# Restorative potential of (−)-epicatechin in a rat model of Gulf War illness muscle atrophy and fatigue

**DOI:** 10.1038/s41598-021-01093-w

**Published:** 2021-11-08

**Authors:** Israel Ramirez-Sanchez, Viridiana Navarrete-Yañez, Alejandra Garate-Carrillo, Modesto Lara-Hernandez, Judith Espinosa-Raya, Aldo Moreno-Ulloa, Benjamin Gomez-Diaz, Ana Lilia Cedeño-Garcidueñas, Guillermo Ceballos, Francisco Villarreal

**Affiliations:** 1 UCSD School of Medicine, 9500 Gilman Dr. BSB4028, La Jolla, CA 92093-0613J USA; 2 Seccion de Estudios de Posgrado e Investigacion, Escuela Superior de Medicina, IPN, Mexico City, Mexico; 3 Carrera de Biologia, Facultad de Estudios Superiores, Iztacala, UNAM, Edo. Mex., Mexico; 4 Laboratorio MS2, Departamento de Innovación Biomédica, CICESE, Ensenada, Mexico; 5 Laboratorio Especializado en Metabolómica y Proteómica (MetPro), CICESE, Ensenada, Mexico; 6 Instituto Nacional de Rehabilitacion, Mexico City, Mexico; 7 VA San Diego Health Care, San Diego, CA USA

**Keywords:** Metabolic disorders, Metabolomics

## Abstract

We examined in a rat model of Gulf War illness (GWI), the potential of (−)-epicatechin (Epi) to reverse skeletal muscle (SkM) atrophy and dysfunction, decrease mediators of inflammation and normalize metabolic perturbations. Male Wistar rats (n = 15) were provided orally with pyridostigmine bromide (PB) 1.3 mg/kg/day, permethrin (PM) 0.13 mg/kg/day (skin), DEET 40 mg/kg/day (skin) and were physically restrained for 5 min/day for 3 weeks. A one-week period ensued to fully develop the GWI-like profile followed by 2 weeks of either Epi treatment at 1 mg/kg/day by gavage (n = 8) or water (n = 7) for controls. A normal, control group (n = 15) was given vehicle and not restrained. At 6 weeks, animals were subjected to treadmill and limb strength testing followed by euthanasia. SkM and blood sampling was used for histological, biochemical and plasma pro-inflammatory cytokine and metabolomics assessments. GWI animals developed an intoxication profile characterized SkM atrophy and loss of function accompanied by increases in modulators of muscle atrophy, degradation markers and plasma pro-inflammatory cytokine levels. Treatment of GWI animals with Epi yielded either a significant partial or full normalization of the above stated indicators relative to normal controls. Plasma metabolomics revealed that metabolites linked to inflammation and SkM waste pathways were dysregulated in the GWI group whereas Epi, attenuated such changes. In conclusion, in a rat model of GWI, Epi partially reverses detrimental changes in SkM structure including modulators of atrophy, inflammation and select plasma metabolites yielding improved function.

## Introduction

Gulf War illness (GWI) afflicts ~ 30% of the US military personnel who served in the 1990–1991 first Persian Gulf War. Symptoms include cognitive deficits, muscle pain, weakness, exercise intolerance and fatigue that is still present after 25 + years^1,2^. Suspect causal agents include the compounds pyridostigmine (PB), permetrim (PM) and N,N-diethyl-m-toluamide (DEET) used as protectants against nerve gases and insects^2–4^. We recently reported a study in which young male rats were provided PB, PM and DEET at equivalent human doses and physical restraint (to induce stress) for 3 weeks^5^. In GWI animals, gastrocnemius weight was 35% lower vs. controls, which correlated with decreases in myofiber area, limb strength and treadmill time/distance. In GWI rats, SkM fiber type relative abundance changed towards the slow type I. Muscle wasting pathway proteins were upregulated while those that promote growth decreased. Decreases in SkM citrate synthase activity, ATP levels and increases in protein carbonyls (indicator of oxidative stress; OS) also suggested the development of mitochondrial dysfunction (MD) results that were further supported by data derived from proteomic analysis of SkM which documented alterations in mitochondrial and metabolic pathways. Thus, the sum of changes suggest that a major cause of fatigue may follow detrimental changes in SkM structure/function^5^.

In GWI Veterans, studies have detected a prolongation of SkM phosphocreatine recovery time after a bout of lower limb exercise lending support to the MD hypothesis^4^. The analysis of white blood cells from GWI Veterans has also detected higher level of mitochondrial DNA mutations vs. controls^6^. It is well established that MD increases OS and promotes the development of an inflammatory milieu^7^. In support of this concept, the untargeted metabolomic analysis of plasma samples of GWI patients demonstrated an altered profile of phospholipids compatible with inflammation^8^. Furthermore, GWI Veteran cytokine plasma levels are higher vs. controls^9^.

We have reported on the stimulatory effects of the flavanol (−)-epicatechin (Epi), which is abundantly present in cacao, on SkM structure/function^10^. In cultured cells, Epi stimulates multiple markers of SkM cell differentiation and growth^11^. Healthy young mice treated with 1 mg/kg of Epi BID by gavage demonstrate enhanced SkM function and ATP levels^10^. In aged mice, Epi restores reactive oxygen species buffering systems and reduces OS in heart and SkM leading to improved function^12^. High flavanol cocoa significantly reduces the plasma levels of cytokines and protein carbonyls in older human subjects^13^. In a pilot study in patients with Becker muscular dystrophy, multiple serum markers of SkM improved with 8 weeks of 100 mg/day of Epi treatment^14^. However, no studies have examined the potential of Epi to reverse GWI associated detrimental changes in SkM and modulators of inflammation.

The objective of this study was to examine the potential restorative effects of Epi using an established rat model of GWI on SkM structure and function. Furthermore, we examined the effects of treatment on atrophy related pathways as well as in the metabolomic and inflammatory cytokine profile of plasma samples.

## Results

**Figure 1:**
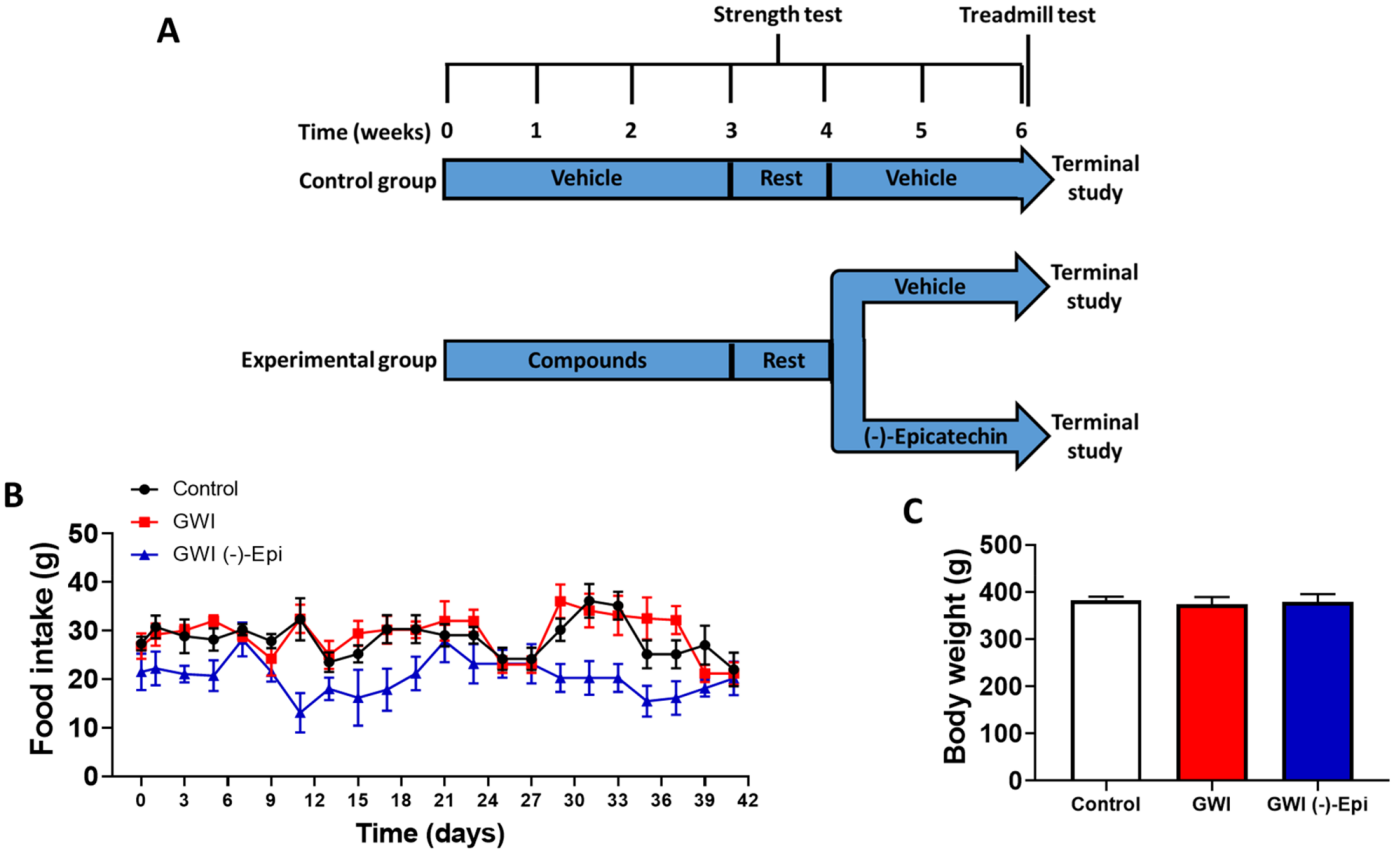
General experimental design describing period of time for controls and experimental groups (GWI and GWI (−)-Epi), and time points in which compounds or vehicle were administrated as well as strength and treadmill test were developed **(A)**. **(B)** Reports on the food intake during the total period of time of the study. **(C)** Reports on the body weight of the animal at the final study time point.

**Figure 2:**
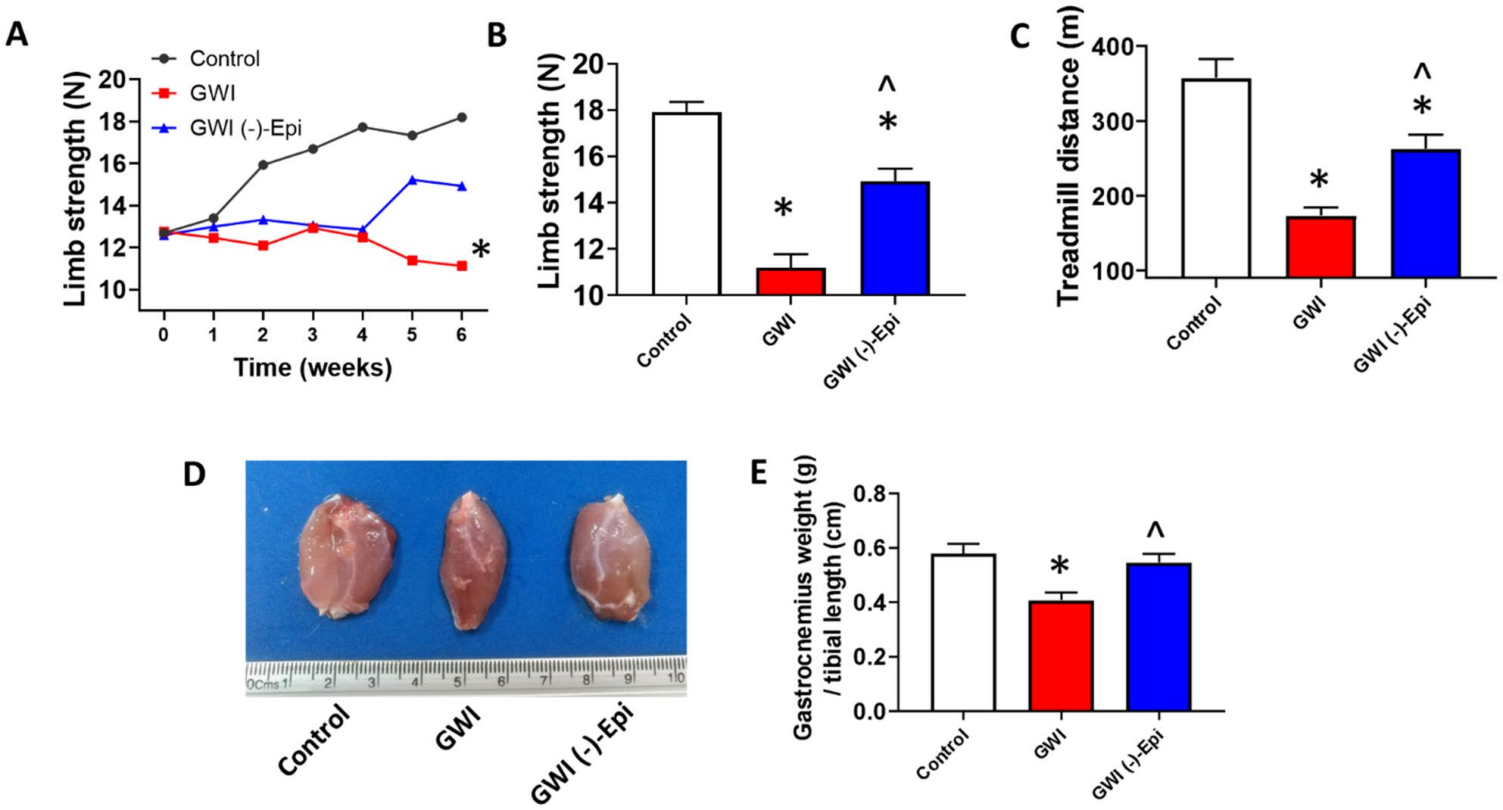
Restorative effects of (−)-epicatechin (Epi) on GWI rat muscle mass and function. Front limb strength recorded weekly **(A)** and at the final (6 week) recording **(B)** in control (n = 9), GWI (n = 5) and GWI-Epi (n = 5) rats. **(C)** Reports on the treadmill test results (time and total distance respectively). **(D,E)** Illustrate and report on weight recorded for gastrocnemius muscles. *p < 0.05 vs. control, ^p < 0.05 vs. GWI by ANOVA and by t-test.

**Figure 3:**
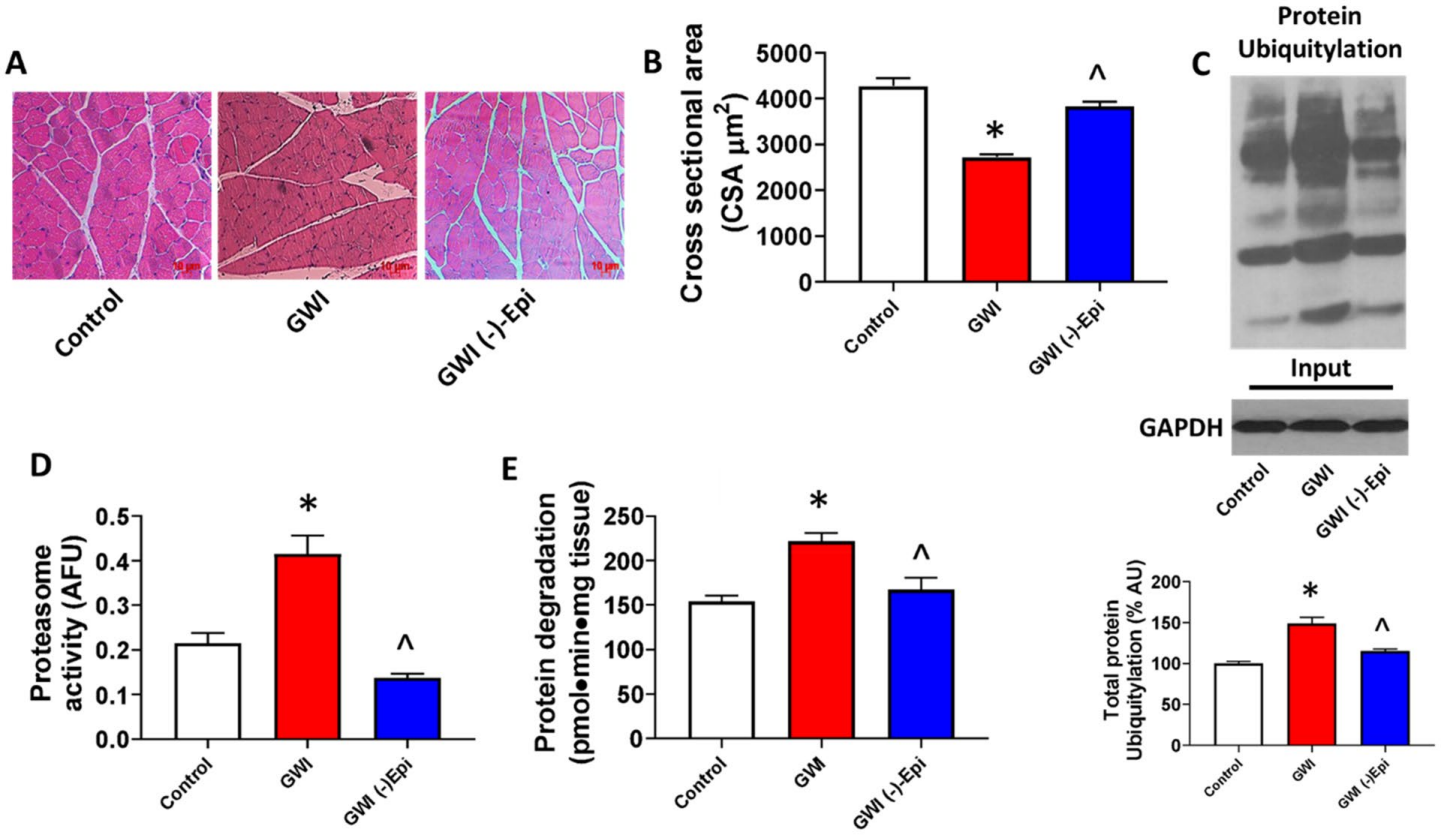
Effects of (−)-epicatechin (Epi) on GWI myofiber area, muscle protein ubiquitylation and degradation. **(A,B)** Images recorded from muscle stained with hematoxilin and eosin (scale bar = 50 µm) and reports on myofiber area measured by image analysis in control (n = 9), GWI (n = 5) and GWI-Epi (n = 5) rats. **(C)** An immunoblot image illustrating enhanced levels of muscle total protein ubiquitylation and reports on the relative quantification. **(D)** Reports on the quantification of proteasome activity (arbitrary fluorescent units = AFU) and **(E)** Reports on protein degradation by tyrosine release in gastrocnemius muscles *p < 0.05 vs. control, ^p < 0.05 vs. GWI by *p < 0.05 by t-test.

Food intake for the duration of the study was similar (i.e., not statistically different) between animal groups as was the final body weight recorded (Fig. 1B,C). Figure 2A plots average once/week recorded front limb strength data. While control animals demonstrated an increase in strength at the 3-week time point, GWI animals demonstrated a steady progressive decline that was significantly different in weeks 3–6. Treatment with Epi, induced a partial recovery of muscle strength in comparison with GWI group. The final strength recorded at week 6 is plotted in panel B. Control animals averaged ~ 18 N whereas GWI animals decreased to ~ 11 N. Epi was able to partially restore strength to ~ 15 N. Treadmill distance is reported in panel C with control animals demonstrating a total distance covered of ~ 360 m while a significant decrease to ~ 180 m occurred in GWI animals. Epi was able to partially restore distance to ~ 275 m. Gastrocnemius mass/weight was also altered (D, E). GWI rats decreased gastrocnemius mass ~ 25% while Epi restored muscle weight to become similar to controls. Figure 3 reports on changes in myofiber cross-sectional area (A, B) which, declined by ~ 35% in the GWI group. Epi restored myofiber cross-sectional area, while nuclei numbers remained unchanged (data not shown). Panel C denotes increases in GWI gastrocnemius total protein ubiquitylation vs. controls where Epi treatment was able to normalize it. Proteasome activity and protein degradation (tyrosine release) also increased in GWI rats (D, E) and Epi treatment was able to restore them to normal levels.

**Figure 4:**
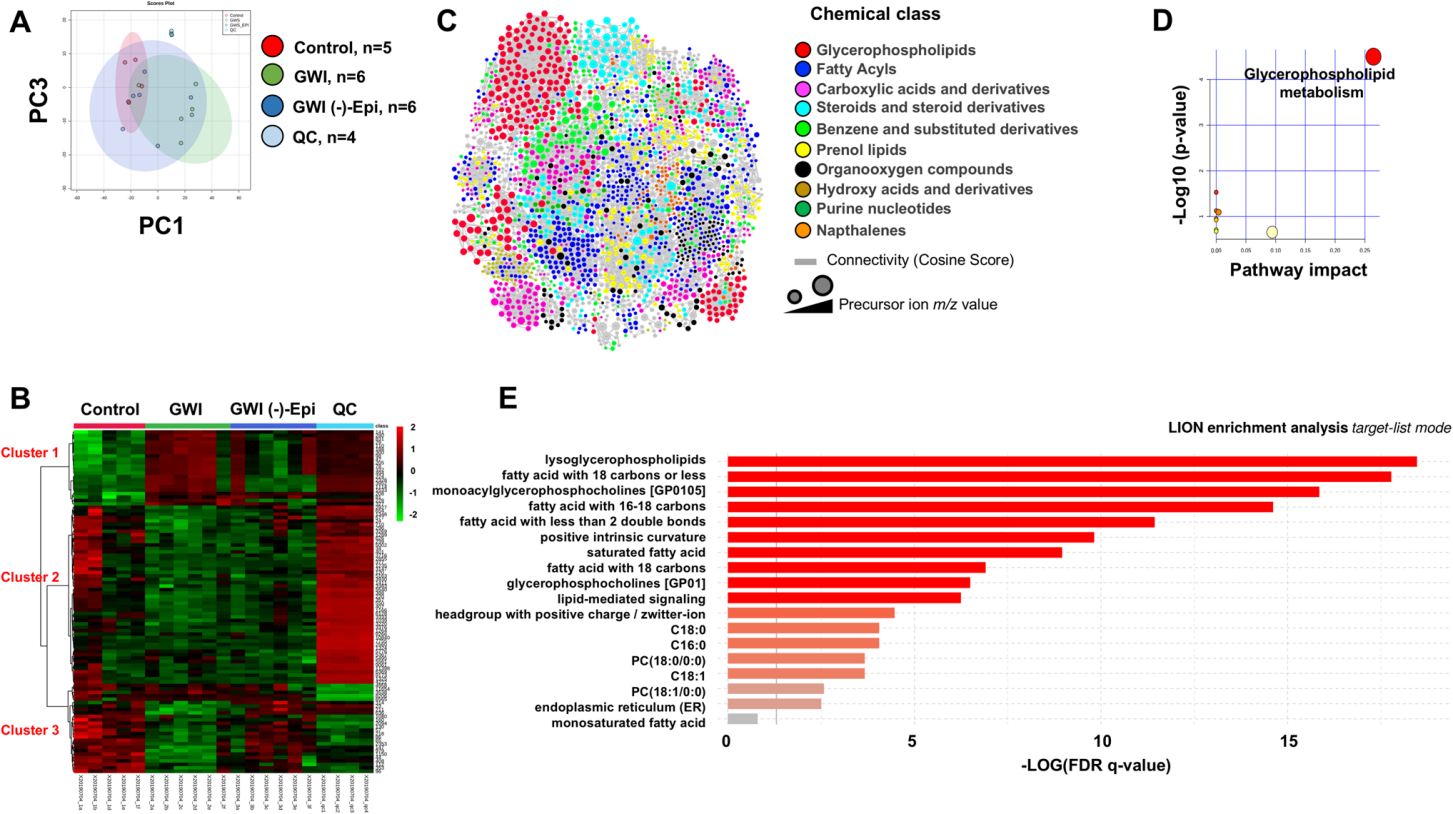
Untargeted metabolomics and chemoinformatic analyses of rat plasma. **(A)** Principal Component Analysis (PCA) on rat serum metabolomes. Data was log transformed without scaling. Shade areas depict the 95% confidence intervals. **(B)** Heat map of the top 100 metabolites ranked by ANOVA test. For hierarchical clustering analysis, Euclidean distance and ward linkage were used as methods. **(C)** Rat serum molecular network colored by the 10 most abundant metabolite chemical classes annotated by Classyfire. Each node represents a unique molecule and edges connecting nodes indicate structural similarity (according to a cosine score). **(D)** Pathway analysis of the up-modulated metabolites by toxicants in rat serum. Circles represent matched pathways. The color and size of the nodes are based on the adjusted p-value and pathway impact value, respectively. **(E)** Enrichment analysis of the up-modulated lipids by toxicants in rat serum using the “target-list mode” in LION/Web. The gray vertical line denotes the cut-off value of significant enrichments (q < 0.05). *QC* quality control samples.

**Figure 5:**
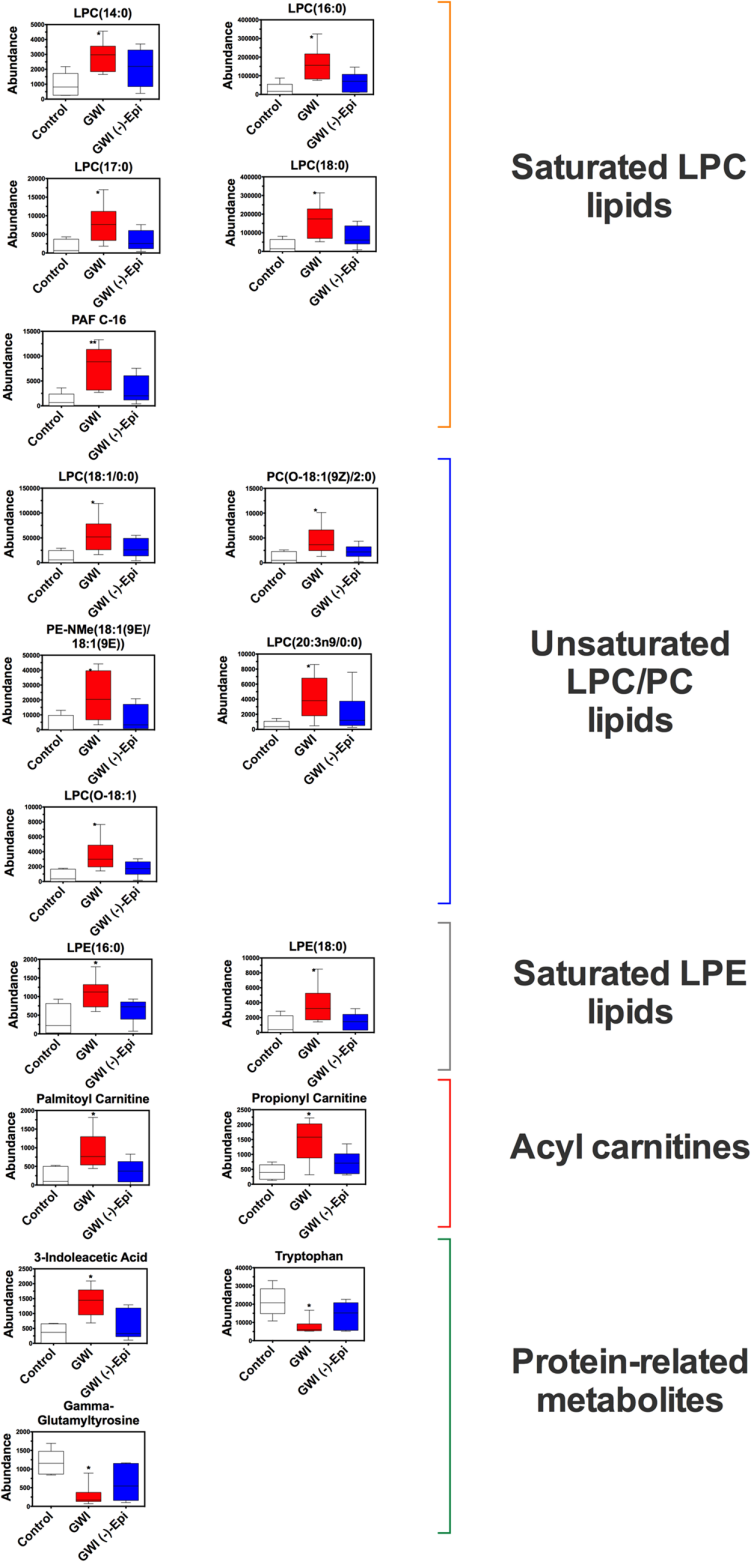
Effects of (−)-epicatechin (Epi) on GWI rat plasma metabolites. Results are expressed as median and 10–90 percentile and visualized as box plots. *p < 0.05 vs. control, **p < 0.01 vs. control, Kruskal–Wallis–Dunn’s multiple comparison post hoc test.

From plasma metabolomics, peak extraction and filtering by XCMS generated 9367 aligned features or potential metabolites among all LC-MS^2^ datasets. To determine global patterns of changes on plasma metabolomes, we performed a PCA analysis on such aligned features using Metaboanalyst 4.0. The tight clustering of QC samples demonstrated a reproducible LC-MS^2^ methodology (Fig. 4A). The first and third principal components revealed a clear separation of control and GWI groups, while rats treated with Epi were clustered between both groups (Fig. 4A). Using a heatmap representation and hierarchical clustering analysis, as another visualization tool, we found specific patterns between datasets using the top-100 most significant features based on ANOVA test (Fig. 4B). At the cluster 1 (top) and 3 (bottom) of the heatmap, we noted a more-alike metabolic pattern between control and GWI + Epi groups, while in cluster 2 (middle) GWI + Epi group was more similar to GWI group than to control group. To determine the identity of the aligned features among groups, we analyzed the LC-MS^2^ datasets using GNPS, which enables online dereplication (MSI level 2) against public spectral libraries and in silico metabolite structural predictions (MSI level 3). A molecular network with 6149 features (containing MS^2^) was created wherein each node represents a unique molecule and edges connecting nodes indicate structural similarity (according to a cosine score) (Fig. 4C). Within the network, 339 (272 unique metabolites, excluding duplicates and contaminants) nodes (5.5%) were matched to the reference spectra of GNPS library (Supplementary Table 2). The top-10 most representative chemical classes, according Classyfire’s ontology, are shown with a distinct color. Of the 272 putatively identified metabolites, only 39 were found differentially abundant among groups based on Kruskal–Wallis test (p < 0.05). To facilitate the understanding of the data analysis, we initially focused on metabolite differences between GWI and control cohorts (post hoc test, p > 0.05). In this regard, 20 metabolites presented with increased abundance (fold change range 1.4–11.3), while 19 presented with reduced abundance (fold change range 0.1–0.55) in the GWI group vs. controls (Supplementary Table 3). To delineate the pathways affected in the GWI group vs. control group, we performed a pathway enrichment analysis using the list of up-regulated metabolites. The most significant pathway was glycerophospholipid metabolism (Fig. 4D). Further enrichment analysis exclusively on lipid metabolites by the LION bioinformatic tool, revealed an overrepresentation of lysoglycerophospholipids and fatty acids with 18 carbons or less (Fig. 4E). No significant enriched pathways were found when using the down-regulated metabolites in the GWI group vs. control group. Notably, Epi treatment attenuated the increase in abundance of various chemical types of lipid metabolites noted in the GWI animals vs. control group (Fig. 5). Likewise, protein catabolism-linked metabolites dysregulated in the GWI vs. control group, were modulated by Epi treatment (Fig. 5). However, Epi treatment did not attenuate or restore the levels of pantothenic acid, deoxycytidine, pyridoxine, tyrosine and valine.

**Figure 6:**
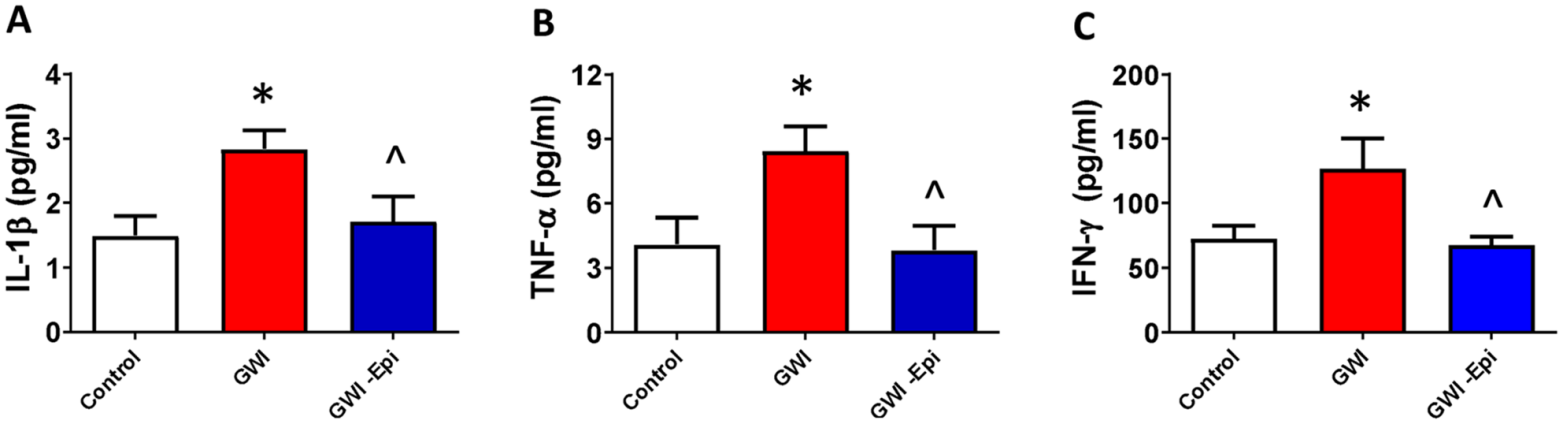
Effects of (−)-epicatechin (Epi) on pro-inflammatory cytokines levels in GWI rat plasma. Quantification of IL-1β **(A)**, TNF-α **(B)** and IFN-γ **(C)** plasma levels in control, GWI and GWI-Epi animals (n = 6/group). *p < 0.05 vs. control, ^p < 0.05 vs. GWI by ANOVA.

**Figure 7:**
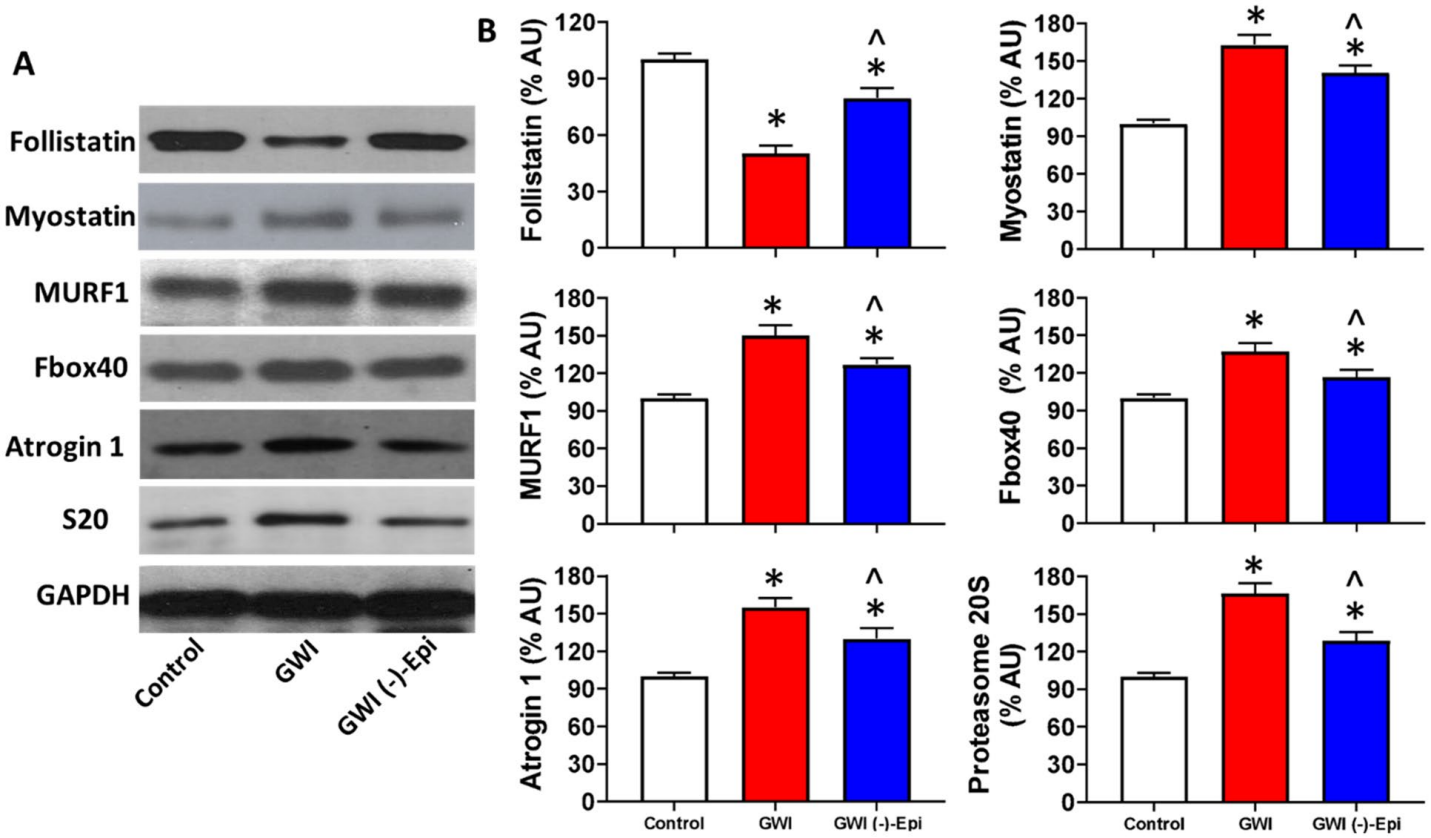
Beneficial effects of (−)-epicatechin (Epi) on modulators of GWI muscle mass. **(A)** Illustrates a representative image of Western blots and (**B**) reports on relative changes in protein levels of gastrocnemius follistatin, myostatin, Murf1, Fbox40, atrogin1 and proteasome subunit 20 (S20) in control, GWI and GWI-Epi animals (n = 6/group). *p < 0.05 vs. control, ^p < 0.05 vs. GWI by t-test. *AU* arbitrary units.

**Figure 8:**
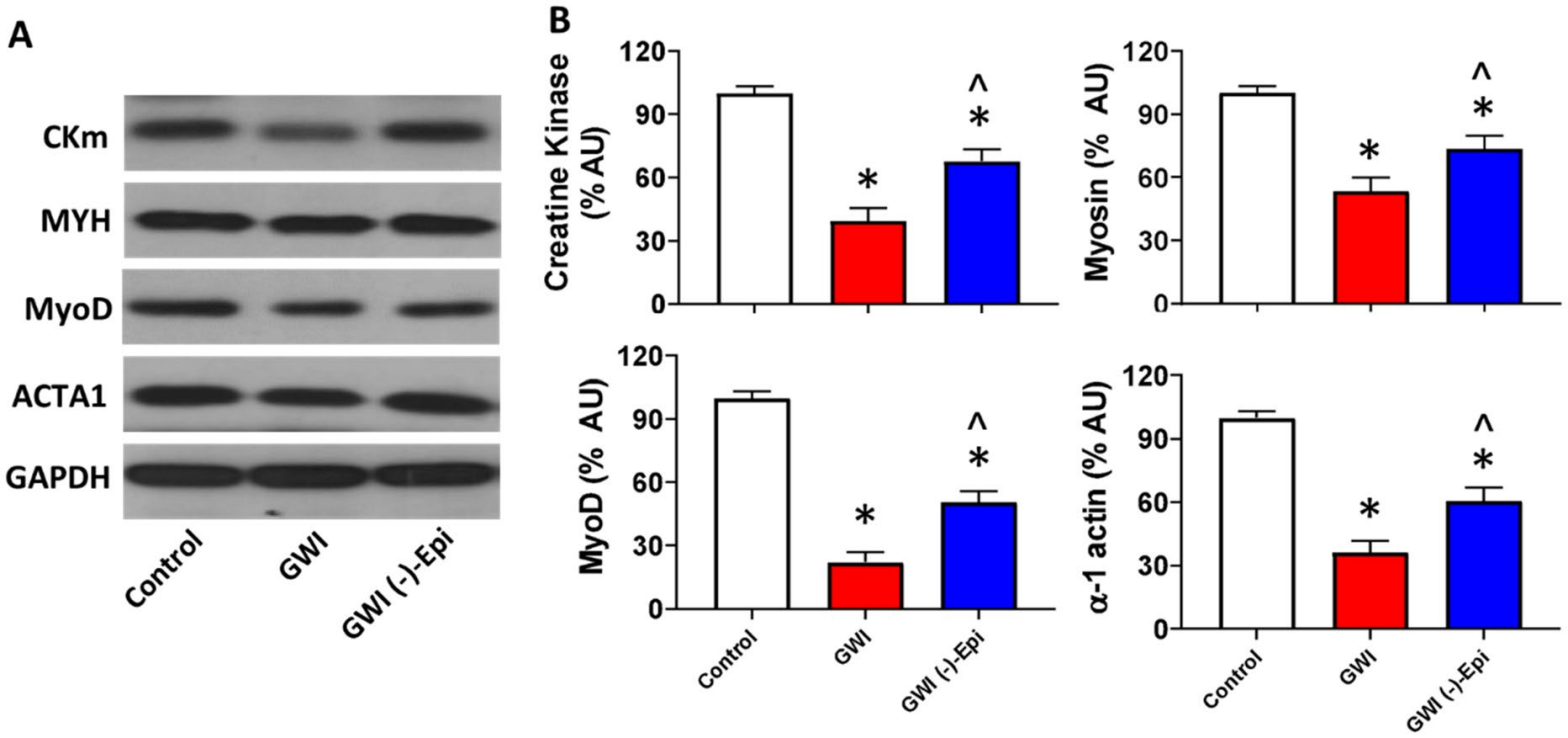
Restorative effects of (−)-epicatechin (Epi) on GWI muscle constitutive proteins. **(A)** Illustrates a representative image of Western blots and **(B)** reports on relative changes in protein levels of gastrocnemius muscle creatine kinase, myosin heavy chain 2a, MyoD, and α1-actin in control, GWI and GWI-Epi animals (n = 6/group). *p < 0.05 vs. control, ^p < 0.05 vs. GWI by t-test. *AU* arbitrary units.

**Figure 9:**
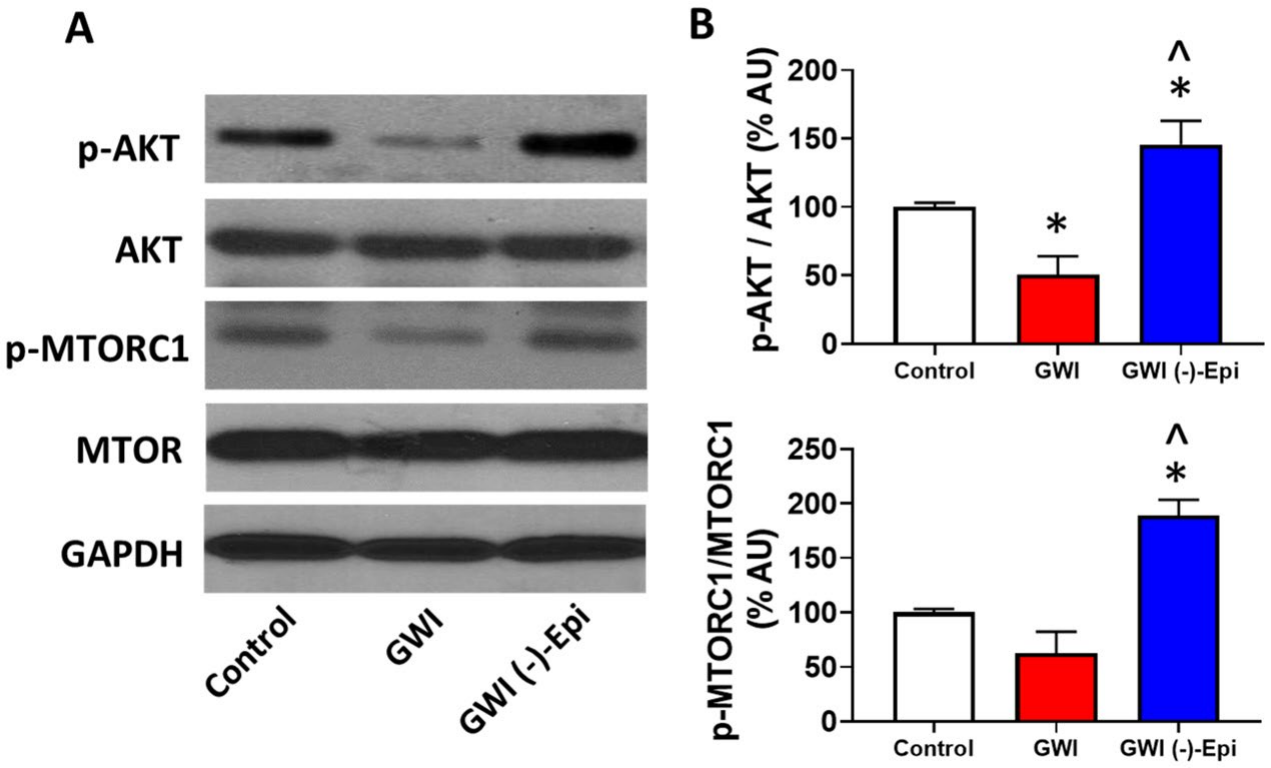
Effects of (−)-epicatechin (Epi) on GWI muscle activation of AKT and MTORC1, two major components of the protein synthesis pathway. (**A**) Illustrates a representative image of Western blots of AKT phosphorylation at Ser473 and MTORC1 at Ser2448 (activation sites); (**B**) reports on phosphorylation activation relative changes in protein levels of gastrocnemius muscle p-AKT and p-MTORC1 in control, GWI and GWI-Epi animals (n = 6/group). *p < 0.05 vs. control, ^p < 0.05 vs. GWI by t-test. *AU* arbitrary units.

The assessment of plasma pro-inflammatory cytokines demonstrated that IL-1β, TNF-α and IFN- levels approximately doubled in GWI animals and Epi treatment was able to normalize them to become comparable to controls (Fig. 6A–C). Figure 7 reports on the assessment of changes in relative protein levels for regulators of muscle growth (follistatin, myostatin) and modulators of SkM atrophy including MURF1, Fbox40, atrogin 1 and proteasome S20. Significant decreases in relative protein levels were noted for follistatin in GWI samples whereas the rest of the modulators were significantly upregulated vs. controls. Epi treatment was able to partially restore protein levels to become closer to controls. Figure 8 shows levels of SkM constitutive proteins (myosin heavy chain, α1-actin and creatine kinase) or of MyoD which can modulate muscle development. GWI demonstrated a significant downregulation of all endpoints which were partially restored with Epi treatment. Figure 9 denotes the effects of Epi on GWI muscle activation/phosphorylation of AKT and MTORC1, two major components of the protein synthesis pathway in skeletal muscle and other tissues. GWI shows decreased protein phosphorylation, while Epi treatment significantly augments activation.

## Discussion

Unique findings from this study provide supportive, pre-clinical evidence for the therapeutic potential of Epi to improve SkM function in GWI. Epi treatment of GWI rats yielded partial restorative changes in SkM mass, strength and endurance while reducing protein degradation and plasma inflammatory cytokines. Plasma metabolome profiles were dysregulated in GWI rats and Epi induced a shift towards controls in select metabolites.

There is precedent for the use of dietary supplements in the experimental treatment of GWI. Kodali et al., reported that treatment for 30 days with curcumin yielded better cognitive and mood function in GWI rats^15^. In brain samples, treatment was associated with enhanced expression of antioxidant and mitochondrial genes, neurogenesis modulators and diminished inflammation. Shetty et al., reported in a rat model of GWI that luminol reinstates redox homeostasis, improves cognition, mood and neurogenesis and alleviates neuro- and systemic inflammation^16^. Joshi et al., reported that treatment with the dietary supplement oleoylethanolamide led to improved cognition, reduced fatigue and disinhibition-like behavior in GWI mice^17^. The same author also reported that the use of nicotinamide riboside reduces neuroinflammation in a GWI mouse model and improved fatigue like behavior^18^. While these studies have mainly focused on neurological endpoints, no studies have yet published on the effects of treatment on SkM structure and function.

The consumption of flavonoids, which are found in plants and their products is associated with healthy effects. Examples of flavonoids evaluated for their healthy actions include those derived from tea (catechins) and from apples and onions (quercetin). Cacao seeds contain by weight the highest amount of flavonoids found in natural products with Epi, being the most predominant form^19^. Intriguing supportive evidence comes from Kuna Indians who live in the San Blas Islands off Panama who, consume every day a “home-made” cacao beverage and their death rates for cardiovascular, cancer and diabetes (amongst others) are a small fraction of the US population^20^. Recent population studies have reported the beneficial effects of consuming modest amounts of cocoa or dark chocolate on many forms of cardiovascular disease with a meta-analysis reporting on ~ 40% overall reduction in cardiometabolic risk^21,22^.

We and others have characterized the stimulatory effects of cocoa and Epi on SkM structure/function. In a study using normal, one year old male mice, 2 weeks of Epi treatment yielded improved SkM structure/function as per increased treadmill performance, myofiber fatigue, capillarity and myofiber cross sectional area^10^. In a separate study, we examined changes in protein levels of molecular modulators of SkM growth and differentiation. Two weeks of Epi treatment decreased myostatin and increased markers of SkM growth and differentiation in normal, 26-month old male mice^12^. In human subjects we also evaluated the effects of 7 days of Epi treatment (25 mg of Epi in capsules BID). Epi increased hand grip strength and the ratio of plasma follistatin/myostatin^23^. We implemented a proof-of-concept study in heart failure patients in which treatment with high flavanol cocoa for 3 months yielded improved modulators of SkM regeneration (MyoD) and growth (follistatin), as did indicators of sarcomeric microstructural integrity^24^. Most recently, we reported on the effects of 3-month supplementation with high flavanol cocoa on exercise capacity in normal sedentary middle age subjects^25^. High flavanol cocoa yielded a stimulatory effect on exercise capacity and work as determined by bicycle ergometry. In a similar study in older subjects, functional indicators of frailty such as a 6-min walk test improved after high flavanol cocoa supplementation. Improvements were also accompanied by decreased plasma levels of markers of OS and inflammation^13^. In patients with peripheral artery disease, high flavanol cocoa supplementation for 3 months led to a significant increase in the 6-min walk test while increasing calf perfusion and capillarity^26^ and in Becker muscular dystrophy patients, treatment with Epi (50 mg BID for 2 months) led to increases in quadriceps and plasma follistatin levels while myostatin decreased^14^. Markers of SkM regeneration and structure-associated proteins also increased with Epi and exercise testing demonstrated decreased heart rate, maximal oxygen consumption/kilogram and plasma lactate levels. Thus, there is compelling evidence to consider the pre-clinical evaluation of Epi for its possible use in GWI.

Local and systemic inflammation is recognized as an important contributor to the pathology of SkM diseases that leads to atrophy^27^. Many of these diseases evidence a sustained elevation of circulating pro-inflammatory cytokines as seen in sarcopenic individuals. Increases in IL-1, IL-6, and TNF-α, have been associated with SkM wasting and weakness^28^. In vivo infusion of TNF-α, IL-1, or IL-6 stimulates myofiber proteolysis in rats, resulting in muscle wasting^29–32^. In the study by Frost et al., it was reported that infusion of TNF-α increased mRNA expression levels of stimulators of atrophy such as atrogin-1, Fbox40 and MURF1. TNF-α also inhibits the PI3K/Akt/mTOR muscle growth pathway^33^. Cytokine action on SkM is largely mediated by the NF-kB pathway that upregulates atrophy modulators while also further facilitating the transcription of proinflammatory cytokines that can further their effects by acting as autocrine or paracrine factors^28^. In GWI Veterans, elevated plasma cytokines and other markers of inflammation^9^ have been reported and associate with neurological symptoms and fatigue. These observations suggest that the use of safe and effective agents capable of suppressing cytokine effects may be suitable for the treatment of SkM atrophy and fatigue.

There is extensive pre-clinical and clinical precedent for the consumption of flavonoid containing foods or pure flavonoids to exert anti-inflammatory actions. As noted above, we reported on the capacity of high flavanol cocoa supplementation to reduce plasma IL-6 and TNF-α (p = 0.06) levels in older, frail subjects. Here, we report that in GWI rats, plasma levels of IL-6, TNF-α and IFN- essentially doubled vs. controls and Epi was able normalize such changes.

Although proinflammatory cytokines levels were reversed by Epi treatment, which is important in the recovery of muscle mass in the setting of atrophy, we observed only a partial recovery in muscle mass and function, which may be related to a partial reduction in myostatin, a key negative regulator of muscle growth. Another possible explanation for this discrepancy, could be the severe impact on mitochondrial function as we reported in our previous study^5^ which is important in the bioenergetics of muscle and thus, the recovery of muscle mass and function. Interestingly, we observed no differences in food intake and body weight between animal groups while GWI rats demonstrate reduced muscle weight. In our previous study, we reported comparable animal weights between controls and GWI rats^5^. While muscle weight was reduced, the content of abdominal fat was increased. The results from the current study suggest that the recovery of muscle weight observed with Epi is likely accompanied by reduced levels of abdominal fat.

Using mass spectrometry-based untargeted metabolomics, multivariate statistics and exhaustive chemoinformatic analysis, we were able to profile the rat plasma metabolome and analyze global and specific differences between groups. PCA revealed differences between GWI and control rats' metabolome, while Epi-treated rats clustered between both groups, suggesting partial protection by Epi from the harmful effects of the GWI related chemical agents. Through molecular networking^34^ and in silico annotation tools^35^ we identified altered metabolites from various chemical classes^36^, ranging from amino acids to lipids, which were differentially abundant between control and GWI groups. Our study identified plasma metabolic changes in GWI rats that are similar to those reported by others in rodents and^37^ in Gulf War Veterans^8^ as in the case for increases in phosphocholine- and phosphoethanolamine-containing lysoglycerophospholipids, and acylcarnitines. Although those lipid metabolites are associated with a diverse array of physiological effects, we believe that they are increased as a result of systemic inflammation in GWI rats. Several reports indicate a relationship among lysoglycerophospholipids, other types of phospholipids (e.g., PAF-C16), and inflammatory cytokines levels (i.e., TNF-α, IL-1β, IFN-) in various organs^38–41^. That agrees with our study showing increased abundance of those two groups (lipids and proteins) of potent inflammatory mediators in the GWI rats. Remarkably, some animal studies suggest a critical role for PAF-C16 and TNF-α in evoking necrosis and dysfunction on SkM cells^42,43^, thereby allowing us to infer a plausible mechanistic route that leads to a muscle injury in GWI rats via overproduction of PAF-C16 and TNF-α. The role of other type of phospholipids in mediating SkM damage remains to be carefully evaluated.

Acyl carnitine metabolites are produced by incomplete fatty acid β-oxidation and their pool in plasma could be derived from various organs or tissues, whereby SkM is believed to be a major contributor^44^. The length of the carnitine acyl chain may reflect their metabolic origin. In contrast, short-chain carnitines are associated with glucose, amino acids, and fatty acid degradation^45^, while medium to long-chain carnitines are mostly linked to fatty acid metabolisms^44,46^. Worth mentioning, some in-vitro reports indicate that long-chain acylcarnitine metabolites (> C14 length) can trigger oxidative stress and inflammation in SkM cells^47,48^. Here, we noted increased levels of a short (C3) and long-chain (C16) acyl-carnitine in GWI rats, which in conjunction with the observed increased levels of PAF-C16, points towards a pool of lipid metabolites contributing to SkM damage in GWI rats. Due to our untargeted metabolomics analysis approach, we could also capture another metabolic view of the plasma rat metabolome. For instance, we report metabolic changes linked to dysregulated protein metabolism. Low plasma levels of tryptophan accompanied by increased metabolite levels of 3-indole acetic acid were noted, indicating augmented tryptophan catabolism^49^. That finding correlates with the increased SkM degradation (e.g., lower mass) found in the GWI rats. Gamma glutamyl-tyrosine presented reduced abundance in GWI rats, and although it is a dipeptide, its precise biological role is not understood. Notably, Epi treatment led to partial restoration in the abundance of all these metabolites, while some metabolites were not modulated by the flavanol hinting at a partial protective effect of Epi against GWI chemicals. Interestingly, low tryptophan levels have been reported in the metabolome of severely ill COVID-19 patients and in those suffering from chronic fatigue syndrome which somewhat resembles GWI symptoms^50^.

In vitro studies have examined on elucidating the molecular mechanisms by which Epi stimulates myogenesis and muscle growth. Kim et al., reported that Epi treated C2C12 myoblasts exhibited the enhanced expression of MyoD and myogenin leading to bigger MHC-positive myotubes^51^ an effect corroborated by our C2C12 studies^11^. Hemdan et al., documented anti-atrophic effects of Epi on C2C12 myotubes subjected to clinorotation^52^. The effects were secondary to the downregulation of atrogin-1 and MuRF1 via the dephosphorylation of ERK. Lee et al., reported that Epi promotes myogenic differentiation in C2C12 cells by increasing the protein levels of MyoD, myogenin leading to increases in MHC^51^. Epi also increased SkM mRNA levels of MyoD and decreased the expression of FoxO3, myostatin and MuRF1^53^. Si et al.^54^ reported that Epi increases the survival rate of aged mice and delays SkM degeneration. In our previous GWI study, we reported the activation of the ubiquitin–proteasome pathway leading to muscle atrophy which was accompanied by elevated levels tyrosine release which, is as a measure of protein degradation^5^. The gastrocnemius of GWI rats demonstrated increases in myostatin as were the levels of MuRF1, MAFbx, atrogin-1, -2 and of proteasome (subunit 20) while those of follistatin decreased. The levels of MyoD, creatine kinase, MHC and α1-actin were also substantially reduced. In this study, we replicate such changes and were able to document Epi’s capacity to partly reverse these alterations leading to essentially, a full recovery of muscle mass. These results confirm our previous report denoting increases in Myf5, MyoD and decreased myostatin in the SkM of Epi treated aged mice^23^. In the present study, the purpose to measuring MyoD was to evidence SkM growth potential. Furthermore, the analysis of AKT and MTORC1 activation (phosphorylation) Epi-induced, strongly support the fact of increasing in protein synthesis in the GWI rat gastrocnemius.

## Conclusions

In conclusion, in a rat model of GWI, Epi reverses adverse changes in recognized modulators of SkM differentiation and growth. As inflammation is recognized as an important driver of muscle atrophy, it is possible that the restorative effects of Epi follow its suppressive effects on modulators of inflammation. These encouraging pre-clinical results support the implementation of clinical trials in GWI Veterans using either high flavanol cocoa or Epi.

## Methods

### Study design and animal model

All experimental protocols were approved by UCSD’s Institutional Animal Care and Use Committee (IACUC) and all methods were carried out in compliance with relevant institutional, Federal and ARRIVE guidelines and regulations. PB, PM, DEET and Epi were obtained from Sigma-Aldrich, Inc. Our GWI animal model is based on a model developed by Hattiangady et al.^55^ with minor modifications. In brief, 3 month old male Wistar rats (n = 15) underwent 3 weeks of exposure to PB (oral gavage 1.3 mg/kg/day) PM and DEET (skin applications 0.13 and 40 mg/kg/day respectively, in 70% ethanol) as well as physical restraint (stress) by placing the animals in a plexiglass holder for 5 min/day^56^. Control animals were given only vehicle and no restraint (n = 15). Animals were then allowed to recover for 1 week. GWI animals (n = 15) were then randomly segregated followed by Epi treatment at 1 mg/kg/day by gavage (n = 8) or water (n = 7) (Fig. 1A). Food intake and body weight were recorded every other day during the exposure and recovery period. After their final functional assessment, animals were euthanized, blood samples and gastrocnemius muscle collected which were weighed.

### Muscle strength

At the beginning of chemical exposure, front limb SkM strength was measured once/week using a grip-strength meter device. Animals are placed on a metal grid where their front paws hold onto a front T-bar. Upon the sudden application of a tail-pull, the animals in a reflex manner, pull the bar and tension is recorded digitally. Weekly measurements were repeated three times and values averaged^5^.

### Treadmill testing

At the end of treatment, treadmill tests were performed as published by us^10^. Rats were initially familiarized with the treadmill device at a slow speed (∼5 m/min) at 10° incline for ~ 10 min for two days previous the final test. Once familiarized, the exhaustion test consisted of a warm-up at 4 m/min for 2 min followed by an increase of 2 m/min every min thereafter. Air jets at the back of the treadmill were used to discourage animals from stopping. Exhaustion was defined as when rats were no longer able to maintain their normal running position and/or were unwilling to run as indicated by contact with the shock grid, which was readily deactivated. Running time was recorded and total distance calculated.

### Histomorphometry

Gastrocnemius muscles were weighed and trimmed before their immediate formaldehyde fixing or freezing for subsequent sectioning or biochemical analysis. Once sectioned, samples were stained with Hematoxilin and Eosin and microscope imaged to digitally quantify nuclei and myofiber cross-sectional area (a total of ~ 450 fibers/group were measured).

### Western blotting

To assess the effects of chemical exposure and Epi treatment on markers of SkM differentiation, growth and atrophy, follistatin, myostatin, MURF1, Fbox40, atrogin 1, proteasome S20, AKT and MTORC1 were determined in samples of gastrocnemius. For regulators of muscle differentiation and growth as well as constitutive proteins, changes in protein levels for MyoD, muscle creatine kinase, myosin heavy chain and α1-actin were determined. Muscle homogenates were prepared and a total of 30 µg of protein were loaded onto a 4–15% gel, electrotransferred, incubated for 1 h in blocking solution (5% non-fat dry milk in tween-Tris buffer saline) and followed by either 3 h incubation at room temperature or overnight at 4 °C with primary antibodies. Primary antibodies were typically diluted 1:1000 or 2000 in Tween buffer plus 0.5% bovine serum albumin or 2% milk-based buffer. Membranes were washed (3 × for 5 min) with Tween buffer and incubated 1 h at room temperature in the presence of species-specific horseradish peroxidase-conjugated secondary antibodies diluted 1:5,000 in blocking solution. Membranes were again washed 3 times with tween buffer and immunoblots developed using chemiluminescence. Band intensities were digitally quantified and normalized using GAPDH as a loading control. Antibodies used included AKT, p-AKT (ser 473), MTORC1, p-MTORC1 (ser 2448), GAPDH, MURF1, Fbox40 (Cell Signaling), atrogin 1, MyoD, follistatin, myostatin (Abcam), ACTA1 ^1-actin^, proteasome S20, myosin heavy chain, (Santa Cruz Biotechnology), muscle creatine kinase (ThermoFisher Scientific).

### Biochemical assays

#### Protein degradation

The protein degradation rate was measured as net tyrosine release from isolated samples. Gastrocnemius samples (10 mg) were pre-incubated for 30 min in Krebs Ringer buffer (NaCl 1.2 mmol/L; KCl 4.8 mmol/L; NaHCO_3_ 25 mmol/L; CaCl_2_ 2.5 mmol/L; KH_2_PO_4_ 1.2 mmol/L and MgSO_4_ 1.2 mmol/L; pH 7.4), supplemented with glucose (5.5 mmol/L), bovine serum albumin (1.0 g/L), insulin (5 U/mL), and cyclohexamide (5 mmol/L), saturated with 95% O_2_/5% CO_2_ gas mixture. Muscles were transferred into a fresh medium of the same composition and incubated for 2 h. At the end of the incubation, medium samples were used to measure tyrosine released by spectrophotometry.

#### Protein ubiquitylation

Lysates were prepared by homogenizing gastrocnemius (15 mg) in a buffer. Samples were centrifuged at 12,000*g* for 10 min and supernatants collected. A total of 30 µg of protein were loaded onto a 4–15% gel and transferred to polyvinylidene fluoride membranes. An antibody (Cell Signaling) was used for the detection of ubiquitinated proteins by Westerns.

#### Proteasome activity assay

Proteasome activity was measured by using a Kit (Abcam) that detects chymotrypsin-like activity using a 7-amino-4-methylcoumarin tagged peptide substrate that generates a fluorescent product in the presence of proteolytic activity. Gastrocnemius lysates (15 mg) were used. Homogenates were centrifuged at 12,000*g* for 10 min and supernatants collected. Proteasome activity was measured with fluorescent substrates of the tagged peptide. The assay was conducted in the absence and presence of the specific proteasome inhibitor MG-132 to determine proteasome-specific activity. Released tagged peptide was measured using a fluorometer at an excitation wavelength of 350 nm and emission of 440 nm.

#### Inflammatory cytokines

For interleukin-1β (IL-1β), tumor necrosis factor-α (TNF-α) and interferon- (IFN-), plasma samples were analyzed by the use of a Milliplex xMAP rat cytokine magnetic-bead Luminex system (Millipore EMD Cat# RecytMAG-65K) following manufacturer instructions.

### Metabolomic analysis

#### Metabolite extraction

Due to limited availability of plasma 5, 6 and 6 animals of the control, GWI, and GWI + Epi groups respectively were processed. For each sample, 50 µL of serum were extracted with 200 µL of a mixture of cold ethanol:methanol at a 50:50 ratio, vortexed for 1 min and centrifuged at 14,000 rpm for 10 min at 4 °C. The supernatant was recovered, transferred to a 1.5 mL Eppendorf tube and the tube placed into a SpeedVac system to evaporate the solvent at room temperature. Extracts were resuspended in 100 µL of a solution of water:acetonitrile at a 95:5 ratio and then centrifuged at 14,000 rpm for 10 min at 4 °C. The particle-free supernatant was recovered for further analysis. Quality control (QC) samples (n = 4) were prepared by mixing equal volumes of all the particle-free supernatants.

#### LC-MS^2^ data acquisition

We employed the LC-MS^2^ methodology previously reported by our group with minor modifications^57^. In brief, plasma samples were loaded into an Eksigent nanoLC400 system (AB Sciex, Foster City, CA, USA) with a HALO Phenyl-Hexyl column (0.5 × 50 mm, 2.7 µm, 90 Å pore size, Eksigent AB Sciex). Metabolites were separated using a gradient elution with 0.1% formic acid in water (A) and 0.1% formic acid in ACN (B) as mobile phases at a constant flow rate of 5 µL/min. The gradient started at 5% B for 1 min, followed by a stepped increased to 100% B over 26 min, held for 4 min with 100% B and solvent composition was then returned to 5% B over 0.1 min. A four-minute pre-run with 5% B was applied between samples to ensure column re-equilibration. A blank sample (1 μL of buffers A:B at a 95:5 ratio) was run between experimental sample injections to minimize potential carryover. The eluate from LC was delivered directly to the TurboV source of a TripleTOF 5600 + mass spectrometer (SCIEX, USA) using electrospray ionization (ESI) under positive mode. ESI source conditions were set as follows: IonSpray Voltage Floating, 5500 V; Source temperature, 350 °C; Curtain gas, 20 psi; Ion source gases 1 and 2 were set to 40 and 45 psi; Declustering potential, 100 V. Data was acquired using information-dependent acquisition (IDA) with high sensitivity mode selected, automatically switching between full-scan MS and MS^2^. The accumulation time for TOF MS was 0.25 s/spectra over the m/z range 100–1500 Da and for MS^2^ scan was 0.05 s/spectra over the *m/z* 50–1500 Da. The IDA settings were as follows: charge state + 1 to + 2, intensity 125 cps, exclude isotopes within 6 Da, mass tolerance 50 mDa, and a maximum number of candidate ions 20. Under IDA settings, the ‘‘exclude former target ions’’ was set as 15 s after two occurrences and ‘‘dynamic background subtract’’ was selected. The manufacturer rolling collision energy (CE) option was used based on the size and charge of the precursor ion using formula CE = m/z × 0.0575 + 9. The instrument was automatically calibrated by the batch mode using appropriate positive TOF MS and MS^2^ calibration solutions before sample loading and after injection of four samples (< 3.5 working hours) to ensure a mass accuracy of < 5 ppm for both MS and MS^2^ data. Instrument performance was monitored during data acquisition by including one QC sample (n = 4) every 4 experimental samples.

#### LC-MS^2^ data processing and analysis

Three complementary informatic approaches were utilized to analyze the LC-MS^2^ datasets. (1) Feature extraction, alignment, normalization, and univariate statistical analysis was performed using XCMS (version 2.7.2) online platform (https://xcmsonline.scripps.edu), (2) MS^2^ spectral data extraction for metabolite identification (Metabolomics Standards Initiative (MSI) classification level 2)^58^ was performed using the Global Natural Products Social Molecular Networking web platform (GNPS, https://gnps.ucsd.edu)^34^, (3) For multivariate statistical analysis and heatmap visualization, Metaboanalyst 4.0 was utilized (https://www.metaboanalyst.ca). For approach one, raw proprietary .wiff files were imported into the XCMS online platform using the parameters specified for the TripleTOF 5600 instrument but with some modifications. The detailed parameters are shown in Supplementary Table 1. The feature areas were normalized by the median fold change. For approach two, *.wiff files were first converted to *.mzML format using ProteoWizard version 3.0^59^ and then imported into the GNPS platform to perform Classical Molecular Networking and automatic structural annotation against GNPS public spectral libraries^34^. To further characterize the features not automatically annotated by spectral matching, we employed the GNPS in silico tool, Network Annotation Propagation (NAP), that uses the output of molecular networking to improve the accuracy of in silico annotations^35^. To provide a more comprehensive chemical overview of the metabolome detected, we integrated the molecular networking, spectral library and NAP in silico annotation outputs, as well as the automated chemical classification through Classyfire into the MolNetEnhancer workflow^60^. Overall, the input parameters were: precursor ion mass and MS^2^ fragment ion tolerance of 0.02 Da. A molecular network was created using a cosine score of 0.7 and at least 4 matched peaks. Spectral matches or automatic metabolite annotation were required to have a cosine score of 0.6 and at least 4 matched peaks. Contaminants (from blank runs) were filtered out before the creation of the molecular networks and automatic metabolite annotation using the GNPS filtering option. The molecular network output was exported to Cytoscape Software version 3.8.2 for visualization^61^. To filter out poor reliable spectral annotations, experimental and theoretical masses for the precursor ion were calculated, as well as the mass accuracy (in ppm) of the comparison. As a cut off, annotations with a mass accuracy greater than 15 ppm were excluded. For approach 3, normalized feature areas obtained with XCMS were exported and submitted as a .txt file to Metaboanalyst 4.0 (https://www.metaboanalyst.ca/) to perform PCA and Heatmap analysis. Data was Log transformed (no scaling) before analysis^62^.

### Data availability

The raw datasets have been deposited on the GNPS/MassIVE public repository^63^ under the accession number MSV000086118. The parameters for classical molecular networking and spectral matching using all datasets (excluding QC samples) are available on the following link: https://gnps.ucsd.edu/ProteoSAFe/status.jsp?task=6534f3e006034998a65afcf943b53ef6. The NAP parameters are available on the following link: https://proteomics2.ucsd.edu/ProteoSAFe/status.jsp?task=03e1ab3fcac24c7f8ba70d48e9b67c07. The MolNetEnhancer output is available on the following link: https://gnps.ucsd.edu/ProteoSAFe/status.jsp?task=a6a795eecfc4491dac5fc4c0a76b8510. The quantitative results generated using the XCMS platform can be accessed after logging into the following link https://xcmsonline.scripps.edu and searching for the job number 1397687.

### Bioinformatic analysis

We initially focused on metabolites differentially abundant between control and GWI cohorts to determine the pathways affected by the chemicals. The chemical names of the annotated (by GNPS spectral matching) differential abundant metabolites were converted to HMDB IDs and submitted as a list to Metaboanalyst 4.0 to perform pathway analysis. The input parameters were: Over Representation Analysis, Hypergeometric Test; Pathway Topology Analysis, Relative-betweeness Centrality; Pathway library, Rattus norvegicus (rat) (KEGG)^64^. In addition, to overcome the limited coverage of KEGG database on the diversity of lipids, we performed an enrichment analysis using the Lipid Ontology (LION) enrichment analysis web application (LION/web) using only the list of metabolites annotated as lipids. The chemical names of the lipids were converted to their standardized name (RefMet nomenclature) using an online tool on https://www.metabolomicsworkbench.org/databases/refmet/name_to_refmet_form.php. Lipids were entered as a target-list using the background list of lipids reported by Molenaar MR, et al.^65^.

### Statistical analysis

For biological experiments, data are reported as mean ± standard error of the mean (SEM). Statistical analysis included one-way analysis of variance (ANOVA) or unpaired t-test as appropriate using Graph Pad Prism 9. Results were considered statistically significant at a value of p < 0.05. For the metabolomics data, features with a fold change ≥ 1.4 or ≤ 1/1.4 and a *p-value* < 0.05 (Welch’s t-test) were considered as differentially abundant when comparing two groups. To assess for differences among multiple groups, Kruskal Wallis followed by post-hoc multiple comparison test (within XCMS) was utilized. To determine the effects of Epi treatment, further inspection of features annotated (MSI, level 2) by GNPS and with a p < 0.05 (post-hoc test in XCMS) was performed in PRIMS 6.0 (GraphPad Software, San Diego, CA). The abundance of selected metabolites was plotted as Box Plots depicting median and 10–90 percentile. Derived from the bioinformatic analysis, we only considered pathways (hypergeometric Test) and LION-terms (Fisher’s exact test) with an adjusted p < 0.05. For multivariate statistical analysis and heatmap visualization, Metaboanalyst 4.0 (https://www.metaboanalyst.ca) was utilized. Principal component analysis (PCA) was used to assess for sample clustering behavior and inter-group variation. Data was Log transformed (without scaling) for PCA and heatmap analysis.

## Supplementary Information

Supplementary Tables.

Supplementary Figures.
