# Supplementary material for: Restorative potential of (−)-epicatechin in a rat model of Gulf War illness muscle atrophy and fatigue
Source: Sci Rep. 2021 Nov 8;11:21861. doi: 10.1038/s41598-021-01093-w (PMC8575952; doi:10.1038/s41598-021-01093-w)
Supplement: Supplementary file 1 — Supplementary Tables. [file 41598_2021_1093_MOESM1_ESM.docx]

**SUPPLEMANTARY INFORMATION**

**Table S1.** Parameters used for TripleTOF5600 LC-MS^2^ datasets in XCMS online platform.

| ------------------------------------------------------------------------ | | |
| --- | --- | --- |
| XCMSOnline version 2.7.2 | | |
| XCMS version 1.47.3 | | |
| CAMERA version 1.34.0 | | |
| ------------------------------------------------------------------------ | | |
| 1. General parameters | |  |
|  | Polarity | positive |
|  | Retention time format | minutes |
| 2. Feature detection | |  |
|  | method : centWave |  |
|  | ppm | 15 |
|  | snthr | 6 |
|  | peakwidth | 15 90 |
|  | mzdiff | 0.01 |
|  | prefilter peaks | 3 |
|  | prefilter intensity | 100 |
|  | noise | 0 |
| 3. Retention time correction | |  |
|  | method : obiwarp |  |
|  | profStep | 1 |
| 4. Grouping |  |  |
|  | method : density |  |
|  | bw | 7 |
|  | mzwid | 0.015 |
|  | minfrac | 0.5 |
|  | minsamp | 1 |
| 5. FillPeaks |  |  |
| 6. Diffreport |  |  |
|  | classes | GWS_rats_wiff |
|  | classes | Control_Rats_wiff |
|  | classes | Quality_Control_GWS |
|  | classes | EPI_GWS_Wiff |
|  | statistical test | Kruskal Wallis |
|  | statistics.threshold.pvalue | 0.01 |
|  | statistics.diffReport.value | into |
|  | statistics.normalization | Median Fold Change |
| Removing Quality_Control_GWS from the statistical analysis | |  |
| Making Coeff. Var. from QC sample: Quality_Control_GWS | |  |
| Finished Running Statistical tests | |  |
| 7. Additional Plots & Statistics | |  |
|  | Running mummichog |  |
|  | Printing MDS plot |  |
|  | Printing static PCA and Select Scaling plot |  |
| 8. Annotation (isotopes & adducts) | |  |
|  | featureAnnotation.CAMERA.annotate | isotopes + adducts |
|  | featureAnnotation.CAMERA.mzabs | 0.01 |
|  | featureAnnotation.CAMERA.ppm | 10 |
|  | featureAnnotation.CAMERA.sigma | 6 |
|  | featureAnnotation.CAMERA.perfwhm | 0.6 |
|  | featureAnnotation.CAMERA.maxcharge | 3 |
|  | featureAnnotation.CAMERA.maxiso | 4 |
|  | featureAnnotation.CAMERA.intensity | into |
| 9. Putative ID's (METLIN) | |  |
|  | identification.METLIN.ppm | 10 |
|  | identification.METLIN.adducts | M+H, M+NH4, M+Na |
|  | Found 1476 total, MS^2 spectra to match |  |

**Table S2.** List of all metabolites annotated by spectral matching against GNPS public spectral libraries.

| **Compound_Name** | **LibMZ** | **SpecMZ** | **Adduct** | **MassDiff** | **MZErrorPPM** | **RT (Min)** | **RT (s)** | **SharedPeaks** | **Instrument** | **Smiles** | **INCHI** | **INCHI_AUX** | **LibraryQualityString** | **#Scan#** | **MQScore** | **TIC_Query** | **InChIKey** | **InChIKey-Planar** | **superclass** | **class** | **subclass** |
| --- | --- | --- | --- | --- | --- | --- | --- | --- | --- | --- | --- | --- | --- | --- | --- | --- | --- | --- | --- | --- | --- |
| 11,22-dimethyl (6E,17E)-3,14-dioxo-8,19-bis({[(2S,3R,4S,5S,6R)-3,4,5-trihydroxy-6-(hydroxymethyl)oxan-2-yl]oxy})-4,9,15,20-tetraoxatricyclo[16.4.0.0?,ï¿½ï¿½]docosa-6,10,17,21-tetraene-11,22-dicarboxylate | 288.144 | 288.143 | [M+NH4]+ | 0.00100708 | 3.49506 | 16.2450333 | 974.702 | 4 | ESI-QFT | N/A | InChI=1S/C13H18O6/c14-6-9-10(15)11(16)12(17)13(19-9)18-7-8-4-2-1-3-5-8/h1-5,9-17H,6-7H2/t9-,10-,11+,12-,13-/m1/s1 | N/A | Bronze | 7740 | 0.74723 | 4997.23 | GKHCBYYBLTXYEV-UJPOAAIJSA-N | GKHCBYYBLTXYEV | Organic oxygen compounds | Organooxygen compounds | Carbohydrates and carbohydrate conjugates |
| 1,4a-dimethyl-9-oxo-7-propan-2-yl-3,4,10,10a-tetrahydro-2H-phenanthrene-1-carboxylic acid | 315.195 | 315.196 | [M+H]+ | 0.00100708 | 3.1951 | 19.5488333 | 1172.93 | 7 | ESI-QFT | N/A | InChI=1S/C20H26O3/c1-12(2)13-6-7-15-14(10-13)16(21)11-17-19(15,3)8-5-9-20(17,4)18(22)23/h6-7,10,12,17H,5,8-9,11H2,1-4H3,(H,22,23) | N/A | Bronze | 10930 | 0.777523 | 15276.2 | MSWJSDLNPCSSNW-UHFFFAOYSA-N | MSWJSDLNPCSSNW | Lipids and lipid-like molecules | Prenol lipids | Diterpenoids |
| 1-(hexadecanoyloxy)-3-hydroxypropan-2-yl-octadec-9-enoate | 612.556 | 612.555 | M+NH4 | 0.0010376 | 1.69388 | 29.3491667 | 1760.95 | 11 | qTof |  |  |  | Bronze | 24397 | 0.768045 | 2661.15 | N/A | N/A | N/A | N/A | N/A |
| (1S,5R,9S,13R)-14-formyl-5,9-dimethyltetracyclo[11.2.1.0Â¹,Â¹ï¿½?ï¿½.0ï¿½?ï¿½,ï¿½?ï¿½]hexadec-14-ene-5-carboxylic acid | 317.211 | 317.211 | M+H | 0 | 0 | 16.7195 | 1003.17 | 7 | Orbitrap | C[C@@](CCC1)(C(CC[C@H]2C3)[C@@]3(CC3)C=C2C=O)C3[C@@]1(C)C(O)=O | InChI=1S/C20H28O3/c1-18-7-3-8-19(2,17(22)23)15(18)6-9-20-10-13(4-5-16(18)20)14(11-20)12-21/h11-13,15-16H,3-10H2,1-2H3,(H,22,23)/t13-,15?,16?,18-,19-,20+/m0/s1 | N/A | Gold | 11236 | 0.63311 | 7176.97 | DIURMAFIAYOVGU-JVLNKKSHSA-N | DIURMAFIAYOVGU | N/A | N/A | N/A |
| (1S,5R,9S,13R)-5,9-dimethyl-14-methylidenetetracyclo[11.2.1.0Â¹,Â¹ï¿½?ï¿½.0ï¿½?ï¿½,ï¿½?ï¿½]hexadecane-5-carboxylic acid | 303.232 | 303.231 | M+H | 0.0010071 | 3.32115 | 22.394 | 1343.64 | 9 | Orbitrap | C[C@@](CCC1)(C(CC[C@@H](C2)C(C3)=C)[C@@]23CC2)C2[C@@]1(C)C(O)=O | InChI=1S/C20H30O2/c1-13-11-20-10-7-15-18(2,16(20)6-5-14(13)12-20)8-4-9-19(15,3)17(21)22/h14-16H,1,4-12H2,2-3H3,(H,21,22)/t14-,15?,16?,18-,19-,20-/m0/s1 | N/A | Gold | 9788 | 0.757141 | 16646.9 | NIKHGUQULKYIGE-TYTHQFKISA-N | NIKHGUQULKYIGE | N/A | N/A | N/A |
| (2-{[2-[docosa-4.7.10.13.16-pentaenoyloxy]-3-(hexadecanoyloxy)propyl phosphonato]oxy}ethyl)trimethylazanium | 808.586 | 808.583 | M+H | 0.0029907 | 3.69871 | 22.638 | 1358.28 | 9 | qTof | CCCCCCCCCCCCCCCC(=O)OC[C@]([H])(COP([O-])(=O)OCC[N+](C)(C)C)OC(=O)CC\C=C/C\C=C/C\C=C/C\C=C/C\C=C/CCCCC | N/A | N/A | Bronze | 26316 | 0.818756 | 32800.1 | MWXAIAMSOXOQJK-JIXVGTEKSA-N | MWXAIAMSOXOQJK | Lipids and lipid-like molecules | Glycerophospholipids | Glycerophosphocholines |
| (2-{[2-hexadecanamido-3-hydroxyoctadec-4-en-1-yl phosphono]oxy}ethyl)trimethylazanium | 703.575 | 703.576 | M+H | 0.00097656 | 1.388 | 25.441 | 1526.46 | 6 | qTof | [H][C@@](COP([O-])(=O)OCC[N+](C)(C)C)(NC(=O)CCCCCCCCCCCCCCC)[C@H](O)\C=C\CCCCCCCCCCCCC | N/A | N/A | Bronze | 25354 | 0.793819 | 62917.6 | RWKUXQNLWDTSLO-GWQJGLRPSA-N | RWKUXQNLWDTSLO | Lipids and lipid-like molecules | Sphingolipids | Phosphosphingolipids |
| (2-{[2-(hexadecanoyloxy)-3-(pentadecanoyloxy)propyl phosphonato]oxy}ethyl)trimethylazanium | 720.554 | 720.555 | M+H | 0.00097656 | 1.35529 | 27.8113333 | 1668.68 | 5 | qTof | [H][C@@](COC(=O)CCCCCCCCCCCCCC)(COP([O-])(=O)OCC[N+](C)(C)C)OC(=O)CCCCCCCCCCCCCCC | N/A | N/A | Bronze | 25498 | 0.825673 | 7921.85 | LLHISNQVRRYJGL-DIPNUNPCSA-N | LLHISNQVRRYJGL | Lipids and lipid-like molecules | Glycerophospholipids | Glycerophosphocholines |
| (2-{[2-[icos-11-enoyloxy]-3-[octadec-9-enoyloxy]propyl phosphonato]oxy}ethyl)trimethylazanium | 814.633 | 814.635 | M+H | 0.0020142 | 2.47248 | 25.361 | 1521.66 | 6 | qTof | CCCCCCCC\C=C/CCCCCCCCCC(=O)O[C@]([H])(COC(=O)CCCCCCC\C=C/CCCCCCCC)COP([O-])(=O)OCC[N+](C)(C)C | N/A | N/A | Bronze | 26369 | 0.845305 | 8917.4 | JLDQDLKBBDQYDE-WWUFLCHTSA-N | JLDQDLKBBDQYDE | Lipids and lipid-like molecules | Glycerophospholipids | Glycerophosphocholines |
| (2-{[2-[icosa-5.8.11.14-tetraenoyloxy]-3-[octadec-9-en-1-yloxy]propyl phosphono]oxy}ethyl)trimethylazanium | 794.605 | 794.605 | M+H | 0 | 0 | 24.8183333 | 1489.1 | 9 | qTof | [H][C@@](COCCCCCCCC\C=C/CCCCCCCC)(COP([O-])(=O)OCC[N+](C)(C)C)OC(=O)CCC\C=C/C\C=C/C\C=C/C\C=C/CCCCC | N/A | N/A | Bronze | 26196 | 0.862421 | 11544.2 | VJNPDLZENXBRLB-MQEDXBOASA-N | VJNPDLZENXBRLB | Lipids and lipid-like molecules | Glycerophospholipids | Glycerophosphocholines |
| (2-{[2-[icosa-5.8.11-trienoyloxy]-3-(octadecanoyloxy)propyl phosphonato]oxy}ethyl)trimethylazanium | 812.616 | 812.613 | M+H | 0.00305176 | 3.75547 | 24.7386667 | 1484.32 | 8 | qTof | CCCCCCCCCCCCCCCCCC(=O)OC[C@]([H])(COP([O-])(=O)OCC[N+](C)(C)C)OC(=O)CCC\C=C/C\C=C/C\C=C/CCCCCCCC | N/A | N/A | Bronze | 26355 | 0.759196 | 6775.24 | QXDDDGCNWRUEFM-UMKMFDOBSA-N | QXDDDGCNWRUEFM | Lipids and lipid-like molecules | Glycerophospholipids | Glycerophosphocholines |
| [2,3-di(heptadecanoyloxy)propyl] 2-(trimethylazaniumyl)ethyl phosphate | 762.601 | 762.601 | M+H | 0 | 0 | 23.0665 | 1383.99 | 5 | qTof |  |  |  | Bronze | 25887 | 0.86322 | 33448.6 | N/A | N/A | N/A | N/A | N/A |
| (2-{[3-(hexadecanoyloxy)-2-[icosa-5.8.11.14.17-pentaenoyloxy]propyl phosphonato]oxy}ethyl)trimethylazanium | 780.554 | 780.553 | M+H | 0.0010376 | 1.32931 | 26.4758333 | 1588.55 | 5 | qTof | CCCCCCCCCCCCCCCC(=O)OC[C@]([H])(COP([O-])(=O)OCC[N+](C)(C)C)OC(=O)CCC\C=C/C\C=C/C\C=C/C\C=C/C\C=C/CC | N/A | N/A | Bronze | 26035 | 0.874452 | 31433.3 | KLTHQSWIRFFBRI-CPFPVJFHSA-N | KLTHQSWIRFFBRI | Lipids and lipid-like molecules | Glycerophospholipids | Glycerophosphocholines |
| (2-{[3-(hexadecanoyloxy)-2-[icosa-5,8,11,14-tetraenoyloxy]propyl phosphonato]oxy}ethyl)trimethylazanium | 804.551 | 804.551 | M+Na | 0 | 0 | 21.8968333 | 1313.81 | 11 | qTof |  |  |  | Bronze | 26277 | 0.699711 | 7245.7 | N/A | N/A | N/A | N/A | N/A |
| "2-((4R)-4-((3R,5R,6S,7S,9S,10R,13R,14S,17R)-3,6,7-trihydroxy-10,13-dimethylhexadecahydro-1H-cyclopenta[a]phenanthren-17-yl)pentanamido)ethane-1-sulfonic acid" | 480.278 | 480.276 | M-2H2O+H | 0.0020142 | 4.19374 | 13.21885 | 793.131 | 11 | qTof | [H][C@@]12[C@H](O)[C@@H](O)[C@]3([H])C[C@H](O)CC[C@]3(C)[C@H]1CC[C@@]4(C)[C@H]2CC[C@@H]4[C@@H](CCC(NCCS(=O)(O)=O)=O)C | "InChI=1S/C26H45NO7S/c1-15(4-7-21(29)27-12-13-35(32,33)34)17-5-6-18-22-19(9-11-25(17,18)2)26(3)10-8-16(28)14-20(26)23(30)24(22)31/h15-20,22-24,28,30-31H,4-14H2,1-3H3,(H,27,29)(H,32,33,34)/t15-,16-,17-,18+,19+,20+,22+,23+,24+,25-,26-/m1/s1" | N/A | Gold | 21929 | 0.737867 | 4279.33 | XSOLDPYUICCHJX-QQXJNSDFSA-N | XSOLDPYUICCHJX | N/A | N/A | N/A |
| (2-aminoethoxy)[2-[icosa-5.8.11.14-tetraenoyloxy]-3-[octadec-11-enoyloxy]propoxy]phosphinic acid | 766.539 | 766.539 | M+H | 0 | 0 | 26.7356667 | 1604.14 | 12 | qTof | [H][C@@](COC(=O)CCCCCCCCC\C=C/CCCCCC)(COP(O)(=O)OCCN)OC(=O)CCC\C=C/C\C=C/C\C=C/C\C=C/CCCCC | N/A | N/A | Bronze | 25925 | 0.704499 | 23332.6 | LJIFHMULTXIFNU-KAMIUYGRSA-N | LJIFHMULTXIFNU | Lipids and lipid-like molecules | Glycerophospholipids | Glycerophosphoethanolamines |
| 2-((heptadec-9-enoyl)oxy)-3-((hexadec-9-enoyl)oxy)propyl (2-(trimethylammonio)ethyl) phosphate | 744.554 | 744.553 | M+H | 0.0010376 | 1.39358 | 27.1858333 | 1631.15 | 5 | qTof | [C@](COP(=O)([O-])OCC[N+](C)(C)C)([H])(OC(CCCCCCC/C=C\CCCCCCC)=O)COC(CCCCCCC/C=C\CCCCCC)=O | N/A | N/A | Bronze | 25684 | 0.872956 | 29473.2 | AEPBKRNQLLRJTA-NYVOMTAGSA-N | AEPBKRNQLLRJTA | Lipids and lipid-like molecules | Glycerophospholipids | Glycerophosphocholines |
| {[2-hexadecanamido-3-hydroxyoctadec-4-en-1-yl]oxy}[2-(trimethylazaniumyl)ethoxy]phosphinic acid | 725.557 | 725.556 | M+Na | 0.00097656 | 1.34595 | 25.4065 | 1524.39 | 8 | qTof |  |  |  | Bronze | 25527 | 0.841182 | 4803.85 | N/A | N/A | N/A | N/A | N/A |
| "2-((R)-4-((3R,5R,6S,7R,8S,9S,10R,13R,14S,17R)-3,6,7-trihydroxy-10,13-dimethylhexadecahydro-1H-cyclopenta[a]phenanthren-17-yl)pent-2-enamido)ethane-1-sulfonic acid" | 514.283 | 514.283 | M+H | 0 | 0 | 13.4103167 | 804.619 | 16 | qTof | C[C@@H]([C@H]1CC[C@]2([H])[C@]1(C)CC[C@@]3([H])[C@@]2([H])[C@@H](O)[C@@H](O)[C@@]4([H])[C@]3(C)CC[C@@H](O)C4)/C=C/C(NCCS(=O)(O)=O)=O | "InChI=1S/C26H43NO7S/c1-15(4-7-21(29)27-12-13-35(32,33)34)17-5-6-18-22-19(9-11-25(17,18)2)26(3)10-8-16(28)14-20(26)23(30)24(22)31/h4,7,15-20,22-24,28,30-31H,5-6,8-14H2,1-3H3,(H,27,29)(H,32,33,34)/b7-4+/t15-,16-,17-,18+,19+,20+,22+,23+,24-,25-,26-/m1/s1" | N/A | Gold | 22768 | 0.847527 | 9082.56 | JZAIXDCPNUAWTQ-KAJAMYNGSA-N | JZAIXDCPNUAWTQ | N/A | N/A | N/A |
| "2-((R)-4-((3R,5S,7S,8R,9S,10S,13R,14S,17R)-3,7-dihydroxy-10,13-dimethylhexadecahydro-1H-cyclopenta[a]phenanthren-17-yl)pentanamido)ethane-1-sulfonic acid" | 500.304 | 500.304 | M+H | 0 | 0 | 15.8058667 | 948.352 | 16 | qTof | [H][C@@]12[C@@H](O)C[C@]3([H])C[C@H](O)CC[C@]3(C)[C@H]1CC[C@@]4(C)[C@H]2CC[C@@H]4[C@@H](CCC(NCCS(=O)(O)=O)=O)C | "InChI=1S/C26H45NO6S/c1-16(4-7-23(30)27-12-13-34(31,32)33)19-5-6-20-24-21(9-11-26(19,20)3)25(2)10-8-18(28)14-17(25)15-22(24)29/h16-22,24,28-29H,4-15H2,1-3H3,(H,27,30)(H,31,32,33)/t16-,17+,18-,19-,20+,21+,22+,24+,25+,26-/m1/s1" | N/A | Gold | 22462 | 0.824015 | 15586.8 | BHTRKEVKTKCXOH-LBSADWJPSA-N | BHTRKEVKTKCXOH | Lipids and lipid-like molecules | Steroids and steroid derivatives | Bile acids, alcohols and derivatives |
| 384 | 355.063 | 355.065 | M+H | 0.00201416 | 5.67268 | 11.7826333 | 706.958 | 8 | qTof |  |  |  | Bronze | 15623 | 0.655538 | 8294.83 | N/A | N/A | N/A | N/A | N/A |
| 3-((heptadec-9-enoyl)oxy)-2-(oleoyloxy)propyl (2-(trimethylammonio)ethyl) phosphate | 772.585 | 772.585 | M+H | 0 | 0 | 28.5635 | 1713.81 | 5 | qTof | [C@](COP(=O)([O-])OCC[N+](C)(C)C)([H])(OC(CCCCCCC/C=C\CCCCCCCC)=O)COC(CCCCCCC/C=C\CCCCCCC)=O | N/A | N/A | Bronze | 25976 | 0.85987 | 2975.76 | CWDLNVPVXRALIK-UNUIOPIBSA-N | CWDLNVPVXRALIK | Lipids and lipid-like molecules | Glycerophospholipids | Glycerophosphocholines |
| 3-hydroxy-2-(tetracosa-11.13.15-trienamido)octadecyl (2-(trimethylammonio)ethyl) phosphate | 811.669 | 811.667 | M+H | 0.00201416 | 2.4815 | 25.4921667 | 1529.53 | 6 | qTof | [H][C@@](NC(CCCCCCCCC/C=C/C=C/C=C/CCCCCCCC)=O)([C@@H](CCCCCCCCCCCCCCC)O)COP([O-])(OCC[N+](C)(C)C)=O | N/A | N/A | Bronze | 26349 | 0.853294 | 9645.83 | BBUSRIKDKFLVPF-YWXYLSGTSA-N | BBUSRIKDKFLVPF | N/A | N/A | N/A |
| 3-Indolepropionic acid | 190.086 | 190.086 | M+H | 0 | 0 | 13.4428 | 806.568 | 4 | qTof | C1=CC=C2C(=C1)C(=CN2)CCC(=O)O |  |  | Bronze | 979 | 0.79184 | 63951.3 | GOLXRNDWAUTYKT-UHFFFAOYSA-N | GOLXRNDWAUTYKT | Organoheterocyclic compounds | Indoles and derivatives | Indolyl carboxylic acids and derivatives |
| "(3R)-3-((3R,5S,7R,8R,9S,10S,13R,17R)-3,7-dihydroxy-10,13-dimethylhexadecahydro-1H-cyclopenta[a]phenanthren-17-yl)butanoic acid" | 361.273 | 361.275 | M-H2O+H | 0.0019836 | 5.4907 | 15.5117 | 930.702 | 11 | qTof | C[C@@H]([C@H]1CC[C@]2([H])[C@]1(C)CC[C@@]3([H])[C@@]2([H])[C@H](O)C[C@@]4([H])[C@]3(C)CC[C@@H](O)C4)CC(O)=O | "InChI=1S/C23H38O4/c1-13(10-20(26)27)16-4-5-17-21-18(7-9-23(16,17)3)22(2)8-6-15(24)11-14(22)12-19(21)25/h13-19,21,24-25H,4-12H2,1-3H3,(H,26,27)/t13-,14+,15-,16-,17+,18+,19-,21+,22+,23-/m1/s1" | N/A | Gold | 16293 | 0.736815 | 2702.66 | QYYDXDSPYPOWRO-AYTZMJRQSA-N | QYYDXDSPYPOWRO | Lipids and lipid-like molecules | Steroids and steroid derivatives | Bile acids, alcohols and derivatives |
| "(3S,8S,9S,10R,13R,14S,17R)-17-((2R)-7-hydroxy-6-methylheptan-2-yl)-10,13-dimethyl-2,3,4,7,8,9,10,11,12,13,14,15,16,17-tetradecahydro-1H-cyclopenta[a]phenanthren-3-ol" | 367.336 | 367.336 | M-2H2O+H | 0 | 0 | 23.8643333 | 1431.86 | 8 | qTof | C[C@@H]([C@H]1CC[C@]2([H])[C@]1(C)CC[C@@]3([H])[C@@]2([H])CC=C4[C@]3(C)CC[C@H](O)C4)CCCC(C)CO | "InChI=1S/C27H46O2/c1-18(17-28)6-5-7-19(2)23-10-11-24-22-9-8-20-16-21(29)12-14-26(20,3)25(22)13-15-27(23,24)4/h8,18-19,21-25,28-29H,5-7,9-17H2,1-4H3/t18?,19-,21+,22+,23-,24+,25+,26+,27-/m1/s1" | N/A | Gold | 16692 | 0.688918 | 11807.5 | FYHRJWMENCALJY-CCDZVGGQSA-N | FYHRJWMENCALJY | Lipids and lipid-like molecules | Steroids and steroid derivatives | Bile acids, alcohols and derivatives |
| "(4R)-4-((1R,3S,5S,7R,9S,10S,13R,14S,17R)-1,3,7-trihydroxy-10,13-dimethylhexadecahydro-1H-cyclopenta[a]phenanthren-17-yl)pentanoic acid" | 391.284 | 391.284 | M-H2O+H | 0 | 0 | 16.0883167 | 965.299 | 11 | qTof | [H][C@@]1([C@H](O)C[C@H]2[C@]3(C)[C@H](O)C[C@@H](O)C2)[C@@H]3CC[C@@]4(C)[C@H]1CC[C@@H]4[C@@H](CCC(O)=O)C | "InChI=1S/C24H40O5/c1-13(4-7-21(28)29)16-5-6-17-22-18(8-9-23(16,17)2)24(3)14(11-19(22)26)10-15(25)12-20(24)27/h13-20,22,25-27H,4-12H2,1-3H3,(H,28,29)/t13-,14+,15+,16-,17+,18+,19-,20-,22+,23-,24+/m1/s1" | N/A | Gold | 17907 | 0.759001 | 18946.6 | GYUVAHWOVINGNE-RWXZXXAWSA-N | GYUVAHWOVINGNE | Lipids and lipid-like molecules | Steroids and steroid derivatives | Bile acids, alcohols and derivatives |
| AdenineHCl | 172.133 | 172.133 | M+H | 0 | 0 | 10.2695 | 616.17 | 4 | qTof | N/A | N/A | N/A | Bronze | 570 | 0.8173 | 17552.5 | N/A | N/A | N/A | N/A | N/A |
| ADENOSINE-MONOPHOSPHATE | 348.07 | 348.07 | M+H | 0 | 0 | 0.70481667 | 42.289 | 4 | Orbitrap | NC1=NC=NC2=C1N=CN2[C@@H]1O[C@H](COP(O)(O)=O)[C@@H](O)[C@H]1O | "InChI=1S/C10H14N5O7P/c11-8-5-9(13-2-12-8)15(3-14-5)10-7(17)6(16)4(22-10)1-21-23(18,19)20/h2-4,6-7,10,16-17H,1H2,(H2,11,12,13)(H2,18,19,20)/t4-,6-,7-,10-/m1/s1" | N/A | Gold | 14922 | 0.768421 | 11090.3 | UDMBCSSLTHHNCD-KQYNXXCUSA-N | UDMBCSSLTHHNCD | Nucleosides, nucleotides, and analogues | Purine nucleotides | Purine ribonucleotides |
| ADONITOL | 170.102 | 170.117 | [M+NH4]+ | 0.0149994 | 88.1788 | 9.6605 | 579.63 | 6 | ESI-QFT | N/A | InChI=1S/C5H12O5/c6-1-3(8)5(10)4(9)2-7/h3-10H,1-2H2 | N/A | Bronze | 541 | 0.76159 | 10139.6 | HEBKCHPVOIAQTA-UHFFFAOYSA-N | HEBKCHPVOIAQTA | Organic oxygen compounds | Organooxygen compounds | Carbohydrates and carbohydrate conjugates |
| Arginine | 174.2 | 175.118 | M+H | 0.00100708 | 5.75083 | 0.42676667 | 25.606 | 5 | Q-Exactive Plus | N/A | "InChI=1S/C6H14N4O2/c7-4(5(11)12)2-1-3-10-6(8)9/h4H,1-3,7H2,(H,11,12)(H4,8,9,10)/t4-/m0/s1" | ODKSFYDXXFIFQN-BYPYZUCNSA-N | Gold | 625 | 0.82455 | 17687.2 | N/A | N/A | N/A | N/A | N/A |
| Benzylpenicillin_20eV | 335.107 | 335.107 | M+H | 0.00030518 | 0.910682 | 12.0863167 | 725.179 | 11 | qTof | [H][C@]12SC(C)([C@H](C(O)=O)N1C([C@H]2NC(Cc3ccccc3)=O)=O)C | N/A | N/A | Bronze | 13427 | 0.781002 | 10719.3 | JGSARLDLIJGVTE-MBNYWOFBSA-N | JGSARLDLIJGVTE | Organic acids and derivatives | Carboxylic acids and derivatives | Amino acids, peptides, and analogues |
| C17-Sphinganine | 288.291 | 288.29 | M+H | 0.00097656 | 3.38742 | 15.95865 | 957.519 | 4 | qTof | CCCCCCCCCCCCCCC(C(CO)N)O |  |  | Bronze | 7782 | 0.68582 | 67576.2 | KFQUQCFJDMSIJF-UHFFFAOYSA-N | KFQUQCFJDMSIJF | Organic nitrogen compounds | Organonitrogen compounds | Amines |
| Ceramide (18:1/16:0) | 538.52 | 538.525 | M+H | 0.0050049 | 9.29377 | 27.5705 | 1654.23 | 7 | qTof |  |  |  | Bronze | 23230 | 0.800895 | 5040.82 | N/A | N/A | N/A | N/A | N/A |
| CHOLATE | 391.284 | 391.284 | M-H2O+H | 0 | 0 | 16.0827833 | 964.967 | 8 | Orbitrap | [H][C@@]1(CC[C@@]2([H])[C@]3([H])[C@H](O)C[C@]4([H])C[C@H](O)CC[C@]4(C)[C@@]3([H])C[C@H](O)[C@]12C)[C@H](C)CCC(O)=O | InChI=1S/C24H40O5/c1-13(4-7-21(28)29)16-5-6-17-22-18(12-20(27)24(16,17)3)23(2)9-8-15(25)10-14(23)11-19(22)26/h13-20,22,25-27H,4-12H2,1-3H3,(H,28,29)/t13-,14+,15-,16-,17+,18+,19-,20+,22+,23+,24-/m1/s1 | N/A | Gold | 17910 | 0.679135 | 1083.24 | BHQCQFFYRZLCQQ-OELDTZBJSA-N | BHQCQFFYRZLCQQ | Lipids and lipid-like molecules | Steroids and steroid derivatives | Bile acids, alcohols and derivatives |
| Cholic acid | 834.609 | 834.607 | 2M+NH4 | 0.00201416 | 2.4133 | 16.0746667 | 964.48 | 15 | qTof | [H][C@@]12C[C@H](O)CC[C@]1(C)[C@@]1([H])C[C@H](O)[C@]3(C)[C@]([H])(CC[C@@]3([H])[C@]1([H])[C@H](O)C2)[C@H](C)CCC(O)=O | InChI=1S/C24H40O5/c1-13(4-7-21(28)29)16-5-6-17-22-18(12-20(27)24(16,17)3)23(2)9-8-15(25)10-14(23)11-19(22)26/h13-20,22,25-27H,4-12H2,1-3H3,(H,28,29)/t13-,14+,15-,16-,17+,18+,19-,20+,22+,23+,24-/m1/s1 | BHQCQFFYRZLCQQ-OELDTZBJSA-N | Bronze | 26502 | 0.757454 | 15951.3 | BHQCQFFYRZLCQQ-OELDTZBJSA-N | BHQCQFFYRZLCQQ | Lipids and lipid-like molecules | Steroids and steroid derivatives | Bile acids, alcohols and derivatives |
| CHOLIC ACID | 409.295 | 409.296 | [M+H]+ | 0.00097656 | 2.38596 | 14.9571 | 897.426 | 13 | ESI-QFT | N/A | InChI=1S/C24H40O5/c1-13(4-7-21(28)29)16-5-6-17-22-18(12-20(27)24(16,17)3)23(2)9-8-15(25)10-14(23)11-19(22)26/h13-20,22,25-27H,4-12H2,1-3H3,(H,28,29)/t13-,14+,15-,16-,17+,18+,19-,20+,22+,23+,24-/m1/s1 | N/A | Bronze | 18998 | 0.827296 | 30085.3 | BHQCQFFYRZLCQQ-OELDTZBJSA-N | BHQCQFFYRZLCQQ | Lipids and lipid-like molecules | Steroids and steroid derivatives | Bile acids, alcohols and derivatives |
| CORTICOSTERONE | 693.436 | 693.44 | 2M+H | 0.00402832 | 5.80922 | 15.1061167 | 906.367 | 8 | Orbitrap | CC12CCC(=O)C=C1CCC3C2C(CC4(C3CCC4C(=O)CO)C)O | InChI=1S/C21H30O4/c1-20-8-7-13(23)9-12(20)3-4-14-15-5-6-16(18(25)11-22)21(15,2)10-17(24)19(14)20/h9,14-17,19,22,24H,3-8,10-11H2,1-2H3 | N/A | Gold | 25253 | 0.761905 | 3799.67 | OMFXVFTZEKFJBZ-UHFFFAOYSA-N | OMFXVFTZEKFJBZ | Lipids and lipid-like molecules | Steroids and steroid derivatives | Hydroxysteroids |
| Cortodoxone | 347.222 | 347.222 | [M+H]+ | 0 | 0 | 14.4114167 | 864.685 | 9 | ESI-QTOF | N/A | InChI=1S/C21H30O4/c1-19-8-5-14(23)11-13(19)3-4-15-16(19)6-9-20(2)17(15)7-10-21(20,25)18(24)12-22/h11,15-17,22,25H,3-10,12H2,1-2H3/t15?,16?,17?,19?,20?,21-/m0/s1 | N/A | Bronze | 14887 | 0.621826 | 17469.8 | WHBHBVVOGNECLV-VLAJLFEZSA-N | WHBHBVVOGNECLV | Lipids and lipid-like molecules | Steroids and steroid derivatives | Hydroxysteroids |
| Decanoyl-L-carnitine | -1 | 316.248 | M+H | 0.00799561 | 25.2833 | 15.1381833 | 908.291 | 4 | qTof | CCCCCCCCCC(=O)OC(CC(=O)[O-])C[N+](C)(C)C |  |  | Bronze | 11054 | 0.720513 | 8515.22 | LZOSYCMHQXPBFU-UHFFFAOYSA-N | LZOSYCMHQXPBFU | Lipids and lipid-like molecules | Fatty Acyls | Fatty acid esters |
| D-TRYPTOPHAN | 205.1 | 205.097 | M+H | 0.003006 | 14.6562 | 2.34515 | 140.709 | 4 | Q-Exactive Plus | c1cccc2c1c(c[nH]2)C[C@@H](N)C(=O)O | N/A | N/A | Gold | 1790 | 0.849366 | 130012 | QIVBCDIJIAJPQS-SECBINFHSA-N | QIVBCDIJIAJPQS | Organoheterocyclic compounds | Indoles and derivatives | Indolyl carboxylic acids and derivatives |
| Ethylenediaminetetraacetic acid EDTA | 315.079 | 315.08 | M+Na | 0.00097656 | 3.09942 | 0.24641667 | 14.785 | 10 | qTof | C(CN(CC(=O)O)CC(=O)O)N(CC(=O)O)CC(=O)O | InChI=1S/C10H16N2O8/c13-7(14)3-11(4-8(15)16)1-2-12(5-9(17)18)6-10(19)20/h1-6H2,(H,13,14)(H,15,16)(H,17,18)(H,19,20) |  | Bronze | 10864 | 0.858343 | 25365.7 | KCXVZYZYPLLWCC-UHFFFAOYSA-N | KCXVZYZYPLLWCC | Organic acids and derivatives | Carboxylic acids and derivatives | Tetracarboxylic acids and derivatives |
| Glutamic Acid | 148.06 | 148.061 | M+H | 0.00061035 | 4.12231 | 0.53613333 | 32.168 | 5 | qTof | C(CC(=O)O)C(C(=O)O)N | 1S/C5H9NO4/c6-3(5(9)10)1-2-4(7)8/h3H,1-2,6H2,(H,7,8)(H,9,10)/t3-/m0/s1 |  | Bronze | 233 | 0.802986 | 35877.7 | WHUUTDBJXJRKMK-UHFFFAOYSA-N | WHUUTDBJXJRKMK | Organic acids and derivatives | Carboxylic acids and derivatives | Amino acids, peptides, and analogues |
| glycochenodeoxycholic acid | 450.321 | 450.321 | M+H | 0 | 0 | 16.4355167 | 986.131 | 13 | Orbitrap | C[C@H](CCC(=O)NCC(=O)O)[C@H]1CC[C@@H]2[C@@]1(CC[C@H]3[C@H]2[C@@H](C[C@H]4[C@@]3(CC[C@H](C4)O)C)O)C | 1S/C26H43NO5/c1-15(4-7-22(30)27-14-23(31)32)18-5-6-19-24-20(9-11-26(18,19)3)25(2)10-8-17(28)12-16(25)13-21(24)29/h15-21,24,28-29H,4-14H2,1-3H3,(H,27,30)(H,31,32)/t15?,16-,17+,18+,19-,20-,21+,24-,25?,26?/m0/s1 | N/A | Gold | 20672 | 0.843696 | 13418.6 | GHCZAUBVMUEKKP-GYPHWSFCSA-N | GHCZAUBVMUEKKP | Lipids and lipid-like molecules | Steroids and steroid derivatives | Bile acids, alcohols and derivatives |
| GLYCOCHOLATE | 430.295 | 430.296 | M-2H2O+H | 0.00097656 | 2.26952 | 14.9849333 | 899.096 | 8 | Orbitrap | [H][C@@]12CC[C@H]([C@H](C)CCC(=O)NCC(O)=O)[C@@]1(C)[C@@H](O)C[C@@]1([H])[C@@]2([H])[C@H](O)CC2C[C@H](O)CC[C@]12C | "InChI=1S/C26H43NO6/c1-14(4-7-22(31)27-13-23(32)33)17-5-6-18-24-19(12-21(30)26(17,18)3)25(2)9-8-16(28)10-15(25)11-20(24)29/h14-21,24,28-30H,4-13H2,1-3H3,(H,27,31)(H,32,33)/t14-,15?,16-,17-,18+,19+,20-,21+,24+,25+,26-/m1/s1" | N/A | Gold | 19929 | 0.67103 | 2753.71 | RFDAIACWWDREDC-MZMBZMQMSA-N | RFDAIACWWDREDC | Lipids and lipid-like molecules | Steroids and steroid derivatives | Bile acids, alcohols and derivatives |
| Glycocholic Acid | 488.298 | 488.297 | [M+Na]+ | 0.00100708 | 2.06243 | 14.9632667 | 897.796 | 5 | ESI-QFT | N/A | InChI=1S/C26H43NO6/c1-14(4-7-22(31)27-13-23(32)33)17-5-6-18-24-19(12-21(30)26(17,18)3)25(2)9-8-16(28)10-15(25)11-20(24)29/h14-21,24,28-30H,4-13H2,1-3H3,(H,27,31)(H,32,33)/t14-,15+,16-,17-,18+,19+,20-,21+,24+,25+,26-/m1/s1 | N/A | Bronze | 22141 | 0.658162 | 2593.87 | RFDAIACWWDREDC-FRVQLJSFSA-N | RFDAIACWWDREDC | Lipids and lipid-like molecules | Steroids and steroid derivatives | Bile acids, alcohols and derivatives |
| glycoursodeoxycholic acid | 414.3 | 414.3 | M-2H2O+H | 0 | 0 | 16.4296833 | 985.781 | 9 | Orbitrap | C[C@H](CCC(=O)NCC(=O)O)[C@H]1CC[C@@H]2[C@@]1(CC[C@H]3[C@H]2[C@H](C[C@H]4[C@@]3(CC[C@H](C4)O)C)O)C | 1S/C26H43NO5/c1-15(4-7-22(30)27-14-23(31)32)18-5-6-19-24-20(9-11-26(18,19)3)25(2)10-8-17(28)12-16(25)13-21(24)29/h15-21,24,28-29H,4-14H2,1-3H3,(H,27,30)(H,31,32)/t15?,16-,17+,18+,19-,20-,21-,24-,25?,26?/m0/s1 | N/A | Gold | 19238 | 0.727954 | 953.316 | GHCZAUBVMUEKKP-XROMFQGDSA-N | GHCZAUBVMUEKKP | Lipids and lipid-like molecules | Steroids and steroid derivatives | Bile acids, alcohols and derivatives |
| hyodeoxycholic acid | 375.29 | 375.288 | M-H2O+H | 0.00201416 | 5.36694 | 16.0874833 | 965.249 | 11 | Orbitrap | C[C@H](CCC(=O)O)[C@H]1CC[C@@H]2[C@@]1(CC[C@H]3[C@H]2C[C@@H]([C@H]4[C@@]3(CC[C@H](C4)O)C)O)C | 1S/C24H40O4/c1-14(4-7-22(27)28)17-5-6-18-16-13-21(26)20-12-15(25)8-10-24(20,3)19(16)9-11-23(17,18)2/h14-21,25-26H,4-13H2,1-3H3,(H,27,28)/t14?,15-,16+,17-,18+,19+,20+,21+,23?,24?/m1/s1 | N/A | Gold | 17062 | 0.808197 | 15006.7 | DGABKXLVXPYZII-SIBKNCMHSA-N | DGABKXLVXPYZII | Lipids and lipid-like molecules | Steroids and steroid derivatives | Bile acids, alcohols and derivatives |
| LAUROYLCARNITINE | 344.28 | 344.279 | M+H | 0.00100708 | 2.92518 | 16.7228333 | 1003.37 | 5 | Orbitrap | CCCCCCCCCCCC(=O)O[C@H](CC([O-])=O)C[N+](C)(C)C | "InChI=1S/C19H37NO4/c1-5-6-7-8-9-10-11-12-13-14-19(23)24-17(15-18(21)22)16-20(2,3)4/h17H,5-16H2,1-4H3/t17-/m1/s1" | N/A | Gold | 14628 | 0.639552 | 6361.5 | FUJLYHJROOYKRA-QGZVFWFLSA-N | FUJLYHJROOYKRA | Lipids and lipid-like molecules | Fatty Acyls | Fatty acid esters |
| Linoleyl Carnitine | 424.34 | 424.343 | M+H | 0.00299072 | 7.04794 | 19.0698333 | 1144.19 | 9 | qTof | CCCCCC=CCC=CCCCCCCCC(=O)OC(CC(=O)[O-])C[N+](C)(C)C |  |  | Bronze | 19607 | 0.61318 | 10848.8 | MJLXQSQYKZWZCB-UHFFFAOYSA-N | MJLXQSQYKZWZCB | Lipids and lipid-like molecules | Fatty Acyls | Fatty acid esters |
| L-THYROXINE | 777.698 | 777.693 | [M+H] | 0.00500488 | 6.43551 | 15.3759667 | 922.558 | 21 | qTof | N/A | N[C@@H](Cc1cc(I)c(Oc2cc(I)c(O)c(I)c2)c(I)c1)C(=O)O | N/A | Gold | 26013 | 0.73991 | 1792.1 | N/A | N/A | N/A | N/A | N/A |
| mannitol | 183.08 | 183.088 | M+H | 0.00799561 | 43.6727 | 10.5058333 | 630.35 | 5 | Hybrid FT |  |  |  | Bronze | 730 | 0.793098 | 132911 | N/A | N/A | N/A | N/A | N/A |
| Massbank:AU249206 4-Hydroxyquinoline\|1H-quinolin-4-one | 146.06 | 146.06 | M+H | 0 | 0 | 2.43288333 | 145.973 | 4 | qTof | OC1=CC=NC2=CC=CC=C12 | 1S/C9H7NO/c11-9-5-6-10-8-4-2-1-3-7(8)9/h1-6H,(H,10,11) | N/A | Bronze | 222 | 0.796564 | 91654.8 | PMZDQRJGMBOQBF-UHFFFAOYSA-N | PMZDQRJGMBOQBF | Organoheterocyclic compounds | Quinolines and derivatives | Quinolones and derivatives |
| Massbank:AU252106 4-Hydroxy-1-(2-hydroxyethyl)-2,2,6,6-tetramethylpiperidine\|1-Piperidineethanol, 4-hydroxy-2,2,6,6-tetramethyl-\|1-(2-hydroxyethyl)-2,2,6,6-tetramethylpiperidin-4-ol | 202.18 | 202.181 | M+H | 0.00100708 | 4.98111 | 1.06686667 | 64.012 | 5 | qTof | CC1(C)CC(O)CC(C)(C)N1CCO | 1S/C11H23NO2/c1-10(2)7-9(14)8-11(3,4)12(10)5-6-13/h9,13-14H,5-8H2,1-4H3 | N/A | Bronze | 1415 | 0.75341 | 4295.74 | STEYNUVPFMIUOY-UHFFFAOYSA-N | STEYNUVPFMIUOY | N/A | N/A | N/A |
| Massbank:AU406506 3,6,9,12-Tetraoxatetracosan-1-ol\|Tetraethylene glycol monododecyl ether\|2-[2-[2-(2-dodecoxyethoxy)ethoxy]ethoxy]ethanol-Contaminant | 363.31 | 363.311 | M+H | 0.00100708 | 2.77196 | 21.2686667 | 1276.12 | 7 | qTof | CCCCCCCCCCCCOCCOCCOCCOCCO | 1S/C20H42O5/c1-2-3-4-5-6-7-8-9-10-11-13-22-15-17-24-19-20-25-18-16-23-14-12-21/h21H,2-20H2,1H3 | N/A | Bronze | 16400 | 0.719529 | 3011.09 | WPMWEFXCIYCJSA-UHFFFAOYSA-N | WPMWEFXCIYCJSA | Organic oxygen compounds | Organooxygen compounds | Ethers |
| Massbank:BML00646 Jasmonic acid | 211.133 | 211.132 | [M+H]+ | 0.00099182 | 4.69761 | 14.33045 | 859.827 | 4 | LC-ESI-QTOF | CCC=CCC1C(CCC1=O)CC(=O)O | 1S/C12H18O3/c1-2-3-4-5-10-9(8-12(14)15)6-7-11(10)13/h3-4,9-10H,2,5-8H2,1H3,(H,14,15) | N/A | Bronze | 2167 | 0.686314 | 10527.4 | ZNJFBWYDHIGLCU-UHFFFAOYSA-N | ZNJFBWYDHIGLCU | Lipids and lipid-like molecules | Fatty Acyls | Lineolic acids and derivatives |
| Massbank:CE000170 Quercetin | 303.05 | 303.051 | [M+H]+ | 0.00100708 | 3.32315 | 11.2451833 | 674.711 | 9 | LC-ESI-ITFT | C1=CC(=C(C=C1C2=C(C(=O)C3=C(C=C(C=C3O2)O)O)O)O)O | 1S/C15H10O7/c16-7-4-10(19)12-11(5-7)22-15(14(21)13(12)20)6-1-2-8(17)9(18)3-6/h1-5,16-19,21H | N/A | Bronze | 9751 | 0.795523 | 7357.74 | REFJWTPEDVJJIY-UHFFFAOYSA-N | REFJWTPEDVJJIY | Phenylpropanoids and polyketides | Flavonoids | Flavones |
| Massbank:EA014704 Fluazifop\| 2-[4-[5-(trifluoromethyl)pyridin-2-yl]oxyphenoxy]propanoic acid | 328.079 | 328.079 | [M+H]+ | 0 | 0 | 17.5946667 | 1055.68 | 9 | LC-ESI-ITFT | CC(C(=O)O)OC1=CC=C(C=C1)OC2=NC=C(C=C2)C(F)(F)F | 1S/C15H12F3NO4/c1-9(14(20)21)22-11-3-5-12(6-4-11)23-13-7-2-10(8-19-13)15(16,17)18/h2-9H,1H3,(H,20,21) | N/A | Bronze | 12516 | 0.863734 | 2860.09 | YUVKUEAFAVKILW-UHFFFAOYSA-N | YUVKUEAFAVKILW | Benzenoids | Benzene and substituted derivatives | 2-phenoxypropionic acids |
| Massbank:EA282603 Ketamine\|2-(2-chlorophenyl)-2-(methylamino)-1-cyclohexanone | 238.099 | 238.099 | [M+H]+ | 0 | 0 | 9.86321667 | 591.793 | 7 | LC-ESI-ITFT | CNC1(CCCCC1=O)C2=CC=CC=C2Cl | 1S/C13H16ClNO/c1-15-13(9-5-4-8-12(13)16)10-6-2-3-7-11(10)14/h2-3,6-7,15H,4-5,8-9H2,1H3 | N/A | Bronze | 3505 | 0.964798 | 3985.43 | YQEZLKZALYSWHR-UHFFFAOYSA-N | YQEZLKZALYSWHR | Benzenoids | Benzene and substituted derivatives | Halobenzenes |
| Massbank:EQ022902 Octocrylene\|[(2R)-2-ethylhexyl] 2-cyano-3,3-diphenylprop-2-enoate | 362.211 | 362.206 | M+H | 0.00500488 | 13.8176 | 24.2318333 | 1453.91 | 7 | Hybrid FT | C(\c1ccccc1)(c1ccccc1)=C(/C(OC[C@@H](CCCC)CC)=O)C#N | 1S/C24H27NO2/c1-3-5-12-19(4-2)18-27-24(26)22(17-25)23(20-13-8-6-9-14-20)21-15-10-7-11-16-21/h6-11,13-16,19H,3-5,12,18H2,1-2H3/t19-/m1/s1 | N/A | Bronze | 16320 | 0.7519 | 3043.61 | FMJSMJQBSVNSBF-LJQANCHMSA-N | FMJSMJQBSVNSBF | Benzenoids | Benzene and substituted derivatives | Diphenylmethanes |
| Massbank:EQ319802 Corticosterone\|11b,21-Dihydroxyprogesterone\|(8S,9S,10R,11S,13S,14S,17S)-11-hydroxy-17-(2-hydroxyacetyl)-10,13-dimethyl-1,2,6,7,8,9,11,12,14,15,16,17-dodecahydrocyclopenta[a]phenanthren-3-one | 347.222 | 347.222 | M+H | 0 | 0 | 15.0716667 | 904.3 | 13 | Hybrid FT | O=C4\C=C2/[C@]([C@H]1[C@@H](O)C[C@@]3([C@@H](C(=O)CO)CC[C@H]3[C@@H]1CC2)C)(C)CC4 | 1S/C21H30O4/c1-20-8-7-13(23)9-12(20)3-4-14-15-5-6-16(18(25)11-22)21(15,2)10-17(24)19(14)20/h9,14-17,19,22,24H,3-8,10-11H2,1-2H3/t14-,15-,16+,17-,19+,20-,21-/m0/s1 | N/A | Bronze | 14885 | 0.907732 | 35331.9 | OMFXVFTZEKFJBZ-HJTSIMOOSA-N | OMFXVFTZEKFJBZ | Lipids and lipid-like molecules | Steroids and steroid derivatives | Hydroxysteroids |
| MassbankEU:SM876702 Lauryl diethanolamide\|N,N-bis(2-hydroxyethyl)dodecanamide | 288.253 | 288.254 | [M+H]+ | 0.00100708 | 3.49374 | 17.8596667 | 1071.58 | 5 | LC-ESI-QFT | CCCCCCCCCCCC(=O)N(CCO)CCO | 1S/C16H33NO3/c1-2-3-4-5-6-7-8-9-10-11-16(20)17(12-14-18)13-15-19/h18-19H,2-15H2,1H3 | N/A | Bronze | 7778 | 0.818915 | 18417.7 | AOMUHOFOVNGZAN-UHFFFAOYSA-N | AOMUHOFOVNGZAN | Lipids and lipid-like molecules | Fatty Acyls | Fatty amides |
| MassbankEU:SM876801 N,N-Dimethyldodecylamine N-oxide\|Lauramine oxide\|N,N-dimethyldodecan-1-amine oxide | 230.248 | 230.248 | [M+H]+ | 0 | 0 | 16.7585 | 1005.51 | 4 | LC-ESI-QFT | CCCCCCCCCCCC[N+](C)(C)[O-] | 1S/C14H31NO/c1-4-5-6-7-8-9-10-11-12-13-14-15(2,3)16/h4-14H2,1-3H3 | N/A | Bronze | 3150 | 0.60072 | 5534.8 | SYELZBGXAIXKHU-UHFFFAOYSA-N | SYELZBGXAIXKHU | Organic nitrogen compounds | Organonitrogen compounds | Aminoxides |
| MassbankEU:SM878901 N-Butylbenzenesulfonamide | 214.09 | 214.09 | [M+H]+ | 0 | 0 | 2.25256667 | 135.154 | 5 | LC-ESI-QFT | CCCCNS(=O)(=O)C1=CC=CC=C1 | 1S/C10H15NO2S/c1-2-3-9-11-14(12,13)10-7-5-4-6-8-10/h4-8,11H,2-3,9H2,1H3 | N/A | Bronze | 2313 | 0.771259 | 23417.6 | IPRJXAGUEGOFGG-UHFFFAOYSA-N | IPRJXAGUEGOFGG | Benzenoids | Benzene and substituted derivatives | Benzenesulfonamides |
| Massbank: Folic_Acid | 442.148 | 442.147 | [M+H]+ | 0.0010071 | 2.2777 | 8.5163 | 510.978 | 4 | LC-ESI-QTOF | [H]OC(=O)C([H])([H])C([H])([H])C([H])(C(=O)O[H])N([H])C(=O)c(c([H])3)c([H])c([H])c(c([H])3)N([H])C([H])([H])c(c([H])2)nc(C(=O)1)c(n2)N([H])C(N([H])[H])=N1 | 1S/C19H19N7O6/c20-19-25-15-14(17(30)26-19)23-11(8-22-15)7-21-10-3-1-9(2-4-10)16(29)24-12(18(31)32)5-6-13(27)28/h1-4,8,12,21H,5-7H2,(H,24,29)(H,27,28)(H,31,32)(H3,20,22,25,26,30) | N/A | Bronze | 20500 | 0.950337 | 12094.3 | OVBPIULPVIDEAO-UHFFFAOYSA-N | OVBPIULPVIDEAO | Organoheterocyclic compounds | Pteridines and derivatives | Pterins and derivatives |
| Massbank: Genistein\|5,7-dihydroxy-3-(4-hydroxyphenyl)chromen-4-one | 271.06 | 271.06 | M+H | 0 | 0 | 14.4712333 | 868.274 | 5 | Hybrid FT | OC1=CC=C(C=C1)C1=COC2=CC(O)=CC(O)=C2C1=O | 1S/C15H10O5/c16-9-3-1-8(2-4-9)11-7-20-13-6-10(17)5-12(18)14(13)15(11)19/h1-7,16-18H | N/A | Bronze | 6126 | 0.620357 | 994.572 | TZBJGXHYKVUXJN-UHFFFAOYSA-N | TZBJGXHYKVUXJN | Phenylpropanoids and polyketides | Isoflavonoids | Isoflav-2-enes |
| Massbank:KNA00309 Inosine | 137.05 | 137.052 | [M+H] | 0.001999 | 14.5852 | 18.768 | 1126.08 | 4 | LC-ESI-ITFT | OC[C@@H](O1)[C@@H](O)[C@@H](O)[C@@H]1n(c3)c(N=2)c(n3)C(=O)NC2 | 1S/C10H12N4O5/c15-1-4-6(16)7(17)10(19-4)14-3-13-5-8(14)11-2-12-9(5)18/h2-4,6-7,10,15-17H,1H2,(H,11,12,18)/t4-,6-,7-,10-/m1/s1 | N/A | Bronze | 170 | 0.662728 | 9014.06 | UGQMRVRMYYASKQ-KQYNXXCUSA-N | UGQMRVRMYYASKQ | N/A | N/A | N/A |
| Massbank:KW100702 1,3-diphenylguanidine | 212.118 | 212.118 | M+H | 0 | 0 | 9.96701667 | 598.021 | 4 | Hybrid FT | N=C(Nc1ccccc1)Nc2ccccc2 | 1S/C13H13N3/c14-13(15-11-7-3-1-4-8-11)16-12-9-5-2-6-10-12/h1-10H,(H3,14,15,16) | N/A | Bronze | 2251 | 0.965188 | 24689.9 | OWRCNXZUPFZXOS-UHFFFAOYSA-N | OWRCNXZUPFZXOS | N/A | N/A | N/A |
| Massbank: Lauramidopropyl betaine\|3-(Dodecanoylamino)propyl(carboxymethyl)dimethylammonium\|carboxymethyl-[3-(dodecanoylamino)propyl]-dimethylazanium | 343.296 | 343.297 | M | 0.00100708 | 2.93356 | 16.1161167 | 966.967 | 5 | Hybrid FT | CCCCCCCCCCCC(=O)NCCC[N+](C)(C)CC(O)=O | 1S/C19H38N2O3/c1-4-5-6-7-8-9-10-11-12-14-18(22)20-15-13-16-21(2,3)17-19(23)24/h4-17H2,1-3H3,(H-,20,22,23,24)/p+1 | N/A | Bronze | 14545 | 0.912693 | 24159.7 | MRUAUOIMASANKQ-UHFFFAOYSA-O | MRUAUOIMASANKQ | Organic acids and derivatives | Carboxylic acids and derivatives | Amino acids, peptides, and analogues |
| Massbank:LU030302 2-[4-(Diethylamino)-2-hydroxybenzoyl]benzoic acid\|2-(4-(Diethylamino)-2-hydroxybenzoyl)benzoic acid | 314.139 | 314.138 | M+H | 0.0010071 | 3.20584 | 18.1085 | 1086.51 | 9 | Hybrid FT | CCN(CC)C1=CC(O)=C(C=C1)C(=O)C1=C(C=CC=C1)C(O)=O | 1S/C18H19NO4/c1-3-19(4-2)12-9-10-15(16(20)11-12)17(21)13-7-5-6-8-14(13)18(22)23/h5-11,20H,3-4H2,1-2H3,(H,22,23) | N/A | Bronze | 10808 | 0.824922 | 8190.74 | FQNKTJPBXAZUGC-UHFFFAOYSA-N | FQNKTJPBXAZUGC | Benzenoids | Benzene and substituted derivatives | Benzophenones |
| Massbank:LU030802 Lauryldiethanolamine\|2-[dodecyl(2-hydroxyethyl)amino]ethanol | 274.274 | 274.275 | M+H | 0.0010071 | 3.6718 | 15.8762 | 952.572 | 7 | Hybrid FT | CCCCCCCCCCCCN(CCO)CCO | 1S/C16H35NO2/c1-2-3-4-5-6-7-8-9-10-11-12-17(13-15-18)14-16-19/h18-19H,2-16H2,1H3 | N/A | Bronze | 6743 | 0.860238 | 13728.5 | NKFNBVMJTSYZDV-UHFFFAOYSA-N | NKFNBVMJTSYZDV | Organic nitrogen compounds | Organonitrogen compounds | Amines |
| Massbank:LU052302 Acetyl tributyl citrate\|tributyl 2-acetyloxypropane-1,2,3-tricarboxylate | 403.233 | 403.233 | M+H | 0 | 0 | 12.6797 | 760.782 | 7 | Hybrid FT | CCCCOC(=O)CC(CC(=O)OCCCC)(OC(C)=O)C(=O)OCCCC | 1S/C20H34O8/c1-5-8-11-25-17(22)14-20(28-16(4)21,19(24)27-13-10-7-3)15-18(23)26-12-9-6-2/h5-15H2,1-4H3 | N/A | Bronze | 18661 | 0.73638 | 8358.9 | QZCLKYGREBVARF-UHFFFAOYSA-N | QZCLKYGREBVARF | Organic acids and derivatives | Carboxylic acids and derivatives | Tetracarboxylic acids and derivatives |
| Massbank:LU084702 Triethylene glycol bis(2-ethylhexanoate)\|2-[2-[2-(2-ethylhexanoyloxy)ethoxy]ethoxy]ethyl 2-ethylhexanoate-Contaminant | 403.305 | 403.306 | M+H | 0.0010071 | 2.49707 | 23.5145 | 1410.87 | 5 | Hybrid FT | CCCCC(CC)C(=O)OCCOCCOCCOC(=O)C(CC)CCCC | 1S/C22H42O6/c1-5-9-11-19(7-3)21(23)27-17-15-25-13-14-26-16-18-28-22(24)20(8-4)12-10-6-2/h19-20H,5-18H2,1-4H3 | N/A | Bronze | 18671 | 0.931836 | 21661.7 | FRQDZJMEHSJOPU-UHFFFAOYSA-N | FRQDZJMEHSJOPU | Lipids and lipid-like molecules | Fatty Acyls | Fatty acid esters |
| Massbank:LU087001 Pantothenate\|Pantothenic acid\|3-[[(2R)-2,4-dihydroxy-3,3-dimethylbutanoyl]amino]propanoic acid | 220.118 | 220.118 | M+H | 0 | 0 | 0.70368333 | 42.221 | 6 | Hybrid FT | CC(C)(CO)[C@@H](O)C(=O)NCCC(O)=O | 1S/C9H17NO5/c1-9(2,5-11)7(14)8(15)10-4-3-6(12)13/h7,11,14H,3-5H2,1-2H3,(H,10,15)(H,12,13)/t7-/m0/s1 | N/A | Bronze | 2686 | 0.736147 | 63118.8 | GHOKWGTUZJEAQD-ZETCQYMHSA-N | GHOKWGTUZJEAQD | Organic oxygen compounds | Organooxygen compounds | Alcohols and polyols |
| Massbank:NA002716 (+)-Costunolide\|Costunolide\|(3aS,6E,10E,11aR)-6,10-dimethyl-3-methylidene-3a,4,5,8,9,11a-hexahydrocyclodeca[b]furan-2-one | 233.154 | 233.153 | M+H | 0.00100708 | 4.31938 | 15.6076833 | 936.461 | 5 | Hybrid FT | C/C/1=C\CC/C(=C/[C@@H]2[C@@H](CC1)C(=C)C(=O)O2)/C | 1S/C15H20O2/c1-10-5-4-6-11(2)9-14-13(8-7-10)12(3)15(16)17-14/h5,9,13-14H,3-4,6-8H2,1-2H3/b10-5+,11-9+/t13-,14+/m0/s1 | N/A | Bronze | 3318 | 0.621974 | 13718.3 | HRYLQFBHBWLLLL-AHNJNIBGSA-N | HRYLQFBHBWLLLL | Lipids and lipid-like molecules | Prenol lipids | Terpene lactones |
| Massbank:NA002994 [6]-Gingerol\|5-hydroxy-1-(4-hydroxy-3-methoxyphenyl)decan-3-one | 295.19 | 295.19 | M+H | 0 | 0 | 17.9036667 | 1074.22 | 8 | Hybrid FT | CCCCCC(O)CC(=O)CCC1=CC(OC)=C(O)C=C1 | 1S/C17H26O4/c1-3-4-5-6-14(18)12-15(19)9-7-13-8-10-16(20)17(11-13)21-2/h8,10-11,14,18,20H,3-7,9,12H2,1-2H3 | N/A | Bronze | 8487 | 0.849699 | 5450.06 | NLDDIKRKFXEWBK-UHFFFAOYSA-N | NLDDIKRKFXEWBK | Benzenoids | Phenols | Methoxyphenols |
| Massbank:NA002995 [6]-Gingerol\|5-hydroxy-1-(4-hydroxy-3-methoxyphenyl)decan-3-one | 295.19 | 295.191 | M+H | 0.0010071 | 3.41163 | 17.8635 | 1071.81 | 7 | Hybrid FT | CCCCCC(O)CC(=O)CCC1=CC(OC)=C(O)C=C1 | 1S/C17H26O4/c1-3-4-5-6-14(18)12-15(19)9-7-13-8-10-16(20)17(11-13)21-2/h8,10-11,14,18,20H,3-7,9,12H2,1-2H3 | N/A | Bronze | 8494 | 0.66226 | 1477.36 | NLDDIKRKFXEWBK-UHFFFAOYSA-N | NLDDIKRKFXEWBK | Benzenoids | Phenols | Methoxyphenols |
| Massbank:PB000407 Phenylalanine\|2-amino-3-phenylpropanoic acid | 166.087 | 166.086 | [M+H]+ | 0.00100708 | 6.06357 | 0.28326667 | 16.996 | 5 | LC-ESI-QTOF | C1=CC=C(C=C1)CC(C(=O)O)N | 1S/C9H11NO2/c10-8(9(11)12)6-7-4-2-1-3-5-7/h1-5,8H,6,10H2,(H,11,12) | N/A | Bronze | 355 | 0.990488 | 98586.6 | COLNVLDHVKWLRT-UHFFFAOYSA-N | COLNVLDHVKWLRT | Organic acids and derivatives | Carboxylic acids and derivatives | Amino acids, peptides, and analogues |
| Massbank:PB000441 Methionine\|2-amino-4-methylsulfanylbutanoic acid | 150.059 | 150.058 | [M+H]+ | 0.00100708 | 6.71123 | 0.53986667 | 32.392 | 5 | LC-ESI-QTOF | CSCCC(C(=O)O)N | 1S/C5H11NO2S/c1-9-3-2-4(6)5(7)8/h4H,2-3,6H2,1H3,(H,7,8) | N/A | Bronze | 242 | 0.979095 | 27951.6 | FFEARJCKVFRZRR-UHFFFAOYSA-N | FFEARJCKVFRZRR | Organic acids and derivatives | Carboxylic acids and derivatives | Amino acids, peptides, and analogues |
| Massbank:PB000446 Cystine\|2-amino-3-(2-amino-3-hydroxy-3-oxopropyl)disulfanylpropanoic acid | 241.032 | 241.031 | [M+H]+ | 0.00099182 | 4.11489 | 0.75648333 | 45.389 | 5 | LC-ESI-QTOF | C(C(C(=O)O)N)SSCC(C(=O)O)N | 1S/C6H12N2O4S2/c7-3(5(9)10)1-13-14-2-4(8)6(11)12/h3-4H,1-2,7-8H2,(H,9,10)(H,11,12) | N/A | Bronze | 4279 | 0.65183 | 1761.17 | LEVWYRKDKASIDU-UHFFFAOYSA-N | LEVWYRKDKASIDU | Organic acids and derivatives | Carboxylic acids and derivatives | Amino acids, peptides, and analogues |
| Massbank:PR306677 Quercetin-3-O-glucuronide | 477.067 | 477.049 | M-H | 0.017975 | 37.6778 | 12.0591 | 723.546 | 5 | qTof | OC1C(O)C(OC2=C(OC3=CC(O)=CC(O)=C3C2=O)C2=CC(O)=C(O)C=C2)OC(C1O)C(O)=O | 1S/C21H18O13/c22-7-4-10(25)12-11(5-7)32-17(6-1-2-8(23)9(24)3-6)18(13(12)26)33-21-16(29)14(27)15(28)19(34-21)20(30)31/h1-5,14-16,19,21-25,27-29H,(H,30,31) | N/A | Bronze | 21777 | 0.668656 | 1832.5 | DUBCCGAQYVUYEU-UHFFFAOYSA-N | DUBCCGAQYVUYEU | Phenylpropanoids and polyketides | Flavonoids | Flavonoid glycosides |
| Massbank:PR309277 Flavone base + 3O, 2MeO, O-HexA | 505.099 | 505.097 | M-H | 0.0020142 | 3.98765 | 13.0578 | 783.468 | 5 | qTof | O=C(O)C4OC(OC1=CC(O)=C2C(=O)C=C(OC2(=C1))C=3C=C(OC)C(O)=C(OC)C=3)C(O)C(O)C4(O) | 1S/C23H22O13/c1-32-14-3-8(4-15(33-2)17(14)26)12-7-11(25)16-10(24)5-9(6-13(16)35-12)34-23-20(29)18(27)19(28)21(36-23)22(30)31/h3-7,18-21,23-24,26-29H,1-2H3,(H,30,31) | N/A | Bronze | 22566 | 0.684659 | 6294.89 | HJWFFBNADKDQPV-UHFFFAOYSA-N | HJWFFBNADKDQPV | Phenylpropanoids and polyketides | Flavonoids | Flavonoid glycosides |
| Massbank:PR310824 N-Fructosyl isoleucine | 294.156 | 294.158 | M+H | 0.0019836 | 6.74351 | 0.8889 | 53.334 | 10 | qTof | O=C(O)C(NCC1(O)(OC(CO)C(O)C1(O)))C(C)CC | 1S/C12H23NO7/c1-3-6(2)8(11(17)18)13-5-12(19)10(16)9(15)7(4-14)20-12/h6-10,13-16,19H,3-5H2,1-2H3,(H,17,18) | N/A | Bronze | 8313 | 0.836672 | 6758.51 | VYGRYVGDPYFVCA-UHFFFAOYSA-N | VYGRYVGDPYFVCA | Organic acids and derivatives | Carboxylic acids and derivatives | Amino acids, peptides, and analogues |
| Massbank:PR310826 N-Fructosyl phenylalanine | 328.139 | 328.139 | M+H | 0 | 0 | 2.0461 | 122.766 | 6 | qTof | O=C(O)C(NCC1(O)(OC(CO)C(O)C1(O)))CC2=CC=CC=C2 | 1S/C15H21NO7/c17-7-11-12(18)13(19)15(22,23-11)8-16-10(14(20)21)6-9-4-2-1-3-5-9/h1-5,10-13,16-19,22H,6-8H2,(H,20,21) | N/A | Bronze | 12524 | 0.66634 | 3720.85 | FAVRCIXPIVJIPN-UHFFFAOYSA-N | FAVRCIXPIVJIPN | Organic acids and derivatives | Carboxylic acids and derivatives | Amino acids, peptides, and analogues |
| Massbank:PR310841 LPC 18:3 | 518.326 | 518.324 | M+H | 0.0020142 | 3.88589 | 18.2715 | 1096.29 | 8 | qTof | O=C(OCC(O)COP(=O)([O-])OCC[N+](C)(C)C)CCCCC=CCC=CCC=CCCCCC | 1S/C26H48NO7P/c1-5-6-7-8-9-10-11-12-13-14-15-16-17-18-19-20-26(29)32-23-25(28)24-34-35(30,31)33-22-21-27(2,3)4/h9-10,12-13,15-16,25,28H,5-8,11,14,17-24H2,1-4H3 | N/A | Bronze | 22849 | 0.811501 | 40487.4 | MRTUWVDDQVMUCR-UHFFFAOYSA-N | MRTUWVDDQVMUCR | Lipids and lipid-like molecules | Glycerophospholipids | Glycerophosphocholines |
| Massbank:PR310844 LPC 18:2 | 520.341 | 520.339 | M+H | 0.0020142 | 3.87085 | 18.7805 | 1126.83 | 9 | qTof | O=C(OCC(O)COP(=O)([O-])OCC[N+](C)(C)C)CCCCCCCC=CCC=CCCCCC | 1S/C26H50NO7P/c1-5-6-7-8-9-10-11-12-13-14-15-16-17-18-19-20-26(29)32-23-25(28)24-34-35(30,31)33-22-21-27(2,3)4/h9-10,12-13,25,28H,5-8,11,14-24H2,1-4H3 | N/A | Bronze | 22888 | 0.866032 | 98186.9 | SPJFYYJXNPEZDW-UHFFFAOYSA-N | SPJFYYJXNPEZDW | Lipids and lipid-like molecules | Glycerophospholipids | Glycerophosphocholines |
| Massbank:PR311057 Glutamyltyrosine | 311.124 | 311.128 | M+H | 0.0039978 | 12.8495 | 0.93866667 | 56.32 | 8 | qTof | O=C(O)C(N)CCC(=O)NC(C(=O)O)CC1=CC=C(O)C=C1 | 1S/C14H18N2O6/c15-10(13(19)20)5-6-12(18)16-11(14(21)22)7-8-1-3-9(17)4-2-8/h1-4,10-11,17H,5-7,15H2,(H,16,18)(H,19,20)(H,21,22) | N/A | Bronze | 10525 | 0.746488 | 37800.6 | VVLXCWVSSLFQDS-UHFFFAOYSA-N | VVLXCWVSSLFQDS | Organic acids and derivatives | Carboxylic acids and derivatives | Amino acids, peptides, and analogues |
| Massbank:PR311142 Cyclo(leucylprolyl) | 211.144 | 211.144 | M+H | 0 | 0 | 9.96533333 | 597.92 | 6 | qTof | O=C1NC(C(=O)N2CCCC12)CC(C)C | 1S/C11H18N2O2/c1-7(2)6-8-11(15)13-5-3-4-9(13)10(14)12-8/h7-9H,3-6H2,1-2H3,(H,12,14) | N/A | Bronze | 2212 | 0.773452 | 11872 | SZJNCZMRZAUNQT-UHFFFAOYSA-N | SZJNCZMRZAUNQT | Organic acids and derivatives | Carboxylic acids and derivatives | Amino acids, peptides, and analogues |
| Massbank:RP025102 Oleoyl-L-Carnitine\|(3R)-3-[(Z)-octadec-9-enoyl]oxy-4-(trimethylazaniumyl)butanoate | 426.358 | 426.358 | M+H | 0 | 0 | 19.819 | 1189.14 | 11 | qTof | CCCCCCCC/C=C\CCCCCCCC(=O)O[C@H](CC(=O)[O-])C[N+](C)(C)C | 1S/C25H47NO4/c1-5-6-7-8-9-10-11-12-13-14-15-16-17-18-19-20-25(29)30-23(21-24(27)28)22-26(2,3)4/h12-13,23H,5-11,14-22H2,1-4H3/b13-12-/t23-/m1/s1 | N/A | Bronze | 19746 | 0.84324 | 31812.6 | IPOLTUVFXFHAHI-WHIOSMTNSA-N | IPOLTUVFXFHAHI | Lipids and lipid-like molecules | Fatty Acyls | Fatty acid esters |
| Massbank:RP025301 Octanoyl-L-Carnitine\|L-Octanoylcarnitine\|(3R)-3-octanoyloxy-4-(trimethylazaniumyl)butanoate | 288.217 | 288.218 | M+H | 0.00097656 | 3.38829 | 13.2055667 | 792.334 | 4 | qTof | CCCCCCCC(=O)O[C@H](CC(=O)[O-])C[N+](C)(C)C | 1S/C15H29NO4/c1-5-6-7-8-9-10-15(19)20-13(11-14(17)18)12-16(2,3)4/h13H,5-12H2,1-4H3/t13-/m1/s1 | N/A | Bronze | 7776 | 0.800261 | 7479.88 | CXTATJFJDMJMIY-CYBMUJFWSA-N | CXTATJFJDMJMIY | Lipids and lipid-like molecules | Fatty Acyls | Fatty acid esters |
| Massbank:RP025602 Stearoyl-L-Carnitine\|stearoylcarnitine\|3-octadecanoyloxy-4-(trimethylazaniumyl)butanoate | 428.373 | 428.373 | M+H | 0 | 0 | 20.5658333 | 1233.95 | 4 | qTof | CCCCCCCCCCCCCCCCCC(=O)OC(CC(=O)[O-])C[N+](C)(C)C | 1S/C25H49NO4/c1-5-6-7-8-9-10-11-12-13-14-15-16-17-18-19-20-25(29)30-23(21-24(27)28)22-26(2,3)4/h23H,5-22H2,1-4H3 | N/A | Bronze | 19854 | 0.759354 | 15332.1 | FNPHNLNTJNMAEE-UHFFFAOYSA-N | FNPHNLNTJNMAEE | Lipids and lipid-like molecules | Fatty Acyls | Fatty acid esters |
| Massbank:RP030803 N6-Threonylcarbamoyladenosine\|t(6)a\|(2S,3R)-2-[[9-[(2R,3R,4S,5R)-3,4-dihydroxy-5-(hydroxymethyl)oxolan-2-yl]purin-6-yl]carbamoylamino]-3-hydroxybutanoic acid | 413.142 | 413.142 | M+H | 0 | 0 | 7.4691 | 448.146 | 5 | qTof | C[C@H]([C@@H](C(=O)O)NC(=O)Nc1c2c(ncn1)n(cn2)[C@H]3[C@@H]([C@@H]([C@H](O3)CO)O)O)O | 1S/C15H20N6O8/c1-5(23)7(14(26)27)19-15(28)20-11-8-12(17-3-16-11)21(4-18-8)13-10(25)9(24)6(2-22)29-13/h3-7,9-10,13,22-25H,2H2,1H3,(H,26,27)(H2,16,17,19,20,28)/t5-,6-,7+,9-,10-,13-/m1/s1 | N/A | Bronze | 19155 | 0.78817 | 18644.6 | UNUYMBPXEFMLNW-DWVDDHQFSA-N | UNUYMBPXEFMLNW | N/A | N/A | N/A |
| Massbank: Taurocholate | 516.3 | 516.299 | [M+H]+ | 0.00097656 | 1.89146 | 14.5772667 | 874.636 | 15 | LC-ESI-QTOF | C(C([H])([H])1)([H])(O[H])C([H])([H])C(C4([H])[H])(C(C(C3([H])C4([H])O[H])([H])C(C(O[H])(C(C3([H])2)(C([H])(C(C2([H])[H])([H])[H])C(C([H])([H])[H])([H])C(C([H])([H])C(N(C(C(S(=O)(=O)O[H])([H])[H])([H])[H])[H])=O)([H])[H])C([H])([H])[H])[H])([H])[H])(C([H])([H])[H])C1([H])[H])[H] | 1S/C26H45NO7S/c1-15(4-7-23(31)27-10-11-35(32,33)34)18-5-6-19-24-20(14-22(30)26(18,19)3)25(2)9-8-17(28)12-16(25)13-21(24)29/h15-22,24,28-30H,4-14H2,1-3H3,(H,27,31)(H,32,33,34)/t15?,16?,17-,18?,19?,20?,21-,22+,24?,25?,26?/m1/s1 | N/A | Bronze | 22812 | 0.907984 | 30077.5 | WBWWGRHZICKQGZ-UHFFFAOYSA-N | WBWWGRHZICKQGZ | Lipids and lipid-like molecules | Steroids and steroid derivatives | Bile acids, alcohols and derivatives |
| Massbank:TUE00282 Denatonium\|N-Benzyl-2-[(2,6-dimethylphenyl)amino]-N,N-diethyl-2-oxoethanaminium | 325.227 | 325.228 | M | 0.00100708 | 3.09655 | 13.8839167 | 833.035 | 5 | qTof | CC[N+](CC)(Cc1ccccc1)CC(=O)Nc2c(cccc2C)C | 1S/C21H28N2O/c1-5-23(6-2,15-19-13-8-7-9-14-19)16-20(24)22-21-17(3)11-10-12-18(21)4/h7-14H,5-6,15-16H2,1-4H3/p+1 | N/A | Bronze | 12277 | 0.800162 | 6078.47 | ZFQMTVNLDNXRNQ-UHFFFAOYSA-O | ZFQMTVNLDNXRNQ | Organic acids and derivatives | Carboxylic acids and derivatives | Amino acids, peptides, and analogues |
| Massbank:TUE00311 Diethyltoluamide\|DEET | 192.138 | 192.139 | M+H | 0.0010071 | 5.24144 | 15.31435 | 918.861 | 4 | qTof | CCN(CC)C(=O)c1cccc(c1)C | 1S/C12H17NO/c1-4-13(5-2)12(14)11-8-6-7-10(3)9-11/h6-9H,4-5H2,1-3H3 | N/A | Bronze | 1052 | 0.965716 | 8231.82 | MMOXZBCLCQITDF-UHFFFAOYSA-N | MMOXZBCLCQITDF | Benzenoids | Benzene and substituted derivatives | Toluenes |
| Massbank:UF424004 Bisphenol S\|4,4`-Sulfonyldiphenol\|4-(4-Hydroxyphenyl)sulfonylphenol | 251.037 | 251.04 | M+H | 0.00299072 | 11.9135 | 12.4611833 | 747.671 | 5 | Hybrid FT | OC1=CC=C(C=C1)S(=O)(=O)C1=CC=C(O)C=C1 | 1S/C12H10O4S/c13-9-1-5-11(6-2-9)17(15,16)12-7-3-10(14)4-8-12/h1-8,13-14H | N/A | Bronze | 4763 | 0.937087 | 2000 | VPWNQTHUCYMVMZ-UHFFFAOYSA-N | VPWNQTHUCYMVMZ | Benzenoids | Benzene and substituted derivatives | Benzenesulfonyl compounds |
| "methyl (R)-4-((3R,5S,7R,8R,9S,10S,13R,14S,15S,17R)-3,7,15-trihydroxy-10,13-dimethylhexadecahydro-1H-cyclopenta[a]phenanthren-17-yl)pentanoate" | 423.31 | 423.309 | M+H | 0.00100708 | 2.37906 | 17.9848333 | 1079.09 | 10 | qTof | C[C@@H]([C@H]1C[C@H](O)[C@]2([H])[C@]1(C)CC[C@@]3([H])[C@@]2([H])[C@H](O)C[C@@]4([H])[C@]3(C)CC[C@@H](O)C4)CCC(OC)=O | "InChI=1S/C25H42O5/c1-14(5-6-21(29)30-4)18-13-20(28)23-22-17(8-10-25(18,23)3)24(2)9-7-16(26)11-15(24)12-19(22)27/h14-20,22-23,26-28H,5-13H2,1-4H3/t14-,15+,16-,17+,18-,19-,20+,22+,23+,24+,25-/m1/s1" | N/A | Gold | 19562 | 0.691623 | 1190.52 | GNGSNCPQCZJNBI-AKFBEHFKSA-N | GNGSNCPQCZJNBI | N/A | N/A | N/A |
| MoNA:790553 Linoleic acid | 281.247 | 281.248 | [M+H]+ | 0.00097656 | 3.47226 | 19.993 | 1199.58 | 9 | LC-ESI-QFT | N/A | InChI=1/C18H32O2/c1-2-3-4-5-6-7-8-9-10-11-12-13-14-15-16-17-18(19)20/h6-7,9-10H,2-5,8,11-17H2,1H3,(H,19,20)/b7-6-,10-9-/f/h19H | N/A | Bronze | 7136 | 0.797332 | 49707.9 | OYHQOLUKZRVURQ-HZJYTTRNSA-N | OYHQOLUKZRVURQ | Lipids and lipid-like molecules | Fatty Acyls | Lineolic acids and derivatives |
| N-ACETYL-D-TRYPTOPHAN | 247.11 | 247.108 | M+H | 0.0019989 | 8.08912 | 11.2078333 | 672.47 | 8 | Q-Exactive Plus | O=C([C@@H](Cc1c[nH]c2c1cccc2)NC(C)=O)O | N/A | N/A | Gold | 4629 | 0.923575 | 10313.9 | DZTHIGRZJZPRDV-GFCCVEGCSA-N | DZTHIGRZJZPRDV | Organic acids and derivatives | Carboxylic acids and derivatives | Amino acids, peptides, and analogues |
| N-ACETYLLEUCINE | 174.112 | 174.112 | M+H | 0 | 0 | 8.94005 | 536.403 | 5 | Orbitrap | CC(C)C[C@H](NC(C)=O)C(O)=O | "InChI=1S/C8H15NO3/c1-5(2)4-7(8(11)12)9-6(3)10/h5,7H,4H2,1-3H3,(H,9,10)(H,11,12)/t7-/m0/s1" | N/A | Gold | 599 | 0.79612 | 1982.12 | WXNXCEHXYPACJF-ZETCQYMHSA-N | WXNXCEHXYPACJF | Organic acids and derivatives | Carboxylic acids and derivatives | Amino acids, peptides, and analogues |
| NCGC00186665-03!2,3-dihydroxypropyl hexadecanoate | 348.311 | 348.312 | M+NH4 | 0.00100708 | 2.89132 | 22.4258333 | 1345.55 | 12 | Maxis II HD Q-TOF Bruker | CCCCCCCCCCCCCCCC(=O)OCC(O)CO | InChI=1S/C19H38O4/c1-2-3-4-5-6-7-8-9-10-11-12-13-14-15-19(22)23-17-18(21)16-20/h18,20-21H,2-17H2,1H3 | N/A | Gold | 15014 | 0.87201 | 32825.5 | QHZLMUACJMDIAE-UHFFFAOYSA-N | QHZLMUACJMDIAE | Lipids and lipid-like molecules | Glycerolipids | Monoradylglycerols |
| NCGC00347612-02_C20H30O4_17-Hydroxy-15,16-epoxykauran-18-oic acid | 317.211 | 317.212 | M-H2O+H | 0.00100708 | 3.1748 | 15.3482667 | 920.896 | 6 | Maxis II HD Q-TOF Bruker | CC12CCCC(C)(C1CCC34CC(CCC23)C5(CO)OC45)C(O)=O | InChI=1S/C20H30O4/c1-17-7-3-8-18(2,16(22)23)13(17)6-9-19-10-12(4-5-14(17)19)20(11-21)15(19)24-20/h12-15,21H,3-11H2,1-2H3,(H,22,23) | N/A | Gold | 11255 | 0.60517 | 14117 | VUKHFRDPHIDEAW-UHFFFAOYSA-N | VUKHFRDPHIDEAW | Lipids and lipid-like molecules | Prenol lipids | Diterpenoids |
| NCGC00380283-01!4-[5-[[4-[5-[acetyl(hydroxy)amino]pentylamino]-4-oxobutanoyl]-hydroxyamino]pentylamino]-4-oxobutanoic acid | 478.287 | 478.293 | M+NH4 | 0.00601196 | 12.5698 | 18.7771667 | 1126.63 | 9 | Maxis II HD Q-TOF Bruker | CC(=O)N(O)CCCCCNC(=O)CCC(=O)N(O)CCCCCNC(=O)CCC(O)=O | InChI=1S/C20H36N4O8/c1-16(25)23(31)14-6-2-4-12-21-17(26)8-10-19(28)24(32)15-7-3-5-13-22-18(27)9-11-20(29)30/h31-32H,2-15H2,1H3,(H,21,26)(H,22,27)(H,29,30) | N/A | Gold | 21858 | 0.810271 | 29706.3 | VJQANBSDAGCDJE-UHFFFAOYSA-N | VJQANBSDAGCDJE | Lipids and lipid-like molecules | Fatty Acyls | Fatty amides |
| NCGC00384828-01! | 527.275 | 527.287 | M+NH4 | 0.0119629 | 22.6881 | 15.1289333 | 907.736 | 9 | Maxis II HD Q-TOF Bruker | CC(=O)OC[C@]1(C)[C@H](CC[C@@]2(C)[C@H]1CC[C@@]3(C)OC4=C(CC23)C(=O)OC(=C4)C5=CN=CC=C5)OC(C)=O | InChI=1S/C29H35NO7/c1-17(31)34-16-28(4)23-8-11-29(5)24(27(23,3)10-9-25(28)35-18(2)32)13-20-22(37-29)14-21(36-26(20)33)19-7-6-12-30-15-19/h6-7,12,14-15,23-25H,8-11,13,16H2,1-5H3/t23-,24?,25+,27+,28+,29-/m1/s1 | N/A | Gold | 23044 | 0.709729 | 1805.56 | LNZRIIIDRGIMHV-CBDOUEBYSA-N | LNZRIIIDRGIMHV | N/A | N/A | N/A |
| NCGC00384939-01_C28H38O8_Methyl 3-acetoxy-16-hydroxy-4,4,8,12,16-pentamethyl-15,17,19-trioxoandrost-11-ene-14-carboxylate | 520.29 | 520.292 | M+NH4 | 0.0020142 | 3.87123 | 18.6745 | 1120.47 | 6 | Maxis II HD Q-TOF Bruker | COC(=O)C12C(=O)C(C)(O)C(=O)C1(C)\C(=C/C3C2(C)CCC4C(C)(C)C(CCC34C=O)OC(C)=O)C | InChI=1S/C28H38O8/c1-15-13-18-24(5,28(22(33)35-8)21(32)26(7,34)20(31)25(15,28)6)11-9-17-23(3,4)19(36-16(2)30)10-12-27(17,18)14-29/h13-14,17-19,34H,9-12H2,1-8H3 | N/A | Gold | 22884 | 0.64796 | 4999.16 | VNBOCIKTDWMWMX-UHFFFAOYSA-N | VNBOCIKTDWMWMX | Lipids and lipid-like molecules | Steroids and steroid derivatives | Steroid esters |
| NCGC00385123-01_C22H30O6_7b,9-Dihydroxy-3-(hydroxymethyl)-1,1,6,8-tetramethyl-5-oxo-1,1a,1b,4,4a,5,7a,7b,8,9-decahydro-9aH-cyclopropa[3,4]benzo[1,2-e]azulen-9a-yl acetate | 432.238 | 432.239 | M+ACN+H | 0.00100708 | 2.32992 | 17.6788333 | 1060.73 | 6 | Maxis II HD Q-TOF Bruker | CC1C(O)C2(OC(C)=O)C(C3\C=C(CO)/CC4C(\C=C(C)/C4=O)C13O)C2(C)C | InChI=1S/C22H30O6/c1-10-6-15-14(17(10)25)7-13(9-23)8-16-18-20(4,5)22(18,28-12(3)24)19(26)11(2)21(15,16)27/h6,8,11,14-16,18-19,23,26-27H,7,9H2,1-5H3 | N/A | Gold | 20056 | 0.812613 | 71126 | WZYGIALDVOKLLL-UHFFFAOYSA-N | WZYGIALDVOKLLL | Lipids and lipid-like molecules | Prenol lipids | Diterpenoids |
| NCGC00385365-01_C11H16O3_2(4H)-Benzofuranone, 5,6,7,7a-tetrahydro-6-hydroxy-4,4,7a-trimethyl-, (6S,7aR)- | 197.117 | 197.117 | M+H | 0 | 0 | 15.9359333 | 956.156 | 5 | Maxis II HD Q-TOF Bruker | CC\1(C)C[C@H](O)C[C@@]2(C)OC(=O)/C=C12 | InChI=1S/C11H16O3/c1-10(2)5-7(12)6-11(3)8(10)4-9(13)14-11/h4,7,12H,5-6H2,1-3H3/t7-,11+/m0/s1 | N/A | Gold | 1201 | 0.737676 | 843.651 | XEVQXKKKAVVSMW-WRWORJQWSA-N | XEVQXKKKAVVSMW | N/A | N/A | N/A |
| NCGC00385811-01!6-[3-[(3,4-dimethoxyphenyl)methyl]-4-methoxy-2-(methoxymethyl)butyl]-4-methoxy-1,3-benzodioxole | 415.211 | 415.212 | M-H2O+H | 0.00100708 | 2.42547 | 17.6671667 | 1060.03 | 4 | Maxis II HD Q-TOF Bruker | COCC(CC1=CC=C(OC)C(OC)=C1)C(COC)CC2=CC3=C(OCO3)C(OC)=C2 | InChI=1S/C24H32O7/c1-25-13-18(8-16-6-7-20(27-3)21(10-16)28-4)19(14-26-2)9-17-11-22(29-5)24-23(12-17)30-15-31-24/h6-7,10-12,18-19H,8-9,13-15H2,1-5H3 | N/A | Gold | 19259 | 0.852578 | 83020.5 | RCFGIEPQSDGMJJ-UHFFFAOYSA-N | RCFGIEPQSDGMJJ | N/A | N/A | N/A |
| O-ACETYLCARNITINE | 204.123 | 204.123 | M+H | 0 | 0 | 0.85366667 | 51.22 | 6 | Orbitrap | CC(=O)OC(CC([O-])=O)C[N+](C)(C)C | "InChI=1S/C9H17NO4/c1-7(11)14-8(5-9(12)13)6-10(2,3)4/h8H,5-6H2,1-4H3" | N/A | Gold | 1736 | 0.964691 | 18923.5 | RDHQFKQIGNGIED-UHFFFAOYSA-N | RDHQFKQIGNGIED | Lipids and lipid-like molecules | Fatty Acyls | Fatty acid esters |
| Oflaxacin | 362.151 | 362.152 | M+H | 0.0010071 | 2.78083 | 10.03615 | 602.169 | 9 | qTof | N/A | N/A | N/A | Bronze | 16316 | 0.981417 | 2000 | N/A | N/A | N/A | N/A | N/A |
| Oxidized Glutathione | 613.156 | 613.158 | M+H | 0.00201416 | 3.28491 | 1.03536667 | 62.122 | 23 | qTof | C(CC(=O)NC(CSSCC(C(=O)NCC(=O)O)NC(=O)CCC(C(=O)O)N)C(=O)NCC(=O)O)C(C(=O)O)N | 1S/C20H32N6O12S2/c21-9(19(35)36)1-3-13(27)25-11(17(33)23-5-15(29)30)7-39-40-8-12(18(34)24-6-16(31)32)26-14(28)4-2-10(22)20(37)38/h9-12H,1-8,21-22H2,(H,23,33)(H,24,34)(H,25,27)(H,26,28)(H,29,30)(H,31,32)(H,35,36)(H,37,38)/t9-,10-,11-,12-/m0/s1 |  | Bronze | 24401 | 0.902383 | 6359.18 | YPZRWBKMTBYPTK-UHFFFAOYSA-N | YPZRWBKMTBYPTK | Organic acids and derivatives | Carboxylic acids and derivatives | Amino acids, peptides, and analogues |
| PAF (Platelet Activating Factor) | 524.4 | 524.418 | M+H | 0.0180054 | 34.3352 | 20.5281667 | 1231.69 | 8 | qTof | CCCCCCCCCCCCCCCCOC[C@@H](OC(C)=O)COP(OCC[N+](C)(C)C)([O-])=O | InChI=1S/C26H54NO7P/c1-6-7-8-9-10-11-12-13-14-15-16-17-18-19-21-31-23-26(34-25(2)28)24-33-35(29,30)32-22-20-27(3,4)5/h26H,6-24H2,1-5H3/t26-/m1/s1 | HVAUUPRFYPCOCA-AREMUKBSSA-N | Bronze | 22977 | 0.736336 | 1999.65 | HVAUUPRFYPCOCA-AREMUKBSSA-N | HVAUUPRFYPCOCA | Lipids and lipid-like molecules | Glycerophospholipids | Glycerophosphocholines |
| Palmitoylcarnitine | 400.342 | 400.343 | [M+H]+ | 0.00097656 | 2.43932 | 19.3935 | 1163.61 | 10 | ESI-QTOF | N/A | InChI=1S/C23H45NO4/c1-5-6-7-8-9-10-11-12-13-14-15-16-17-18-23(27)28-21(19-22(25)26)20-24(2,3)4/h21H,5-20H2,1-4H3/t21-/m0/s1 | N/A | Bronze | 18485 | 0.910134 | 16045.9 | XOMRRQXKHMYMOC-NRFANRHFSA-N | XOMRRQXKHMYMOC | Lipids and lipid-like molecules | Fatty Acyls | Fatty acid esters |
| PC(0:0/18:0); [M+H]+ C26H55N1O7P1 | 523.364 | 523.348 | M+H | 0.0159912 | 30.5547 | 18.8983333 | 1133.9 | 9 | HCD; Velos | N/A | N/A | N/A | Gold | 22953 | 0.717076 | 7401.09 | N/A | N/A | N/A | N/A | N/A |
| PC(16:0/18:0); [M+H]+ C42H85N1O8P1 | 761.593 | 761.578 | M+H | 0.0150146 | 19.7148 | 27.5863333 | 1655.18 | 7 | HCD; Velos | N/A | N/A | N/A | Gold | 25875 | 0.863756 | 3920.75 | N/A | N/A | N/A | N/A | N/A |
| PC(17:0/20:4); [M+H]+ C45H83N1O8P1 | 795.578 | 795.578 | M+H | 0 | 0 | 25.0113333 | 1500.68 | 4 | HCD; Velos | N/A | N/A | N/A | Gold | 26207 | 0.700064 | 1747.57 | N/A | N/A | N/A | N/A | N/A |
| PC(O-16:0/16:1); [M+H]+ C40H81N1O7P1 | 717.567 | 717.585 | M+H | 0.0180054 | 25.0922 | 26.0251667 | 1561.51 | 6 | HCD; Velos | N/A | N/A | N/A | Gold | 25481 | 0.653071 | 1812.66 | N/A | N/A | N/A | N/A | N/A |
| PYRIDOXINE | 170.081 | 170.082 | M+H | 0.0010071 | 5.92118 | 1.0539 | 63.234 | 4 | Orbitrap | CC1=NC=C(CO)C(CO)=C1O | "InChI=1S/C8H11NO3/c1-5-8(12)7(4-11)6(3-10)2-9-5/h2,10-12H,3-4H2,1H3" | N/A | Gold | 399 | 0.986403 | 1997.21 | LXNHXLLTXMVWPM-UHFFFAOYSA-N | LXNHXLLTXMVWPM | Organoheterocyclic compounds | Pyridines and derivatives | Pyridoxines |
| "(R)-4-((1R,3S,5S,7R,8S,9S,10S,12S,13R,14S,17R)-1,3,7,12-tetrahydroxy-10,13-dimethylhexadecahydro-1H-cyclopenta[a]phenanthren-17-yl)pentanoic acid" | 407.279 | 407.279 | M-H2O+H | 0 | 0 | 15.4727 | 928.362 | 10 | qTof | C[C@@H]([C@H]1CC[C@]2([H])[C@]1(C)[C@@H](O)C[C@@]3([H])[C@@]2([H])[C@H](O)C[C@@]4([H])[C@]3(C)[C@H](O)C[C@@H](O)C4)CCC(O)=O | "InChI=1S/C24H40O6/c1-12(4-7-21(29)30)15-5-6-16-22-17(11-20(28)24(15,16)3)23(2)13(9-18(22)26)8-14(25)10-19(23)27/h12-20,22,25-28H,4-11H2,1-3H3,(H,29,30)/t12-,13+,14+,15-,16+,17+,18-,19-,20+,22+,23+,24-/m1/s1" | N/A | Gold | 18801 | 0.722364 | 18319.5 | UYVVLXVBEQAATF-WAIVXGPNSA-N | UYVVLXVBEQAATF | Lipids and lipid-like molecules | Steroids and steroid derivatives | Bile acids, alcohols and derivatives |
| "(R)-4-((3R,4R,5S,8S,9S,10R,13R,14S,17R)-3,4-dihydroxy-10,13-dimethylhexadecahydro-1H-cyclopenta[a]phenanthren-17-yl)pentanoic acid" | 393.3 | 393.299 | M+H | 0.00097656 | 2.483 | 17.8661667 | 1071.97 | 9 | qTof | C[C@@H]([C@H]1CC[C@]2([H])[C@]1(C)CC[C@@]3([H])[C@@]2([H])CC[C@@]4([H])[C@]3(C)CC[C@@H](O)[C@@H]4O)CCC(O)=O | "InChI=1S/C24H40O4/c1-14(4-9-21(26)27)16-7-8-17-15-5-6-19-22(28)20(25)11-13-24(19,3)18(15)10-12-23(16,17)2/h14-20,22,25,28H,4-13H2,1-3H3,(H,26,27)/t14-,15+,16-,17+,18+,19-,20-,22-,23-,24-/m1/s1" | N/A | Gold | 18125 | 0.726951 | 13095.2 | LKUNZSUKADSCME-MZWKDJGJSA-N | LKUNZSUKADSCME | Lipids and lipid-like molecules | Steroids and steroid derivatives | Bile acids, alcohols and derivatives |
| "((R)-4-((3R,5R,6S,7R,8S,9S,10R,13R,14S,17R)-3,6,7-trihydroxy-10,13-dimethylhexadecahydro-1H-cyclopenta[a]phenanthren-17-yl)pent-2-enoyl)glycine" | 464.301 | 464.302 | M+H | 0.00100708 | 2.16902 | 14.1834167 | 851.005 | 15 | qTof | C[C@@H]([C@H]1CC[C@]2([H])[C@]1(C)CC[C@@]3([H])[C@@]2([H])[C@@H](O)[C@@H](O)[C@@]4([H])[C@]3(C)CC[C@@H](O)C4)/C=C/C(NCC(O)=O)=O | "InChI=1S/C26H41NO6/c1-14(4-7-20(29)27-13-21(30)31)16-5-6-17-22-18(9-11-25(16,17)2)26(3)10-8-15(28)12-19(26)23(32)24(22)33/h4,7,14-19,22-24,28,32-33H,5-6,8-13H2,1-3H3,(H,27,29)(H,30,31)/b7-4+/t14-,15-,16-,17+,18+,19+,22+,23+,24-,25-,26-/m1/s1" | N/A | Gold | 21260 | 0.777504 | 1105.12 | XZDJHXFLHVJPMG-TUWKBOJTSA-N | XZDJHXFLHVJPMG | N/A | N/A | N/A |
| "(R)-4-((3R,5S,8R,9S,10S,12R,13R,14S,17R)-3,12-dihydroxy-10,13-dimethylhexadecahydro-1H-cyclopenta[a]phenanthren-17-yl)pentanoic acid" | 415.282 | 415.282 | M+Na | 0 | 0 | 21.5436667 | 1292.62 | 5 | qTof | C[C@@H]([C@H]1CC[C@]2([H])[C@]1(C)[C@H](O)C[C@@]3([H])[C@@]2([H])CC[C@]4([H])[C@]3(C)CC[C@@H](O)C4)CCC(O)=O | "InChI=1S/C24H40O4/c1-14(4-9-22(27)28)18-7-8-19-17-6-5-15-12-16(25)10-11-23(15,2)20(17)13-21(26)24(18,19)3/h14-21,25-26H,4-13H2,1-3H3,(H,27,28)/t14-,15+,16-,17+,18-,19+,20+,21-,23+,24-/m1/s1" | N/A | Gold | 19300 | 0.777554 | 8824.46 | KXGVEGMKQFWNSR-DNZDVJRKSA-N | KXGVEGMKQFWNSR | Lipids and lipid-like molecules | Steroids and steroid derivatives | Bile acids, alcohols and derivatives |
| Reserpine | 609.281 | 609.282 | [M+H]+ | 0.00097656 | 1.60281 | 15.9288 | 955.728 | 13 | ESI-QTOF | N/A | InChI=1S/C33H40N2O9/c1-38-19-7-8-20-21-9-10-35-16-18-13-27(44-32(36)17-11-25(39-2)30(41-4)26(12-17)40-3)31(42-5)28(33(37)43-6)22(18)15-24(35)29(21)34-23(20)14-19/h7-8,11-12,14,18,22,24,27-28,31,34H,9-10,13,15-16H2,1-6H3/t18-,22+,24-,27-,28+,31+/m1/s1 | N/A | Bronze | 24350 | 0.90238 | 2359.62 | QEVHRUUCFGRFIF-MDEJGZGSSA-N | QEVHRUUCFGRFIF | N/A | N/A | N/A |
| ReSpect:PM018116 sn-Glycero-3-phosphocholine | 258.11 | 258.11 | [M+H] | 0 | 0 | 0.53946667 | 32.368 | 5 | Q-TOF | N/A | N/A | N/A | Bronze | 5291 | 0.751333 | 19447.4 | N/A | N/A | N/A | N/A | N/A |
| RIBOFLAVIN | 377.15 | 377.146 | M+H | 0.003998 | 10.6 | 9.97035 | 598.221 | 10 | Q-Exactive Plus | CC1=CC2=C(C=C1C)N(C3=NC(=O)NC(=O)C3=N2)CC(C(C(CO)O)O)O | N/A | N/A | Gold | 17183 | 0.859897 | 9358.03 | AUNGANRZJHBGPY-UHFFFAOYSA-N | AUNGANRZJHBGPY | Organoheterocyclic compounds | Pteridines and derivatives | Alloxazines and isoalloxazines |
| Secalonic acid D | 639.169 | 639.171 | M+H | 0.0020142 | 3.15122 | 19.535 | 1172.1 | 24 | qTof | C[C@H]1CC(=O)C2=C(C3=C(C=CC(=C3O)C4=C(C5=C(C=C4)O[C@]6([C@@H]([C@H](CC(=O)C6=C5O)C)O)C(=O)OC)O)O[C@]2([C@@H]1O)C(=O)OC)O | N/A | N/A | Bronze | 24718 | 0.937641 | 3172.88 | NFZJAYYORNVZNI-OCHURCMPSA-N | NFZJAYYORNVZNI | Organoheterocyclic compounds | Benzopyrans | 1-benzopyrans |
| SM(d18:1/20:0); [M+H]+ C43H88N2O6P1 | 758.63 | 758.626 | M+H | 0.0040283 | 5.30999 | 27.741 | 1664.46 | 4 | HCD; Velos | N/A | N/A | N/A | Gold | 25851 | 0.935819 | 9934.07 | N/A | N/A | N/A | N/A | N/A |
| SM(d18:2/24:1); [M+H]+ C47H92N2O6P1 | 810.661 | 810.645 | M+H | 0.0159912 | 19.7261 | 25.3241667 | 1519.45 | 4 | HCD; Velos | N/A | N/A | N/A | Gold | 26342 | 0.91358 | 1990.97 | N/A | N/A | N/A | N/A | N/A |
| Spectral Match to 1-(1Z-Hexadecenyl)-sn-glycero-3-phosphocholine from NIST14 | 480.346 | 480.345 | M+H | 0.0010071 | 2.09657 | 18.7145 | 1122.87 | 7 | QqIT | N/A | N/A | N/A | Bronze | 21938 | 0.806137 | 13269.8 | N/A | N/A | N/A | N/A | N/A |
| Spectral Match to 1-(1Z-Octadecenyl)-2-(5Z,8Z,11Z,14Z-eicosatetraenoyl)-sn-glycero-3-phosphoethanolamine from NIST14 | 752.555 | 752.558 | M+H | 0.00299072 | 3.97409 | 27.6843333 | 1661.06 | 17 | HCD | N/A | N/A | N/A | Bronze | 25742 | 0.809906 | 13529.2 | N/A | N/A | N/A | N/A | N/A |
| Spectral Match to 1-(1Z-Octadecenyl)-2-(9Z-octadecenoyl)-sn-glycero-3-phosphocholine from NIST14 | 772.586 | 772.585 | M+H | 0.00097656 | 1.26402 | 24.2233333 | 1453.4 | 7 | HCD | N/A | N/A | N/A | Bronze | 25973 | 0.90236 | 24046.4 | N/A | N/A | N/A | N/A | N/A |
| Spectral Match to 1-(1Z-Octadecenyl)-sn-glycero-3-phosphocholine from NIST14 | 508.367 | 508.376 | M+H | 0.00900269 | 17.709 | 20.0038333 | 1200.23 | 7 | HCD | N/A | N/A | N/A | Bronze | 22655 | 0.896882 | 70832.1 | N/A | N/A | N/A | N/A | N/A |
| Spectral Match to 12,13-DiHOME from NIST14 | 315.252 | 315.255 | M+H | 0.0029907 | 9.48677 | 17.6905 | 1061.43 | 7 | HCD | N/A | N/A | N/A | Bronze | 10959 | 0.624145 | 9996.02 | N/A | N/A | N/A | N/A | N/A |
| Spectral Match to 12(13)-Epoxy-9Z-octadecenoic acid from NIST14 | 297.241 | 297.242 | M+H | 0.00100708 | 3.38809 | 20.7758333 | 1246.55 | 13 | HCD | N/A | N/A | N/A | Bronze | 8878 | 0.801965 | 7601.65 | N/A | N/A | N/A | N/A | N/A |
| Spectral Match to 1,2-Di-(9Z,12Z,15Z-octadecatrienoyl)-sn-glycero-3-phosphocholine from NIST14 | 778.54 | 778.54 | M+H | 0 | 0 | 26.236 | 1574.16 | 5 | HCD | N/A | N/A | N/A | Bronze | 26017 | 0.7489 | 2763.86 | N/A | N/A | N/A | N/A | N/A |
| Spectral Match to 1,2-Diarachidonoyl-sn-glycero-3-phosphocholine from NIST14 | 830.564 | 830.571 | M+H | 0.00695801 | 8.37745 | 26.8603333 | 1611.62 | 12 | HCD | N/A | N/A | N/A | Bronze | 26462 | 0.920617 | 32875.2 | N/A | N/A | N/A | N/A | N/A |
| Spectral Match to 1,2-Dilinoleoyl-sn-glycero-3-phosphocholine from NIST14 | 782.568 | 782.57 | M+H | 0.0020142 | 2.57378 | 20.327 | 1219.62 | 7 | HCD | N/A | N/A | N/A | Bronze | 26050 | 0.910381 | 62525.8 | N/A | N/A | N/A | N/A | N/A |
| Spectral Match to 1,2-Dioctanoyl PC from NIST14 | 510.321 | 510.308 | M+H | 0.0130005 | 25.4751 | 15.8794167 | 952.765 | 6 | HCD | N/A | N/A | N/A | Bronze | 22694 | 0.737521 | 5467.65 | N/A | N/A | N/A | N/A | N/A |
| Spectral Match to 1,2-Dipalmitoleoyl-sn-glycero-3-phosphocholine from NIST14 | 730.541 | 730.538 | M+H | 0.0029907 | 4.09385 | 26.5995 | 1595.97 | 8 | HCD | N/A | N/A | N/A | Bronze | 25594 | 0.95298 | 35971.2 | N/A | N/A | N/A | N/A | N/A |
| Spectral Match to 12-Oxo-5Z,8Z,10E,14Z-eicosatetraenoic acid from NIST14 | 319.226 | 319.227 | M+H | 0.00097656 | 3.05916 | 20.9006667 | 1254.04 | 7 | HCD | N/A | N/A | N/A | Bronze | 11681 | 0.657258 | 1137.89 | N/A | N/A | N/A | N/A | N/A |
| Spectral Match to 1,3-Dicyclohexylurea from NIST14 | 225.196 | 225.196 | M+H | 0 | 0 | 16.3216 | 979.293 | 5 | QqQ | N/A | N/A | N/A | Bronze | 2892 | 0.89555 | 28340.2 | N/A | N/A | N/A | N/A | N/A |
| Spectral Match to 13-Docosenamide, (Z)- from NIST14 | 675.675 | 675.676 | 2M+H | 0.0010376 | 1.53565 | 25.1043333 | 1506.26 | 14 | QqQ | N/A | N/A | N/A | Bronze | 25117 | 0.92125 | 54271.1 | N/A | N/A | N/A | N/A | N/A |
| Spectral Match to 13-Keto-9Z,11E-octadecadienoic acid from NIST14 | 295.226 | 295.228 | M+H | 0.0019836 | 6.71906 | 14.71475 | 882.885 | 11 | HCD | N/A | N/A | N/A | Bronze | 8522 | 0.849185 | 19860.6 | N/A | N/A | N/A | N/A | N/A |
| Spectral Match to 13S-Hydroxy-9Z,11E,15Z-octadecatrienoic acid from NIST14 | 277.216 | 277.216 | M+H-H2O | 0 | 0 | 14.71935 | 883.161 | 7 | IT/ion trap | N/A | N/A | N/A | Bronze | 6971 | 0.643005 | 884.753 | N/A | N/A | N/A | N/A | N/A |
| Spectral Match to 17.alpha.-Hydroxyprogesterone from NIST14 | 331.226 | 331.228 | M+H | 0.0019836 | 5.98879 | 16.9815 | 1018.89 | 10 | Q-TOF | N/A | N/A | N/A | Bronze | 12916 | 0.837756 | 3796.76 | N/A | N/A | N/A | N/A | N/A |
| Spectral Match to 1-(9Z-Octadecenoyl)-2-tetradecanoyl-sn-glycero-3-phosphocholine from NIST14 | 732.554 | 732.553 | M+H | 0.0010376 | 1.41641 | 21.9851667 | 1319.11 | 10 | HCD | N/A | N/A | N/A | Bronze | 25614 | 0.915858 | 38168 | N/A | N/A | N/A | N/A | N/A |
| Spectral Match to 1-(9Z-Octadecenoyl)-sn-glycero-3-phosphocholine from NIST14 | 522.354 | 522.356 | M+H | 0.00201416 | 3.85593 | 19.4851667 | 1169.11 | 8 | qTof | N/ACCCCCCCC/C=C\CCCCCCCC(=O)OC[C@H](COP(=O)([O-])OCC[NH3+])OC(=O)CCCCCCC/C=C\CCCCCCCC | InChI=1S/C41H78NO8P/c1-3-5-7-9-11-13-15-17-19-21-23-25-27-29-31-33-40(43)47-37-39(38-49-51(45,46)48-36-35-42)50-41(44)34-32-30-28-26-24-22-20-18-16-14-12-10-8-6-4-2/h17-20,39H,3-16,21-38,42H2,1-2H3,(H,45,46)/b19-17-,20-18-/t39-/m1/s1 | N/A | Bronze | 22922 | 0.93296 | 86104.6 | N/A | N/A | N/A | N/A | N/A |
| Spectral Match to 1-(9Z-Octadecenoyl)-sn-glycero-3-phosphoethanolamine from NIST14 | 480.308 | 480.31 | M+H | 0.00198364 | 4.12994 | 19.5236667 | 1171.42 | 12 | QqIT | N/A | N/A | N/A | Bronze | 21934 | 0.918557 | 17159.5 | N/A | N/A | N/A | N/A | N/A |
| Spectral Match to 1-Arachidoyl-2-hydroxy-sn-glycero-3-phosphocholine from NIST14 | 552.401 | 552.402 | M+H | 0.00097656 | 1.76785 | 21.7711667 | 1306.27 | 9 | HCD | N/A | N/A | N/A | Bronze | 23465 | 0.903776 | 41881.8 | N/A | N/A | N/A | N/A | N/A |
| Spectral Match to 1-Behenoyl-2-hydroxy-sn-glycero-3-phosphocholine from NIST14 | 580.43 | 580.433 | M+H | 0.0029907 | 5.1526 | 23.233 | 1393.98 | 7 | HCD | N/A | N/A | N/A | Bronze | 23918 | 0.885845 | 28214.9 | N/A | N/A | N/A | N/A | N/A |
| Spectral Match to 1-Heptadecanoyl-2-(5Z,8Z,11Z,14Z-eicosatetraenoyl)-sn-glycero-3-phosphocholine from NIST14 | 796.583 | 796.584 | M+H | 0.00097656 | 1.22594 | 24.0478333 | 1442.87 | 6 | QqIT | N/A | N/A | N/A | Bronze | 26219 | 0.805272 | 9303.34 | N/A | N/A | N/A | N/A | N/A |
| Spectral Match to 1-Heptadecanoyl-sn-glycero-3-phosphocholine from NIST14 | 510.355 | 510.356 | M+H | 0.00097656 | 1.9135 | 19.6988333 | 1181.93 | 8 | HCD | N/A | N/A | N/A | Bronze | 22697 | 0.915205 | 45213.8 | N/A | N/A | N/A | N/A | N/A |
| Spectral Match to 1-Hexadecanoyl-2-(5Z,8Z,11Z,14Z-eicosatetraenoyl)-sn-glycero-3-phosphoethanolamine from NIST14 | 740.525 | 740.522 | M+H | 0.0030518 | 4.12107 | 26.519 | 1591.14 | 20 | HCD | N/A | N/A | N/A | Bronze | 25667 | 0.865603 | 15103.9 | N/A | N/A | N/A | N/A | N/A |
| Spectral Match to 1-Hexadecanoyl-2-(9Z-octadecenoyl)-sn-glycero-3-phosphocholine from NIST14 | 760.586 | 760.585 | M+H | 0.00097656 | 1.28396 | 25.0201667 | 1501.21 | 9 | HCD | N/A | N/A | N/A | Bronze | 25864 | 0.942587 | 30588.6 | N/A | N/A | N/A | N/A | N/A |
| Spectral Match to 1-Hexadecanoyl-2-(9Z-octadecenoyl)-sn-glycero-3-phosphoethanolamine from NIST14 | 718.535 | 718.537 | M+H | 0.00201416 | 2.80315 | 27.1981667 | 1631.89 | 10 | HCD | N/A | N/A | N/A | Bronze | 25489 | 0.86093 | 5235.4 | N/A | N/A | N/A | N/A | N/A |
| Spectral Match to 1-Hexadecanoyl-2-octadecadienoyl-sn-glycero-3-phosphocholine from NIST14 | 758.569 | 758.569 | M+H | 0 | 0 | 22.4728333 | 1348.37 | 8 | Q-TOF | N/A | N/A | N/A | Bronze | 25820 | 0.944241 | 121388 | N/A | N/A | N/A | N/A | N/A |
| Spectral Match to 1-Hexadecanoyl-sn-glycero-3-phosphocholine from NIST14 | 518.321 | 518.322 | M+Na | 0.0010376 | 2.00184 | 19.0343333 | 1142.06 | 12 | Q-TOF | N/A | N/A | N/A | Bronze | 22847 | 0.845246 | 34613.5 | N/A | N/A | N/A | N/A | N/A |
| Spectral Match to 1-Hexadecanoyl-sn-glycerol from NIST14 | 331.284 | 331.285 | M+H | 0.0010071 | 3.03993 | 22.438 | 1346.28 | 11 | QqQ | N/A | N/A | N/A | Bronze | 12978 | 0.855089 | 27431.4 | N/A | N/A | N/A | N/A | N/A |
| Spectral Match to 1-Hexadecyl-2-(9Z-octadecenoyl)-sn-glycero-3-phosphocholine from NIST14 | 746.602 | 746.617 | M+H | 0.015015 | 20.1106 | 21.5505 | 1293.03 | 4 | HCD | N/A | N/A | N/A | Bronze | 25708 | 0.731706 | 2000 | N/A | N/A | N/A | N/A | N/A |
| Spectral Match to 1-Hexadecyl-sn-glycero-3-phosphocholine from NIST14 | 482.36 | 482.361 | M+H | 0.0010071 | 2.08782 | 19.6055 | 1176.33 | 10 | QqIT | N/A | N/A | N/A | Bronze | 22008 | 0.904939 | 48334.4 | N/A | N/A | N/A | N/A | N/A |
| Spectral Match to 1-Lignoceroyl-2-hydroxy-sn-glycero-3-phosphocholine from NIST14 | 608.463 | 608.465 | M+H | 0.0020142 | 3.31024 | 24.5645 | 1473.87 | 8 | HCD | N/A | N/A | N/A | Bronze | 24343 | 0.899524 | 23204.4 | N/A | N/A | N/A | N/A | N/A |
| Spectral Match to 1-Linoleoylglycerol from NIST14 | 355.284 | 355.285 | M+H | 0.0010071 | 2.83458 | 22.068 | 1324.08 | 9 | HCD | N/A | N/A | N/A | Bronze | 15725 | 0.701727 | 17124.7 | N/A | N/A | N/A | N/A | N/A |
| Spectral Match to 1-Myristoyl-sn-glycero-3-phosphocholine from NIST14 | 468.303 | 468.309 | M+H | 0.00598145 | 12.7726 | 17.9128333 | 1074.77 | 6 | Q-TOF | N/A | N/A | N/A | Bronze | 21502 | 0.900496 | 38502.2 | N/A | N/A | N/A | N/A | N/A |
| Spectral Match to 1-Octadecanoyl-2-(5Z,8Z,11Z,14Z-eicosatetraenoyl)-sn-glycero-3-phospho-(1'-myo-inositol) from NIST14 | 887.557 | 887.562 | M+H | 0.0050049 | 5.63894 | 23.2515 | 1395.09 | 11 | HCD | N/A | N/A | N/A | Bronze | 26779 | 0.648212 | 5478.84 | N/A | N/A | N/A | N/A | N/A |
| Spectral Match to 1-Octadecanoyl-2-(5Z,8Z,11Z,14Z-eicosatetraenoyl)-sn-glycero-3-phosphocholine from NIST14 | 810.6 | 810.6 | M+H | 0 | 0 | 24.5048333 | 1470.29 | 9 | HCD | N/A | N/A | N/A | Bronze | 26333 | 0.925268 | 45231.2 | N/A | N/A | N/A | N/A | N/A |
| Spectral Match to 1-Octadecanoyl-2-octadecenoyl-sn-glycero-3-phosphocholine from NIST14 | 788.612 | 788.617 | M+H | 0.0050049 | 6.34645 | 23.519 | 1411.14 | 5 | Q-TOF | N/A | N/A | N/A | Bronze | 26126 | 0.921647 | 38352.8 | N/A | N/A | N/A | N/A | N/A |
| Spectral Match to 1-Octadecanoyl-sn-glycero-3-phosphocholine from NIST14 | 546.35 | 546.353 | M+Na | 0.0030518 | 5.58572 | 20.4195 | 1225.17 | 8 | Q-TOF | N/A | N/A | N/A | Bronze | 23355 | 0.899768 | 33472.9 | N/A | N/A | N/A | N/A | N/A |
| Spectral Match to 1-O-Hexadecyl-2-O-(2E-butenoyl)-sn-glyceryl-3-phosphocholine from NIST14 | 550.39 | 550.39 | M+H | 0 | 0 | 20.957 | 1257.4 | 7 | HCD | N/A | N/A | N/A | Bronze | 23436 | 0.8159 | 60063 | N/A | N/A | N/A | N/A | N/A |
| Spectral Match to 1-Palmitoyl-2-docosahexaenoyl-sn-glycero-3-phosphocholine from NIST14 | 806.55 | 806.569 | M+H | 0.0189819 | 23.5347 | 20.4918333 | 1229.51 | 15 | HCD | N/A | N/A | N/A | Bronze | 26292 | 0.892698 | 20364.4 | N/A | N/A | N/A | N/A | N/A |
| Spectral Match to 1-Palmitoyl-2-hydroxy-sn-glycero-3-phosphoethanolamine from NIST14 | 454.293 | 454.295 | M+H | 0.0020142 | 4.43361 | 19.0595 | 1143.57 | 14 | HCD | N/A | N/A | N/A | Bronze | 20860 | 0.911127 | 21182.4 | N/A | N/A | N/A | N/A | N/A |
| Spectral Match to 1-Palmitoyl-2-myristoyl-sn-glycero-3-phosphocholine from NIST14 | 706.536 | 706.538 | M+H | 0.0020142 | 2.85075 | 27.1575 | 1629.45 | 10 | HCD | N/A | N/A | N/A | Bronze | 25383 | 0.933453 | 14864.8 | N/A | N/A | N/A | N/A | N/A |
| Spectral Match to 1-Palmitoyl-2-oleoyl-sn-glycerol from NIST14 | 595.534 | 595.529 | M+H | 0.00500488 | 8.40403 | 29.3263333 | 1759.58 | 13 | HCD | N/A | N/A | N/A | Bronze | 24171 | 0.823852 | 8027.95 | N/A | N/A | N/A | N/A | N/A |
| Spectral Match to 1-Pentadecanoyl-sn-glycero-3-phosphocholine from NIST14 | 482.323 | 482.324 | M+H | 0.00100708 | 2.08798 | 18.3426667 | 1100.56 | 9 | HCD | N/A | N/A | N/A | Bronze | 22000 | 0.906832 | 30382.8 | N/A | N/A | N/A | N/A | N/A |
| Spectral Match to 1-Stearoyl-2-hydroxy-sn-glycero-3-phosphocholine from NIST14 | 524.371 | 524.371 | M+H | 0 | 0 | 20.3891667 | 1223.35 | 8 | HCD | N/A | N/A | N/A | Bronze | 22972 | 0.91286 | 79724 | N/A | N/A | N/A | N/A | N/A |
| Spectral Match to 1-Stearoyl-2-hydroxy-sn-glycero-3-phosphoethanolamine from NIST14 | 504.305 | 504.307 | M+Na | 0.00201416 | 3.99393 | 20.3163333 | 1218.98 | 10 | HCD | N/A | N/A | N/A | Bronze | 22556 | 0.94303 | 12746 | N/A | N/A | N/A | N/A | N/A |
| Spectral Match to 1-Stearoyl-2-linoleoyl-sn-glycero-3-phosphocholine from NIST14 | 786.599 | 786.599 | M+H | 0 | 0 | 25.7091667 | 1542.55 | 5 | HCD | N/A | N/A | N/A | Bronze | 26107 | 0.934601 | 27526.4 | N/A | N/A | N/A | N/A | N/A |
| Spectral Match to 1-Stearoyl-2-linoleoyl-sn-glycero-3-phosphoethanolamine from NIST14 | 744.55 | 744.553 | M+H | 0.0029907 | 4.01682 | 27.488 | 1649.28 | 7 | HCD | N/A | N/A | N/A | Bronze | 25688 | 0.752441 | 1968.63 | N/A | N/A | N/A | N/A | N/A |
| Spectral Match to 1-Stearoyl-2-myristoyl-sn-glycero-3-phosphocholine from NIST14 | 734.564 | 734.569 | M+H | 0.0049439 | 6.73032 | 24.5705 | 1474.23 | 6 | HCD | N/A | N/A | N/A | Bronze | 25626 | 0.89898 | 30661.6 | N/A | N/A | N/A | N/A | N/A |
| Spectral Match to 2,4,7,9-Tetramethyl-5-decyne-4,7-diol from NIST14 | 191.18 | 191.179 | M+H-2H2O | 0.00099182 | 5.18789 | 16.933 | 1015.98 | 5 | QqQ | N/A | N/A | N/A | Bronze | 1001 | 0.753938 | 6394.56 | N/A | N/A | N/A | N/A | N/A |
| Spectral Match to 2-Butanone, 4-(2,6,6-trimethyl-2-cyclohexen-1-yl)- from NIST14 | 195.175 | 195.174 | M+H | 0.00100708 | 5.15988 | 14.4245333 | 865.472 | 6 | Q-TOF | N/A | N/A | N/A | Bronze | 1154 | 0.805519 | 88523.5 | N/A | N/A | N/A | N/A | N/A |
| Spectral Match to 2-Linoleoyl-1-palmitoyl-sn-glycero-3-phosphoethanolamine from NIST14 | 716.523 | 716.524 | M+H | 0.00097656 | 1.36292 | 26.504 | 1590.24 | 14 | HCD | N/A | N/A | N/A | Bronze | 25466 | 0.911502 | 15701 | N/A | N/A | N/A | N/A | N/A |
| Spectral Match to 3,5,7,3',4'-Pentahydroxyflavanone from NIST14 | 305.07 | 305.066 | M+H | 0.0039978 | 13.1045 | 11.2196167 | 673.177 | 9 | HCD | N/A | N/A | N/A | Bronze | 10044 | 0.957811 | 17971.8 | N/A | N/A | N/A | N/A | N/A |
| Spectral Match to 3-Hydroxycapric acid from NIST14 | 171.137 | 171.139 | M+H-H2O | 0.00201416 | 11.7693 | 12.2336167 | 734.017 | 4 | Q-TOF | N/A | N/A | N/A | Bronze | 545 | 0.685635 | 61459.3 | N/A | N/A | N/A | N/A | N/A |
| Spectral Match to 3-Indoleacrylic acid from NIST14 | 188.071 | 188.07 | M+H | 0.00099182 | 5.27365 | 13.1112833 | 786.677 | 4 | Q-TOF | N/A | N/A | N/A | Bronze | 913 | 0.938008 | 37767.8 | N/A | N/A | N/A | N/A | N/A |
| Spectral Match to 4-Hydroxynonenal from NIST14 | 157.122 | 157.122 | M+H | 0 | 0 | 17.1146667 | 1026.88 | 4 | HCD | N/A | N/A | N/A | Bronze | 286 | 0.609194 | 9583.65 | N/A | N/A | N/A | N/A | N/A |
| Spectral Match to 4-Hydroxynonenal glutathione from NIST14 | 464.204 | 464.205 | M+H | 0.00097656 | 2.10374 | 10.54195 | 632.517 | 6 | HCD | N/A | N/A | N/A | Bronze | 21247 | 0.701816 | 1811.29 | N/A | N/A | N/A | N/A | N/A |
| Spectral Match to 8-HETE from NIST14 | 303.231 | 303.233 | M+H-H2O | 0.00201416 | 6.64233 | 20.4306667 | 1225.84 | 11 | HCD | N/A | N/A | N/A | Bronze | 9802 | 0.813617 | 11758.2 | N/A | N/A | N/A | N/A | N/A |
| Spectral Match to (.+/-.)-8-Hydroxy-5Z,9E,11Z,14Z,17Z-eicosapentaenoic acid from NIST14 | 301.216 | 301.216 | M+H-H2O | 0 | 0 | 19.7388333 | 1184.33 | 8 | HCD | N/A | N/A | N/A | Bronze | 9565 | 0.670128 | 6282.51 | N/A | N/A | N/A | N/A | N/A |
| Spectral Match to 9-Octadecenamide, (Z)- from NIST14 | 563.547 | 563.551 | 2M+H | 0.00402832 | 7.14815 | 22.6461667 | 1358.77 | 9 | QqQ | N/A | N/A | N/A | Bronze | 23643 | 0.889663 | 34898.1 | N/A | N/A | N/A | N/A | N/A |
| Spectral Match to Abrine from NIST14 | 188.07 | 188.07 | M+H-CH3NH2 | 0 | 0 | 2.4117 | 144.702 | 6 | QqQ | N/A | N/A | N/A | Bronze | 908 | 0.986569 | 39524.7 | N/A | N/A | N/A | N/A | N/A |
| Spectral Match to Adenosine 5'-monophosphate from NIST14 | 370.052 | 370.05 | M+Na | 0.0020142 | 5.44291 | 0.19225 | 11.535 | 9 | HCD | N/A | N/A | N/A | Bronze | 16891 | 0.757766 | 1911.22 | N/A | N/A | N/A | N/A | N/A |
| Spectral Match to Arachidonic acid ethyl ester from NIST14 | 333.278 | 333.281 | M+H | 0.0029907 | 8.97366 | 25.754 | 1545.24 | 6 | HCD | N/A | N/A | N/A | Bronze | 13317 | 0.61645 | 2575.07 | N/A | N/A | N/A | N/A | N/A |
| Spectral Match to Arachidonic acid methyl ester from NIST14 | 319.262 | 319.265 | M+H | 0.0030212 | 9.4632 | 25.1805 | 1510.83 | 7 | HCD | N/A | N/A | N/A | Bronze | 11738 | 0.643035 | 7382.56 | N/A | N/A | N/A | N/A | N/A |
| Spectral Match to Arachidonoylthio-PC from NIST14 | 784.584 | 784.584 | M+H | 0 | 0 | 23.5651667 | 1413.91 | 11 | Q-TOF | N/A | N/A | N/A | Bronze | 26077 | 0.912308 | 69847 | N/A | N/A | N/A | N/A | N/A |
| Spectral Match to Azelaic acid from NIST14 | 189.112 | 189.112 | M+H | 0 | 0 | 11.6133833 | 696.803 | 6 | Q-TOF | N/A | N/A | N/A | Bronze | 956 | 0.944238 | 19340.3 | N/A | N/A | N/A | N/A | N/A |
| Spectral Match to Batyl alcohol from NIST14 | 345.334 | 345.336 | M+H | 0.00198364 | 5.74413 | 24.3606667 | 1461.64 | 5 | HCD | N/A | N/A | N/A | Bronze | 14743 | 0.626006 | 3209.79 | N/A | N/A | N/A | N/A | N/A |
| Spectral Match to Benzyltetradecyldimethylammonium from NIST14 | 332.331 | 332.331 | M+H | 0 | 0 | 20.2653333 | 1215.92 | 6 | qTof | CCCCCCCCCCCCCC[N+](C)(CC1=CC=CC=C1)C | N/A | N/A | Bronze | 13172 | 0.855713 | 18127.8 | WNBGYVXHFTYOBY-UHFFFAOYSA-N | WNBGYVXHFTYOBY | Benzenoids | Benzene and substituted derivatives | Phenylmethylamines |
| Spectral Match to Bifenthrin from NIST14 | 429.083 | 429.089 | 429.1 | 0.00598145 | 13.9401 | 12.2656167 | 735.937 | 4 | IT/ion trap | N/A | N/A | N/A | Bronze | 19858 | 0.683024 | 14750.4 | N/A | N/A | N/A | N/A | N/A |
| Spectral Match to Biliverdin from NIST14 | 583.255 | 583.257 | M+H | 0.0020142 | 3.45331 | 16.57635 | 994.581 | 13 | HCD | N/A | N/A | N/A | Bronze | 23965 | 0.844048 | 6580.79 | N/A | N/A | N/A | N/A | N/A |
| Spectral Match to Chloramphenicol from NIST14 | 323.02 | 323.022 | M+H | 0.0020142 | 6.2354 | 12.83315 | 769.989 | 8 | QqQ | N/A | N/A | N/A | Bronze | 12018 | 0.90427 | 1593.83 | N/A | N/A | N/A | N/A | N/A |
| Spectral Match to Cholesterol from NIST14 | 369.352 | 369.352 | M+H-H2O | 0 | 0 | 26.8768333 | 1612.61 | 15 | Q-TOF | N/A | N/A | N/A | Bronze | 16865 | 0.87766 | 63293 | N/A | N/A | N/A | N/A | N/A |
| Spectral Match to Cholic acid from NIST14 | 839.564 | 839.562 | 2M+Na | 0.00201416 | 2.39906 | 16.0983333 | 965.9 | 6 | QqQ | N/A | N/A | N/A | Bronze | 26545 | 0.938323 | 12520.5 | N/A | N/A | N/A | N/A | N/A |
| Spectral Match to cis-4,7,10,13,16,19-Docosahexaenoic acid from NIST14 | 329.247 | 329.249 | M+H | 0.0019836 | 6.02479 | 23.0625 | 1383.75 | 10 | HCD | N/A | N/A | N/A | Bronze | 12616 | 0.625516 | 34589.1 | N/A | N/A | N/A | N/A | N/A |
| Spectral Match to cis-5,8,11,14-Eicosatetraenoic acid from NIST14 | 305.247 | 305.248 | M+H | 0.00097656 | 3.19925 | 20.6025 | 1236.15 | 13 | HCD | N/A | N/A | N/A | Bronze | 10129 | 0.893122 | 35164.3 | N/A | N/A | N/A | N/A | N/A |
| Spectral Match to cis-7,10,13,16,19-Docosapentaenoic acid from NIST14 | 331.263 | 331.264 | M+H | 0.00100708 | 3.04012 | 23.4563333 | 1407.38 | 12 | HCD | N/A | N/A | N/A | Bronze | 12962 | 0.741781 | 13234.3 | N/A | N/A | N/A | N/A | N/A |
| Spectral Match to cis-7,10,13,16-Docosatetraenoic acid from NIST14 | 333.278 | 333.279 | M+H | 0.00097656 | 2.93017 | 24.1298333 | 1447.79 | 13 | HCD | N/A | N/A | N/A | Bronze | 13318 | 0.825849 | 11090.6 | N/A | N/A | N/A | N/A | N/A |
| Spectral Match to cis-8,11,14-Eicosatrienoic acid from NIST14 | 307.26 | 307.26 | M+H | 0 | 0 | 23.578 | 1414.7 | 12 | HCD | N/A | N/A | N/A | Bronze | 10287 | 0.8744 | 11764 | N/A | N/A | N/A | N/A | N/A |
| Spectral Match to cis-9-Hexadecenoic acid from NIST14 | 255.232 | 255.232 | M+H | 0 | 0 | 22.5845 | 1355.07 | 9 | HCD | N/A | N/A | N/A | Bronze | 5179 | 0.76635 | 8337.64 | N/A | N/A | N/A | N/A | N/A |
| Spectral Match to cis,cis-9,12-Octadecadien-1-ol from NIST14 | 267.268 | 267.268 | M+H | 0 | 0 | 23.7941667 | 1427.65 | 6 | HCD | N/A | N/A | N/A | Bronze | 5945 | 0.714341 | 3617.47 | N/A | N/A | N/A | N/A | N/A |
| Spectral Match to cis-Parinaric acid from NIST14 | 277.215 | 277.216 | M+H | 0.0010071 | 3.63285 | 21.696 | 1301.76 | 9 | IT/ion trap | N/A | N/A | N/A | Bronze | 6962 | 0.670197 | 2146.53 | N/A | N/A | N/A | N/A | N/A |
| Spectral Match to Conjugated linoleic acid (9E,11E) from NIST14 | 263.237 | 263.237 | M+H-H2O | 0 | 0 | 23.0805 | 1384.83 | 6 | HCD | N/A | N/A | N/A | Bronze | 5624 | 0.701717 | 13769.7 | N/A | N/A | N/A | N/A | N/A |
| Spectral Match to Cyclohexasiloxane, dodecamethyl- from NIST14-Contaminant | 445.121 | 445.122 | M+H | 0.0010071 | 2.26249 | 0.5691 | 34.146 | 9 | QqQ | N/A | N/A | N/A | Bronze | 20595 | 0.910076 | 226339 | N/A | N/A | N/A | N/A | N/A |
| Spectral Match to Cyclopentasiloxane, decamethyl- from NIST14-Contaminant | 371.115 | 371.103 | M+H | 0.0119934 | 32.3172 | 0.59293333 | 35.576 | 10 | QqQ | N/A | N/A | N/A | Bronze | 16952 | 0.88604 | 247210 | N/A | N/A | N/A | N/A | N/A |
| Spectral Match to Decaethylene glycol from NIST14-Contaminant | 459.28 | 459.28 | M+H | 0 | 0 | 10.4838333 | 629.03 | 4 | QqQ | N/A | N/A | N/A | Bronze | 21151 | 0.806264 | 27343 | N/A | N/A | N/A | N/A | N/A |
| Spectral Match to D-erythro-C18-Sphingosine from NIST14 | 300.289 | 300.29 | M+H | 0.00100708 | 3.3537 | 17.7168333 | 1063.01 | 9 | QqQ | N/A | N/A | N/A | Bronze | 9213 | 0.913984 | 10338.3 | N/A | N/A | N/A | N/A | N/A |
| Spectral Match to D-erythro-Sphinganine from NIST14 | 302.304 | 302.306 | M+H | 0.00201416 | 6.6627 | 17.9991667 | 1079.95 | 7 | Q-TOF | N/A | N/A | N/A | Bronze | 9739 | 0.817823 | 9593.48 | N/A | N/A | N/A | N/A | N/A |
| Spectral Match to D-erythro-Sphingosine-1-phosphate from NIST14 | 380.256 | 380.256 | M+H | 0 | 0 | 17.832 | 1069.92 | 10 | HCD | N/A | N/A | N/A | Bronze | 17406 | 0.92066 | 5823.9 | N/A | N/A | N/A | N/A | N/A |
| Spectral Match to D-Fructose from NIST14 | 198.094 | 198.09 | M+NH4 | 0.0039978 | 20.1813 | 1.09728333 | 65.837 | 5 | QqQ | N/A | N/A | N/A | Bronze | 1236 | 0.641008 | 6822.93 | N/A | N/A | N/A | N/A | N/A |
| Spectral Match to Dibutyl phthalate from NIST14-Contaminant | 279.16 | 279.172 | M+H | 0.011993 | 42.9625 | 0.7744 | 46.464 | 4 | QqQ | N/A | N/A | N/A | Bronze | 7048 | 0.897262 | 248302 | N/A | N/A | N/A | N/A | N/A |
| Spectral Match to Didodecyl 3,3'-thiodipropionate oxide from NIST14 | 531.406 | 531.408 | M+H | 0.00201416 | 3.79025 | 27.3876667 | 1643.26 | 10 | QqQ | N/A | N/A | N/A | Bronze | 23133 | 0.886015 | 23968.5 | N/A | N/A | N/A | N/A | N/A |
| Spectral Match to Diethyl phthalate from NIST14-Contaminant | 223.097 | 223.112 | M+H | 0.014999 | 67.2326 | 2.1119 | 126.714 | 4 | QqQ | N/A | N/A | N/A | Bronze | 2745 | 0.827737 | 196310 | N/A | N/A | N/A | N/A | N/A |
| Spectral Match to Dioctyl phthalate from NIST14-Contaminant | 391.287 | 391.285 | M+H | 0.00198364 | 5.06953 | 25.9323333 | 1555.94 | 5 | QqQ | N/A | N/A | N/A | Bronze | 17914 | 0.85476 | 3619.48 | N/A | N/A | N/A | N/A | N/A |
| Spectral Match to DL-Indole-3-lactic acid from NIST14 | 206.081 | 206.081 | M+H | 0 | 0 | 10.8596 | 651.573 | 6 | Q-TOF | N/A | N/A | N/A | Bronze | 1813 | 0.99492 | 58124.5 | N/A | N/A | N/A | N/A | N/A |
| Spectral Match to DL-Phenylalanine from NIST14 | 331.168 | 331.166 | 2M+H | 0.0020142 | 6.08199 | 1.17925 | 70.755 | 4 | Q-TOF | N/A | N/A | N/A | Bronze | 12799 | 0.833262 | 32211.6 | N/A | N/A | N/A | N/A | N/A |
| Spectral Match to Docosahexaenoyl PAF C-16 from NIST14 | 792.551 | 792.57 | M+H | 0.0189819 | 23.9504 | 24.0828333 | 1444.97 | 6 | HCD | N/A | N/A | N/A | Bronze | 26153 | 0.840616 | 11522.9 | N/A | N/A | N/A | N/A | N/A |
| Spectral Match to Dodecanedioic acid from NIST14 | 231.158 | 231.159 | M+H | 0.00099182 | 4.29066 | 14.7794667 | 886.768 | 5 | QqQ | N/A | N/A | N/A | Bronze | 3178 | 0.631428 | 18746.9 | N/A | N/A | N/A | N/A | N/A |
| Spectral Match to Eicosapentaenoic acid ethyl ester from NIST14 | 331.263 | 331.257 | M+H | 0.006012 | 18.1486 | 23.7695 | 1426.17 | 8 | HCD | N/A | N/A | N/A | Bronze | 12946 | 0.608036 | 2333.48 | N/A | N/A | N/A | N/A | N/A |
| Spectral Match to Eicosapentaenoyl PAF C-16 from NIST14 | 766.572 | 766.572 | M+H | 0 | 0 | 23.4798333 | 1408.79 | 6 | HCD | N/A | N/A | N/A | Bronze | 25928 | 0.82742 | 7384.67 | N/A | N/A | N/A | N/A | N/A |
| Spectral Match to Elaidic acid from NIST14 | 283.263 | 283.263 | M+H | 0 | 0 | 23.8893333 | 1433.36 | 12 | QqQ | N/A | N/A | N/A | Bronze | 7289 | 0.88755 | 22848.8 | N/A | N/A | N/A | N/A | N/A |
| Spectral Match to Enterolactone from NIST14 | 299.128 | 299.129 | M+H | 0.00100708 | 3.36672 | 15.0896667 | 905.38 | 5 | HCD | N/A | N/A | N/A | Bronze | 9050 | 0.629514 | 5004.68 | N/A | N/A | N/A | N/A | N/A |
| Spectral Match to Equol from NIST14 | 243.101 | 243.101 | M+H | 0 | 0 | 11.9664167 | 717.985 | 4 | Q-TOF | N/A | N/A | N/A | Bronze | 4385 | 0.774752 | 27983.1 | N/A | N/A | N/A | N/A | N/A |
| Spectral Match to Ethanol, 2-(2-butoxyethoxy)- from NIST14 | 163.133 | 163.124 | M+H | 0.00900269 | 55.1862 | 20.4251667 | 1225.51 | 4 | QqQ | N/A | N/A | N/A | Bronze | 342 | 0.609739 | 3734.69 | N/A | N/A | N/A | N/A | N/A |
| Spectral Match to Ethylenediaminetetraacetic acid from NIST14 | 293.097 | 293.101 | M+H | 0.0040283 | 13.744 | 1.3005 | 78.03 | 6 | Q-TOF | N/A | N/A | N/A | Bronze | 8145 | 0.912891 | 163126 | N/A | N/A | N/A | N/A | N/A |
| Spectral Match to .gamma.-CEHC from NIST14 | 265.143 | 265.143 | M+H | 0 | 0 | 14.7482 | 884.889 | 5 | Q-TOF | N/A | N/A | N/A | Bronze | 5757 | 0.76786 | 4158.4 | N/A | N/A | N/A | N/A | N/A |
| Spectral Match to Glu Phe from METLIN | 295.128 | 295.129 | M+H | 0.00100708 | 3.41235 | 0.89473333 | 53.684 | 8 | qTof | O=C(O)[C@H](CC1=CC=CC=C1)NC(CC[C@H](N)C(O)=O)=O | InChI=1S/C14H18N2O5/c15-10(13(18)19)6-7-12(17)16-11(14(20)21)8-9-4-2-1-3-5-9/h1-5,10-11H,6-8,15H2,(H,16,17)(H,18,19)(H,20,21)/t10-,11-/m0/s1 | N/A | Bronze | 8411 | 0.883242 | 48182.2 | XHHOHZPNYFQJKL-QWRGUYRKSA-N | XHHOHZPNYFQJKL | Organic acids and derivatives | Carboxylic acids and derivatives | Amino acids, peptides, and analogues |
| Spectral Match to Glutathione, oxidized from NIST14 | 307.083 | 307.069 | M+2H] | 0.014008 | 45.6149 | 1.72845 | 103.707 | 14 | Q-TOF | N/A | N/A | N/A | Bronze | 10217 | 0.629175 | 5239.16 | N/A | N/A | N/A | N/A | N/A |
| Spectral Match to Glycerol 1-stearate from NIST14 | 359.316 | 359.316 | M+H | 0 | 0 | 23.777 | 1426.62 | 11 | Q-TOF | N/A | N/A | N/A | Bronze | 16093 | 0.8308 | 70272.8 | N/A | N/A | N/A | N/A | N/A |
| Spectral Match to Glycocholic acid from NIST14 | 466.316 | 466.316 | M+H | 0 | 0 | 14.8287167 | 889.723 | 14 | Q-TOF | N/A | N/A | N/A | Bronze | 21308 | 0.916018 | 29509 | N/A | N/A | N/A | N/A | N/A |
| Spectral Match to Glycoursodeoxycholic acid from NIST14 | 432.311 | 432.309 | M+H-H2O | 0.00201416 | 4.65905 | 16.4471833 | 986.831 | 11 | HCD | N/A | N/A | N/A | Bronze | 20078 | 0.736519 | 1380.77 | N/A | N/A | N/A | N/A | N/A |
| Spectral Match to Hemin cation from NIST14 | 616.177 | 616.176 | Cat | 0.00097656 | 1.58487 | 16.9793333 | 1018.76 | 5 | IT/ion trap | N/A | N/A | N/A | Bronze | 24465 | 0.908059 | 43146.2 | N/A | N/A | N/A | N/A | N/A |
| Spectral Match to Hexaethylene glycol from NIST14-Contaminant | 283.175 | 283.175 | M+H | 0 | 0 | 8.24733333 | 494.84 | 6 | HCD | N/A | N/A | N/A | Bronze | 7272 | 0.914052 | 43907.8 | N/A | N/A | N/A | N/A | N/A |
| Spectral Match to Inosine from NIST14 | 269.087 | 269.089 | M+H | 0.00198364 | 7.37175 | 1.17423333 | 70.454 | 4 | QqQ | N/A | N/A | N/A | Bronze | 5996 | 0.904525 | 9983.06 | N/A | N/A | N/A | N/A | N/A |
| Spectral Match to Isovalerylcarnitine from NIST14 | 246.17 | 246.17 | M+H | 0 | 0 | 6.20701667 | 372.421 | 4 | IT/ion trap | N/A | N/A | N/A | Bronze | 4579 | 0.777499 | 62655.6 | N/A | N/A | N/A | N/A | N/A |
| Spectral Match to Jasmonic acid from NIST14 | 211.133 | 211.133 | M+H | 0 | 0 | 14.3072 | 858.429 | 7 | Q-TOF | N/A | N/A | N/A | Bronze | 2187 | 0.81363 | 1461.43 | N/A | N/A | N/A | N/A | N/A |
| Spectral Match to L-Kynurenine from NIST14 | 209.092 | 209.097 | M+H | 0.00500488 | 23.9363 | 1.43098333 | 85.859 | 7 | Q-TOF | N/A | N/A | N/A | Bronze | 1990 | 0.947633 | 54493.9 | N/A | N/A | N/A | N/A | N/A |
| Spectral Match to L-Phenylalanine, N-acetyl- from NIST14 | 208.097 | 208.096 | M+H | 0.00100708 | 4.83947 | 10.4574333 | 627.446 | 4 | Q-TOF | N/A | N/A | N/A | Bronze | 1976 | 0.862537 | 10208.6 | N/A | N/A | N/A | N/A | N/A |
| Spectral Match to L-Tryptophan from NIST14 | 205.098 | 205.097 | M+H | 0.00100708 | 4.91024 | 2.45321667 | 147.193 | 5 | QqQ | N/A | N/A | N/A | Bronze | 1794 | 0.913652 | 8000 | N/A | N/A | N/A | N/A | N/A |
| Spectral Match to L-Tyrosine from NIST14 | 182.081 | 182.081 | M+H | 0 | 0 | 0.30405 | 18.243 | 6 | QqQ | N/A | N/A | N/A | Bronze | 719 | 0.99448 | 101998 | N/A | N/A | N/A | N/A | N/A |
| Spectral Match to Lyso-PAF C-18 from NIST14 | 510.391 | 510.391 | M+H | 0 | 0 | 20.9806667 | 1258.84 | 7 | Q-TOF | N/A | N/A | N/A | Bronze | 22699 | 0.751559 | 22683.1 | N/A | N/A | N/A | N/A | N/A |
| Spectral Match to Lyso-PC(16:0) from NIST14 | 496.34 | 496.34 | M+H | 0 | 0 | 19.0445 | 1142.67 | 8 | Q-TOF | N/A | N/A | N/A | Bronze | 22369 | 0.93196 | 77530 | N/A | N/A | N/A | N/A | N/A |
| Spectral Match to Monoelaidin from NIST14 | 357.299 | 357.301 | M+H | 0.00198364 | 5.55177 | 22.9471667 | 1376.83 | 12 | HCD | N/A | N/A | N/A | Bronze | 15874 | 0.876063 | 13884.4 | N/A | N/A | N/A | N/A | N/A |
| Spectral Match to N-Oleoyl-D-erythro-sphingosylphosphorylcholine from NIST14 | 729.59 | 729.591 | M+H | 0.00097656 | 1.33851 | 25.8211667 | 1549.27 | 6 | HCD | N/A | N/A | N/A | Bronze | 25576 | 0.924293 | 19020.1 | N/A | N/A | N/A | N/A | N/A |
| Spectral Match to N-Oleoylethanolamine from NIST14 | 326.305 | 326.305 | M+H | 0 | 0 | 21.871 | 1312.26 | 7 | Q-TOF | N/A | N/A | N/A | Bronze | 12348 | 0.65337 | 3612.99 | N/A | N/A | N/A | N/A | N/A |
| Spectral Match to Nonaethylene glycol from NIST14-Contaminant | 415.253 | 415.253 | M+H | 0 | 0 | 10.1393333 | 608.36 | 11 | QqQ | N/A | N/A | N/A | Bronze | 19295 | 0.942368 | 25674.4 | N/A | N/A | N/A | N/A | N/A |
| Spectral Match to N-Tetracosanoyl-4-sphingenyl-1-O-phosphorylcholine from NIST14 | 815.708 | 815.698 | M+H | 0.01001 | 12.2713 | 22.884 | 1373.04 | 5 | Q-TOF | N/A | N/A | N/A | Bronze | 26388 | 0.835512 | 3413.91 | N/A | N/A | N/A | N/A | N/A |
| Spectral Match to N-Tetracosenoyl-4-sphingenyl-1-O-phosphorylcholine from NIST14 | 813.683 | 813.684 | M+H | 0.0010376 | 1.27519 | 22.1508333 | 1329.05 | 6 | Q-TOF | N/A | N/A | N/A | Bronze | 26361 | 0.92206 | 38942.6 | N/A | N/A | N/A | N/A | N/A |
| Spectral Match to Octadecanamide from NIST14 | 284.3 | 284.296 | M+H | 0.0039978 | 14.0619 | 23.5248333 | 1411.49 | 10 | QqQ | N/A | N/A | N/A | Bronze | 7344 | 0.868619 | 7926.25 | N/A | N/A | N/A | N/A | N/A |
| Spectral Match to Palatinose from NIST14 | 360.15 | 360.151 | M+NH4 | 0.00100708 | 2.79628 | 0.67658333 | 40.595 | 5 | QqQ | N/A | N/A | N/A | Bronze | 16124 | 0.734465 | 1389.98 | N/A | N/A | N/A | N/A | N/A |
| Spectral Match to Palmitamide from NIST14 | 256.264 | 256.264 | M+H | 0 | 0 | 22.1391667 | 1328.35 | 9 | HCD | N/A | N/A | N/A | Bronze | 5237 | 0.854574 | 13350.5 | N/A | N/A | N/A | N/A | N/A |
| Spectral Match to Pentapropylene glycol from NIST14-Contaminant | 309.227 | 309.226 | M+H | 0.00097656 | 3.15808 | 12.1569167 | 729.415 | 5 | Q-TOF | N/A | N/A | N/A | Bronze | 10442 | 0.653407 | 1334.61 | N/A | N/A | N/A | N/A | N/A |
| Spectral Match to Perillaldehyde from NIST14 | 169.122 | 169.122 | M+H+H2O | 0 | 0 | 10.3348 | 620.088 | 4 | IT/ion trap | N/A | N/A | N/A | Bronze | 388 | 0.762134 | 4212.38 | N/A | N/A | N/A | N/A | N/A |
| Spectral Match to Propanoic acid, 3,3'-thiobis-, didodecyl ester from NIST14 | 515.41 | 515.416 | M+H | 0.0060425 | 11.7236 | 29.038 | 1742.28 | 5 | QqQ | N/A | N/A | N/A | Bronze | 22792 | 0.825704 | 1991.65 | N/A | N/A | N/A | N/A | N/A |
| Spectral Match to p-tert-Octylphenol decaglycol ether from NIST14-Contaminant | 647.434 | 647.433 | M+H | 0.001038 | 1.60263 | 20.3265 | 1219.59 | 11 | QqQ | N/A | N/A | N/A | Bronze | 24828 | 0.815891 | 2678.55 | N/A | N/A | N/A | N/A | N/A |
| Spectral Match to p-tert-Octylphenol heptaglycol ether from NIST14-Contaminant | 515.355 | 515.357 | M+H | 0.00201416 | 3.9083 | 20.3703333 | 1222.22 | 17 | QqQ | N/A | N/A | N/A | Bronze | 22791 | 0.904182 | 2762.96 | N/A | N/A | N/A | N/A | N/A |
| Spectral Match to p-tert-Octylphenol hexaglycol ether from NIST14-Contaminant | 471.332 | 471.331 | M+H | 0.00100708 | 2.13667 | 20.3898333 | 1223.39 | 13 | QqQ | N/A | N/A | N/A | Bronze | 21593 | 0.849042 | 2656.44 | N/A | N/A | N/A | N/A | N/A |
| Spectral Match to p-tert-Octylphenol nonaglycol ether from NIST14-Contaminant | 603.406 | 603.408 | M+H | 0.0020142 | 3.33799 | 20.324 | 1219.44 | 12 | QqQ | N/A | N/A | N/A | Bronze | 24269 | 0.866471 | 2808.58 | N/A | N/A | N/A | N/A | N/A |
| Spectral Match to p-tert-Octylphenol undecaglycol ether from NIST14-Contaminant | 691.46 | 691.459 | M+H | 0.001038 | 1.50059 | 20.329 | 1219.74 | 13 | QqQ | N/A | N/A | N/A | Bronze | 25239 | 0.792908 | 1560.2 | N/A | N/A | N/A | N/A | N/A |
| Spectral Match to PyroGlu-Phe from NIST14 | 277.119 | 277.117 | M+H | 0.00198364 | 7.15809 | 10.2741333 | 616.448 | 5 | Q-TOF | N/A | N/A | N/A | Bronze | 6875 | 0.693608 | 1616.74 | N/A | N/A | N/A | N/A | N/A |
| Spectral Match to Retinol from NIST14 | 269.226 | 269.227 | M+H-H2O | 0.00097656 | 3.6273 | 23.0348333 | 1382.09 | 9 | Q-TOF | N/A | N/A | N/A | Bronze | 6070 | 0.801302 | 22554.3 | N/A | N/A | N/A | N/A | N/A |
| Spectral Match to Ricinoleic acid methyl ester from NIST14 | 295.262 | 295.265 | M+H-H2O | 0.00302124 | 10.2324 | 25.3313333 | 1519.88 | 8 | HCD | N/A | N/A | N/A | Bronze | 8589 | 0.669258 | 2669.04 | N/A | N/A | N/A | N/A | N/A |
| Spectral Match to Suberic acid from NIST14 | 175.096 | 175.096 | M+H | 0 | 0 | 10.0132 | 600.792 | 5 | Q-TOF | N/A | N/A | N/A | Bronze | 619 | 0.87214 | 18370.5 | N/A | N/A | N/A | N/A | N/A |
| Spectral Match to Taurocholic acid from NIST14 | 538.293 | 538.28 | M+Na | 0.013001 | 24.1513 | 14.46275 | 867.765 | 7 | Q-TOF | N/A | N/A | N/A | Bronze | 23223 | 0.727446 | 12584.5 | N/A | N/A | N/A | N/A | N/A |
| Spectral Match to Thr-Val-Leu from NIST14 | 332.218 | 332.218 | M+H | 0 | 0 | 9.73688333 | 584.213 | 5 | QqQ | N/A | N/A | N/A | Bronze | 13082 | 0.663158 | 1076.84 | N/A | N/A | N/A | N/A | N/A |
| Spectral Match to trans-EKODE-(E)-Ib from NIST14 | 311.221 | 311.223 | M+H | 0.00198364 | 6.37374 | 19.3861667 | 1163.17 | 7 | qTof | CCCCCC1C(/C=C/C(CCCCCCCC(O)=O)=O)O1 | InChI=1S/C18H30O4/c1-2-3-7-11-16-17(22-16)14-13-15(19)10-8-5-4-6-9-12-18(20)21/h13-14,16-17H,2-12H2,1H3,(H,20,21)/b14-13+ | N/A | Bronze | 10593 | 0.856204 | 11145.6 | RCMABBHQYMBYKV-BUHFOSPRSA-N | RCMABBHQYMBYKV | Lipids and lipid-like molecules | Fatty Acyls | Fatty acids and conjugates |
| Spectral Match to Tris(2-butoxyethyl) phosphate from NIST14 | 421.231 | 421.233 | M+Na | 0.00201416 | 4.7816 | 20.4463333 | 1226.78 | 5 | HCD | N/A | N/A | N/A | Bronze | 19497 | 0.86784 | 4822.4 | N/A | N/A | N/A | N/A | N/A |
| Spectral Match to Undecaethylene glycol from NIST14-Contaminant | 503.306 | 503.306 | M+H | 0 | 0 | 10.8144667 | 648.868 | 9 | QqQ | N/A | N/A | N/A | Bronze | 22537 | 0.890541 | 22839.4 | N/A | N/A | N/A | N/A | N/A |
| Spectral Match to Val-Leu from NIST14 | 213.16 | 213.16 | M+H-H2O | 0 | 0 | 11.07775 | 664.665 | 5 | Q-TOF | N/A | N/A | N/A | Bronze | 2304 | 0.64644 | 2048.67 | N/A | N/A | N/A | N/A | N/A |
| Spectral Match to Xanthosine from NIST14 | 285.083 | 285.083 | M+H | 0 | 0 | 1.17401667 | 70.441 | 8 | Q-TOF | N/A | N/A | N/A | Bronze | 7406 | 0.856467 | 6964.77 | N/A | N/A | N/A | N/A | N/A |
| Spectral Match to Zymosterol from NIST14 | 367.336 | 367.337 | M+H-H2O | 0.00100708 | 2.74158 | 26.1138333 | 1566.83 | 12 | HCD | N/A | N/A | N/A | Bronze | 16694 | 0.723551 | 10267.3 | N/A | N/A | N/A | N/A | N/A |
| Sphingomyelin (18:1/14:0) | 675.536 | 675.543 | M+H | 0.00701904 | 10.3903 | 24.1946667 | 1451.68 | 6 | qTof |  |  |  | Bronze | 25114 | 0.833668 | 13779.2 | N/A | N/A | N/A | N/A | N/A |
| Succinoadenosine | 384.12 | 384.115 | M+H | 0.00500488 | 13.0295 | 1.45736667 | 87.442 | 7 | qTof |  |  |  | Bronze | 17644 | 0.876244 | 14349.6 | N/A | N/A | N/A | N/A | N/A |
| taurocholic acid | 498.287 | 498.287 | M-H2O+H | 0 | 0 | 14.4812333 | 868.874 | 13 | Orbitrap | C[C@H](CCC(=O)NCCS(=O)(=O)O)[C@H]1CC[C@@H]2[C@@]1([C@H](C[C@H]3[C@H]2[C@@H](C[C@H]4[C@@]3(CC[C@H](C4)O)C)O)O)C | 1S/C26H45NO7S/c1-15(4-7-23(31)27-10-11-35(32,33)34)18-5-6-19-24-20(14-22(30)26(18,19)3)25(2)9-8-17(28)12-16(25)13-21(24)29/h15-22,24,28-30H,4-14H2,1-3H3,(H,27,31)(H,32,33,34)/t15?,16-,17+,18+,19-,20-,21+,22-,24-,25?,26?/m0/s1 | N/A | Gold | 22424 | 0.745712 | 5343.23 | WBWWGRHZICKQGZ-HZAMXZRMSA-N | WBWWGRHZICKQGZ | Lipids and lipid-like molecules | Steroids and steroid derivatives | Bile acids, alcohols and derivatives |
| taurodeoxycholic acid | 522.286 | 522.285 | M+Na | 0.001038 | 1.98665 | 15.78175 | 946.905 | 5 | Orbitrap | C[C@H](CCC(=O)NCCS(=O)(=O)O)[C@H]1CC[C@@H]2[C@@]1([C@H](C[C@H]3[C@H]2CC[C@H]4[C@@]3(CC[C@H](C4)O)C)O)C | 1S/C26H45NO6S/c1-16(4-9-24(30)27-12-13-34(31,32)33)20-7-8-21-19-6-5-17-14-18(28)10-11-25(17,2)22(19)15-23(29)26(20,21)3/h16-23,28-29H,4-15H2,1-3H3,(H,27,30)(H,31,32,33)/t16?,17-,18-,19+,20-,21+,22+,23+,25?,26?/m1/s1 | N/A | Gold | 22918 | 0.614056 | 1879.35 | AWDRATDZQPNJFN-VAYUFCLWSA-N | AWDRATDZQPNJFN | Lipids and lipid-like molecules | Steroids and steroid derivatives | Bile acids, alcohols and derivatives |
| TOP19 Psoriasis feature - Unknown FeatureID=3668 | 466.328 | 466.33 | M+H | 0.0019836 | 4.25375 | 20.721 | 1243.26 | 8 | qTof |  |  |  | Bronze | 21313 | 0.791198 | 11115.1 | N/A | N/A | N/A | N/A | N/A |
| TOP 8 Psoriasis feature - Unknown FeatureID=4262 | 438.297 | 438.299 | M+H | 0.0020142 | 4.59542 | 19.447 | 1166.82 | 9 | qTof |  |  |  | Bronze | 20385 | 0.84838 | 9420.14 | N/A | N/A | N/A | N/A | N/A |
| URSODEOXYCHOLATE | 357.279 | 357.279 | M-2H2O+H | 0 | 0 | 17.8596667 | 1071.58 | 11 | Orbitrap | CC(CCC(=O)O)C1CCC2C1(CCC3C2C(CC4C3(CCC(C4)O)C)O)C | InChI=1S/C24H40O4/c1-14(4-7-21(27)28)17-5-6-18-22-19(9-11-24(17,18)3)23(2)10-8-16(25)12-15(23)13-20(22)26/h14-20,22,25-26H,4-13H2,1-3H3,(H,27,28) | N/A | Gold | 15850 | 0.755814 | 16219.4 | RUDATBOHQWOJDD-UHFFFAOYSA-N | RUDATBOHQWOJDD | Lipids and lipid-like molecules | Steroids and steroid derivatives | Bile acids, alcohols and derivatives |
| VALINE | 118.086 | 118.087 | M+H | 0.00099945 | 8.46375 | 0.41901667 | 25.141 | 4 | Orbitrap | CC(C)[C@H](N)C(O)=O | "InChI=1S/C5H11NO2/c1-3(2)4(6)5(7)8/h3-4H,6H2,1-2H3,(H,7,8)/t4-/m0/s1" | N/A | Gold | 75 | 0.958134 | 32984.2 | KZSNJWFQEVHDMF-BYPYZUCNSA-N | KZSNJWFQEVHDMF | Organic acids and derivatives | Carboxylic acids and derivatives | Amino acids, peptides, and analogues |
| Yersiniabactin_Fe-adduct | 535.035 | 535.044 | M+H | 0.0090332 | 16.8834 | 14.0627167 | 843.763 | 9 | Orbitrap |  |  |  | Bronze | 23175 | 0.859602 | 2668.29 | N/A | N/A | N/A | N/A | N/A |

**Table S3. List of annotated metabolites with differential abundance among Gulf War Illness (GWI) and control cohorts.**

| **Compound Name** | **Chemical Class** | **Fold Change**  **(GWI/control)** | **Retention Time** | **Experimental mass** | **[Adduct]** | **Exact**  **mass** | **Mass Error (ppm)** | **p value** | **Putative**  **Structure** |
| --- | --- | --- | --- | --- | --- | --- | --- | --- | --- |
| Cystine | Carboxylic acids and derivatives | 0.1 | 0.6897 | 241.0314 | [M+H]^+^ | 241.0317 | -1.2447 | 0.0146 |  |
| Pyridoxine | Pyridines and derivatives | 0.15 | 0.9359 | 170.0818 | [M+H]^+^ | 170.0818 | 0.0000 | 0.0103 |  |
| Succinoadenosine | Purine nucleosides | 0.16 | 1.3834 | 384.1154 | [M+H]^+^ | 384.1151 | 0.7810 | 0.0189 |  |
| L-Glu-L-Tyr (Alpha-Glutamyltyrosine) | Carboxylic acids and derivatives | 0.19 | 1.0108 | 311.1248 | [M+H]^+^ | 311.1244 | 1.2857 | 0.0112 |  |
| 3-Keto-9Z,11E-octadecadienoic acid | Fatty Acyls | 0.2 | 14.7299 | 295.2270 | [M+H]^+^ | 295.2274 | -1.3549 | 0.0420 |  |
| 3-Hydroxycapric acid | Hydroxy acids and derivatives | 0.22 | 12.5007 | 189.1484 | [M+H]^+^ | 189.1491 | -3.7008 | 0.0156 |  |
| 12(13)-Epoxy-9Z-octadecenoic acid | Fatty Acyls | 0.23 | 17.7107 | 297.2426 | [M+H]^+^ | 297.2430 | -1.3457 | 0.0146 |  |
| 1-Octadecanoyl-2-octadecenoyl-sn-glycero-3-phosphocholine (1-Stearoyl-2-oleoyl-sn-glycero-3-phosphocholine) | Glycerophospholipids | 0.28 | 23.8787 | 788.6135 | [M+H]^+^ | 788.6170 | -4.4381 | 0.0425 |  |
| L-Tyrosine | Carboxylic acids and derivatives | 0.35 | 0.9715 | 165.0547 | [M-NH3+H]^+^ | 165.0552 | -3.0293 | 0.0255 |  |
| L-Tryptophan | Carboxylic acids and derivatives | 0.35 | 10.8685 | 188.0704 | [M-NH3+H]^+^ | 188.0712 | -4.2537 | 0.0353 |  |
| Valine | Carboxylic acids and derivatives | 0.46 | 0.5798 | 118.0867 | [M+H]^+^ | 118.0869 | -1.6937 | 0.0346 |  |
| 13S-Hydroxy-9Z,11E,15Z-octadecatrienoic acid | Fatty Acyls | 0.52 | 14.7127 | 277.2161 | [M-H2O+H]^+^ | 277.2168 | -2.5251 | 0.0184 |  |
| 3-Hydroxycapric acid | Hydroxy acids and derivatives | 0.55 | 15.4875 | 171.1380 | [M-H2O+H]^+^ | 171.1386 | -3.5059 | 0.0091 |  |
| sn-Glycero-3-phosphocholine | Glycerophospholipids | 1.4 | 0.7821 | 258.1098 | [M+H]^+^ | 258.1106 | -3.0994 | 0.0308 |  |
| L-Kynurenine | Organooxygen compounds | 1.5 | 2.0589 | 209.0921 | [M+H]^+^ | 209.0927 | -2.8695 | 0.0269 |  |
| 1-Palmitoyl-2-oleoyl-sn-glycerol | Glycerolipids | 2.5 | 29.3513 | 595.5284 | [M+H]^+^ | 595.5301 | -2.8546 | 0.0418 |  |
| Palmitoylcarnitine | Fatty Acyls | 2.9 | 19.3608 | 400.3421 | [M+H]^+^ | 400.3425 | -0.9991 | 0.0459 |  |
| 1-Pentadecanoyl-sn-glycero-3-phosphocholine | Glycerophospholipids | 3.0 | 18.5374 | 482.3234 | [M+H]^+^ | 482.3247 | -2.6953 | 0.0485 |  |
| 1-Pentadecanoyl-sn-glycero-3-phosphocholine | Glycerophospholipids | 3.0 | 18.5374 | 482.3234 | [M+H]^+^ | 482.3247 | -2.6953 | 0.0485 |  |
| 1-Hexadecanoyl-2-(5Z,8Z,11Z,14Z-eicosatetraenoyl)-sn-glycero-3-phosphoethanolamine | Glycerophospholipids | 3.0 | 26.3287 | 740.5207 | [M+H]^+^ | 740.5231 | -3.2410 | 0.0098 |  |
| 1-Stearoyl-2-hydroxy-sn-glycero-3-phosphoethanolamine | Glycerophospholipids | 3.1 | 20.2782 | 504.3054 | [M+Na]^+^ | 504.3066 | -2.3795 | 0.0364 |  |
| 1-(9Z-Octadecenoyl)-sn-glycero-3-phosphocholine | Glycerophospholipids | 3.3 | 19.6912 | 560.3103 | [M+K]^+^ | 560.3118 | -2.6771 | 0.0311 |  |
| N-Oleoyl-D-erythro-sphingosylphosphorylcholine (N-(9Z-Octadecenoyl)-sphing-4-enine-1-phosphocholine | Glycerophospholipids | 3.3 | 25.8107 | 729.5891 | [M+H]^+^ | 729.5911 | -2.7413 | 0.0491 | **** |
| Lyso-PC(16:0) (1-Palmitoyl-sn-glycero-3-phosphocholine) | Glycerophospholipids | 3.4 | 23.9181 | 496.3405 | [M+H]^+^ | 496.3403 | 0.4029 | 0.0464 |  |
| 1-(1Z-Octadecenyl)-sn-glycero-3-phosphocholine | Glycerophospholipids | 3.7 | 20.0562 | 508.3752 | [M+H]^+^ | 508.3768 | -3.1473 | 0.0308 |  |
| 4-[5-[[4-[5-[Acetyl(hydroxy)amino]pentylamino]-4-oxobutanoyl]-hydroxyamino]pentylamino]-4-oxobutanoic acid | Fatty Acyls | 3.8 | 18.7200 | 478.2922 | [M+NH4]^+^ | 478.2880 | 8.7813 | 0.0359 |  |
| 1-Lignoceroyl-2-hydroxy-sn-glycero-3-phosphocholine | Glycerophospholipids | 4.0 | 24.6565 | 608.4639 | [M+H]^+^ | 608.4656 | -2.7939 | 0.0423 |  |
| 1-(9Z-Octadecenoyl)-sn-glycero-3-phosphocholine | Glycerophospholipids | 4.3 | 19.6927 | 522.3546 | [M+H]^+^ | 522.3560 | -2.6802 | 0.0311 |  |
| 2-Linoleoyl-1-palmitoyl-sn-glycero-3-phosphoethanolamine | Glycerophospholipids | 4.6 | 26.4412 | 716.5215 | [M+H]^+^ | 716.5231 | -2.2330 | 0.0072 |  |
| 1-Hexadecanoyl-2-(9Z-octadecenoyl)-sn-glycero-3-phosphoethanolamine (1-Palmitoyl-2-oleoyl-sn-glycero-3-phosphoethanolamine) | Glycerophospholipids | 4.8 | 26.9539 | 718.5363 | [M+H]^+^ | 718.5389 | -3.6185 | 0.0062 |  |
| Norchenodeoxycholic acid | Steroids and steroid derivatives | 4.9 | 15.4634 | 361.2734 | [M-H2O+H]^+^ | 361.2742 | -2.2144 | 0.0092 |  |
| PAF (Platelet Activating Factor) | Glycerophospholipids | 5.1 | 20.5720 | 524.3703 | [M+H]^+^ | 524.3717 | -2.6699 | 0.0225 |  |
| 1-Octadecanoyl-sn-glycero-3-phosphocholine | Glycerophospholipids | 5.5 | 20.5674 | 546.3521 | [M+Na]^+^ | 546.3536 | -2.7455 | 0.0218 |  |
| (2-aminoethoxy)[2-[icosa-5.8.11.14-tetraenoyloxy]-3-[octadec-11-enoyloxy]propoxy]phosphinic acid (PE(18:1(11Z)/20:4(5Z,8Z,11Z,14Z))) | Glycerophospholipids | 6.4 | 26.6177 | 766.5367 | [M+H]^+^ | 766.5388 | -2.7396 | 0.0057 |  |
| 1-Palmitoyl-2-myristoyl-sn-glycero-3-phosphocholine | Glycerophospholipids | 7.6 | 27.0665 | 706.5370 | [M+H]^+^ | 706.5388 | -2.5476 | 0.0065 |  |
| 1,2-Di-(9Z,12Z,15Z-octadecatrienoyl)-sn-glycero-3-phosphocholine (PC(18:3(9Z,12Z,15Z)/18:3(9Z,12Z,15Z))) | Glycerophospholipids | 10.5 | 26.1293 | 778.5373 | [M+H]^+^ | 778.5388 | -1.9267 | 0.0223 |  |
| 1-(9Z-Octadecenoyl)-sn-glycero-3-phosphocholine | Glycerophospholipids | 11.3 | 19.0046 | 544.3388 | [M+Na]^+^ | 544.3380 | 1.4697 | 0.0138 |  |
