# Supplementary material for: Restorative potential of (−)-epicatechin in a rat model of Gulf War illness muscle atrophy and fatigue
Source: Sci Rep. 2021 Nov 8;11:21861. doi: 10.1038/s41598-021-01093-w (PMC8575952; doi:10.1038/s41598-021-01093-w)
Supplement: Supplementary file 2 — Supplementary Figures. [file 41598_2021_1093_MOESM2_ESM.pdf]

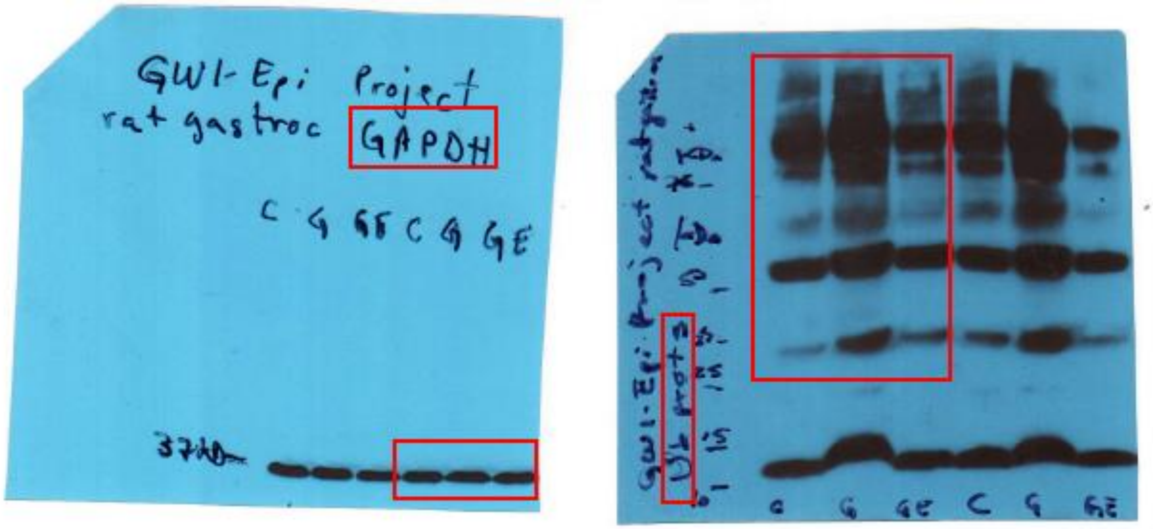

Supplementary Figure 1. Western Blot in **figure 3** illustrates total protein ubiquitylation and GAPDH as loading control in skeletal muscle (gastrocnemius) of controls (C), GWI (G) and GWI -Epi treated (GE).

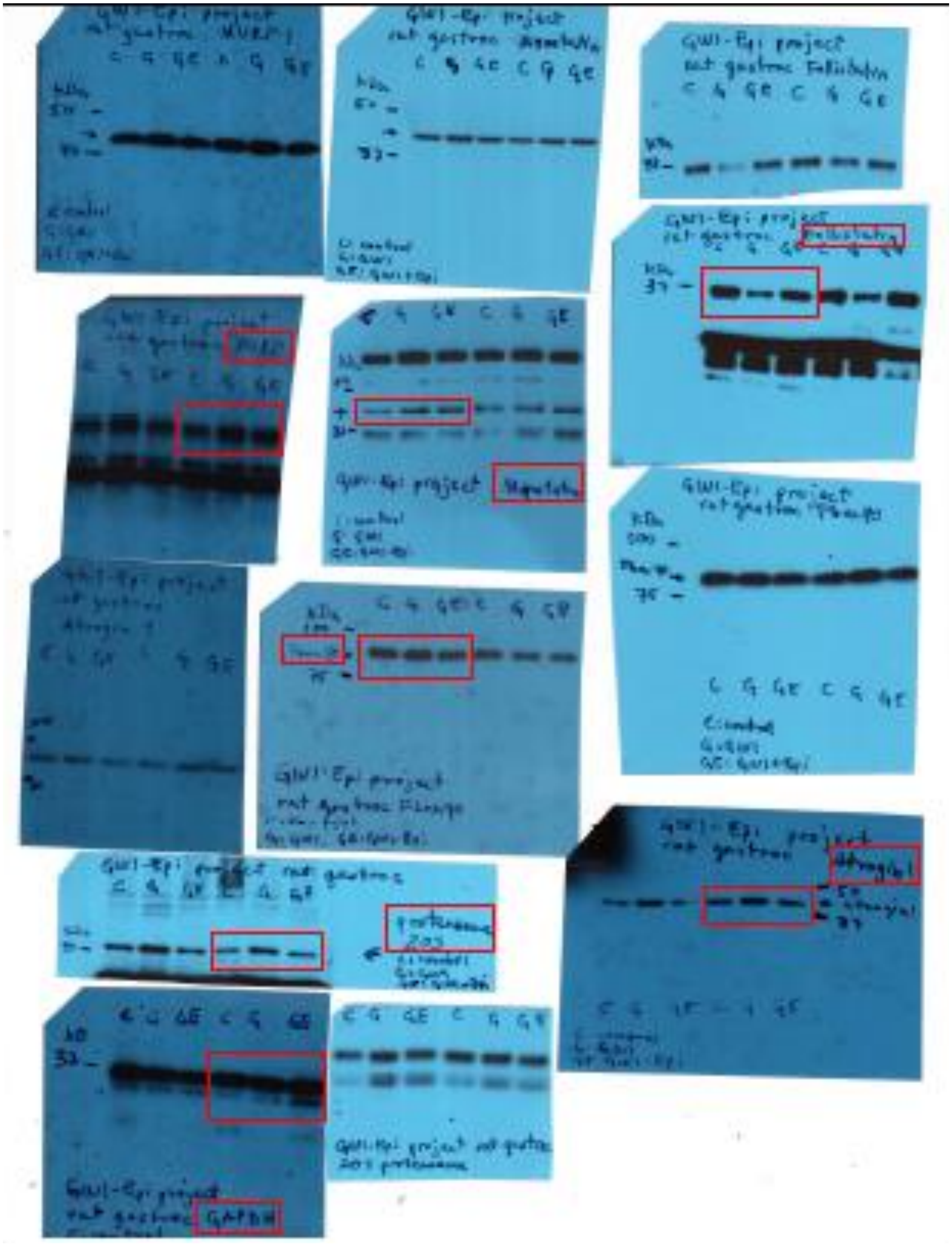

Supplementary Figure 2. Western blot in **figure 7** illustrates representative blot images of follistatin, myostatin, Murf1, Fbox40, atrogin1, proteasome subunit 20 (S20) and GAPDH used as loading control in skeletal muscle (gastrocnemius) of control (C), GWI (G) and GWI-Epi (GE) animals.

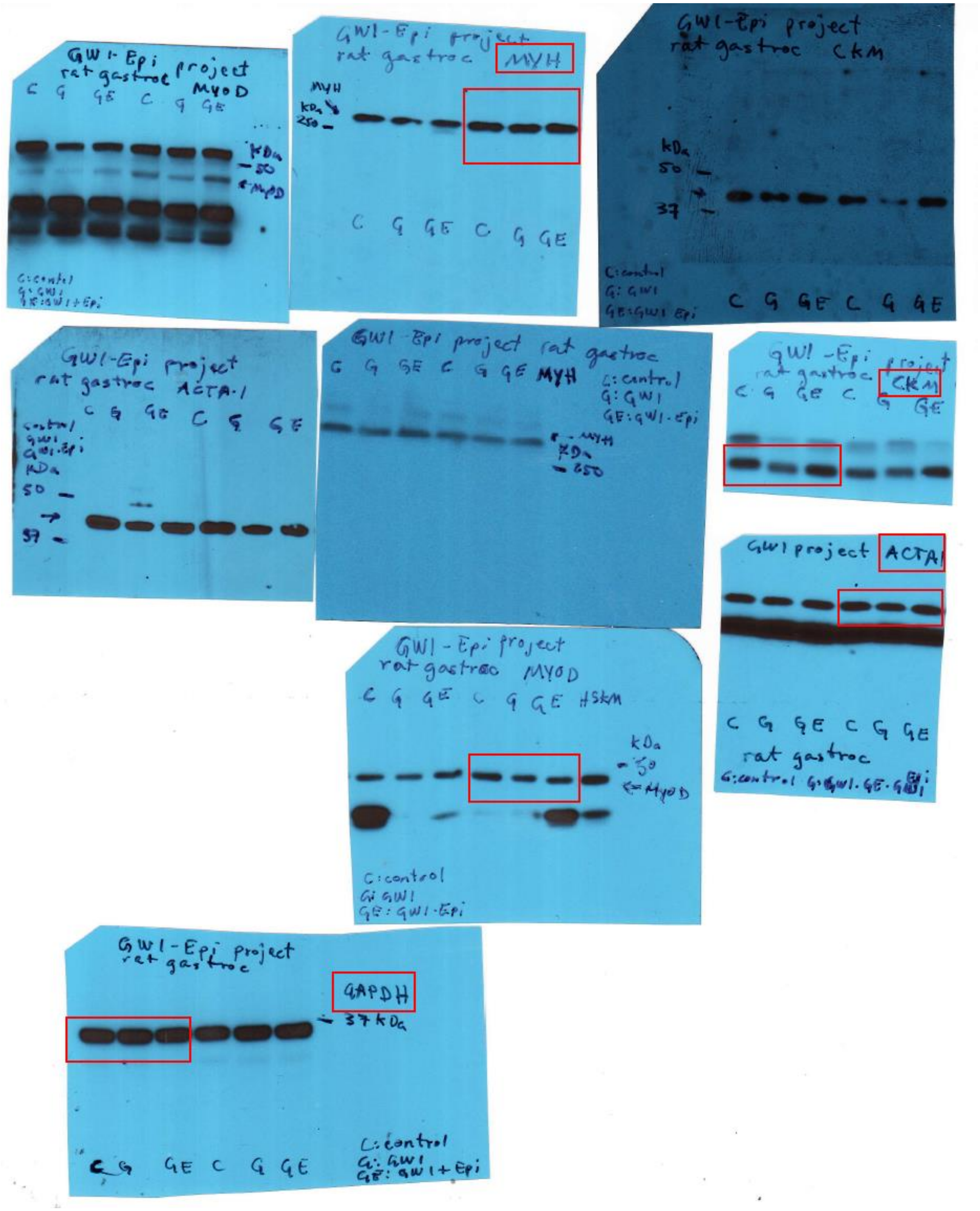

Supplementary Figure 3. Western blot in **figure 8** illustrates representative blot images of muscle creatine kinase (CkM), myosin heavy chain 2a (MYH), MyoD, and α1-actin (ACTA1) and GAPDH used as loading control in skeletal muscle (gastrocnemius) of control (C), GWI (G) and GWI-Epi (GE) animals.

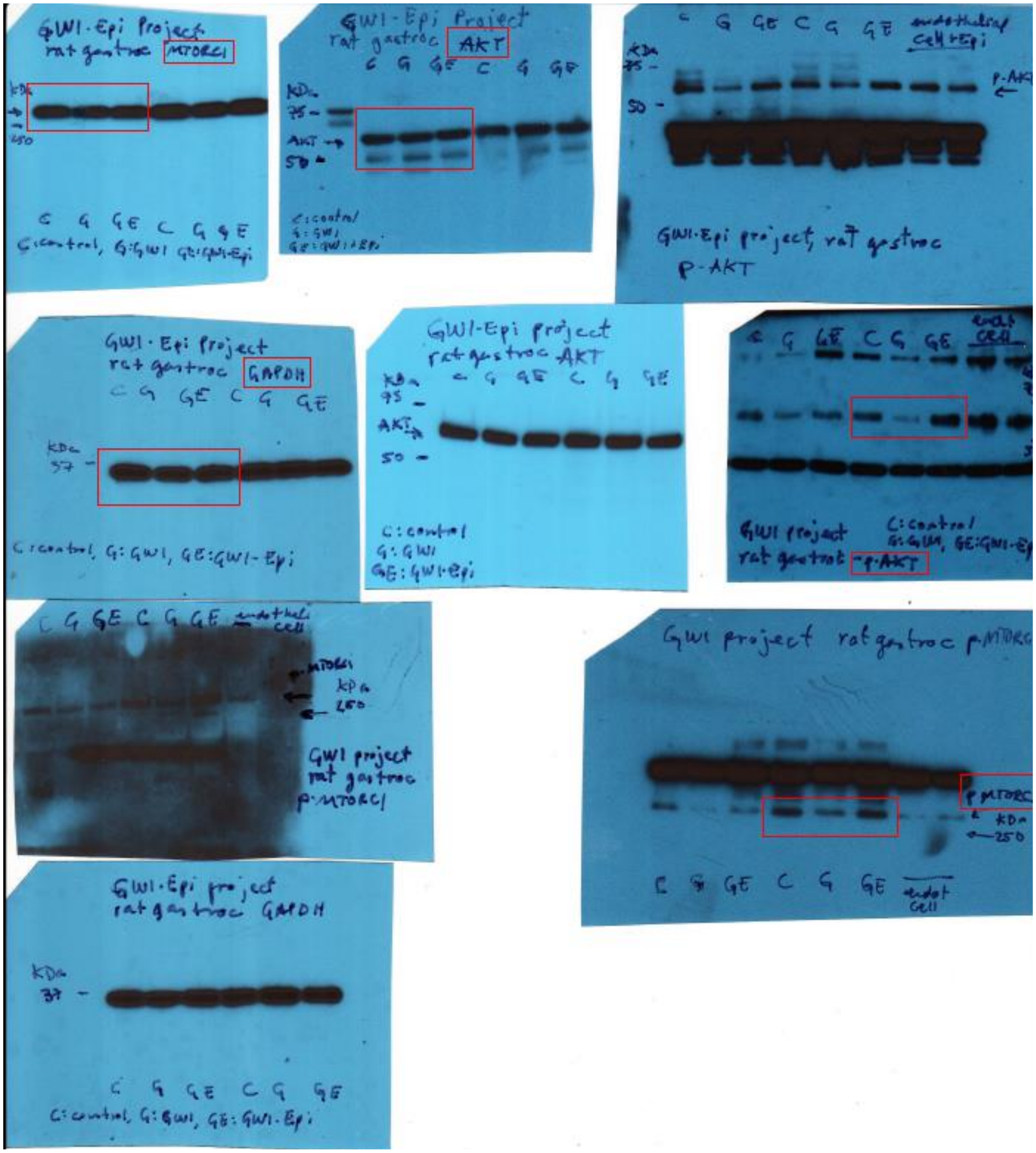

Supplementary Figure 4. Western blots in **figure 9** illustrates representative blot images of AKT, phospho-AKT (p-AKT), MTORC1, phospho MTORC1 (p-MTORC1) and GAPDH used as loading control in skeletal muscle (gastrocnemius) of control (C), GWI (G) and GWI-Epi (GE) animals.
